# ChoCallate: a computational pipeline for consensus germline variant calling in genomic sequencing data

**DOI:** 10.7717/peerj.21758

**Published:** 2026-09-29

**Authors:** Aleksey Ermolaev, Mikhail Divashuk

**Affiliations:** All-Russian Research Institute of Agricultural Biotechnology, Moscow, Russia

**Keywords:** Variant calling, Pipeline, Nextflow, Consensus calling, Voting-based consensus, Next-generation sequencing

## Abstract

Accurate germline variant calling in non-model and polyploid organisms remains challenging due to sequencing errors, genomic complexity, and caller-specific biases. Existing ensemble methods of variant calling are constrained by species-specific training, limited portability, or an inability to process multiple samples concurrently. To address these limitations we developed ChoCallate, a Nextflow-based pipeline for consensus germline variant calling. The pipeline integrates three species-independent callers, bcftools, FreeBayes, and Genome Analysis Toolkit (GATK) HaplotypeCaller, within a flexible voting mechanism. A distinctive feature of ChoCallate is its incorporation of coverage-aware consensus generation *via* Browser Extensible Data (BED) file, enabling reliable variant identification even in reduced-representation sequencing data such as Restriction site Associated DNA sequencing (RAD-seq) or RNA-seq. In a simulated dataset containing 10,000 single-nucleotide polymorphisms (SNPs) and 1,000 InDels of *Arabidopsis thaliana* across coverage depths ranging from 3× to 50×, the two-vote consensus strategy provided a favourable precision-recall balance, particularly at lower coverage (3×–10×), where SNP precision was enhanced without substantial loss of sensitivity. False-negative variants were found to concentrate in centromeric and nucleolus organizer regions (NORs) across all strategies, indicating that repetitive genomic structure, rather than consensus logic, constitutes the primary limitation. In a case study of 200 bread wheat (*Triticum aestivum*) samples, ChoCallate identified 11,178 high-confidence variants, and a genome-wide association study (GWAS) for awnedness successfully recovered the known B1/ALI-1 locus on chromosome 5A, replicating prior findings with reduced genomic inflation (*λ* (gc) = 1.06) by decreasing the number of false-positive variants through a consensus-based approach. ChoCallate is portable, species-agnostic, and freely available.

## Introduction

High-throughput sequencing has transformed the study of genomic variation, yet the accurate identification of germline single-nucleotide variants (SNVs) and small insertions and deletions (indels) remains challenging because of sequencing and mapping errors, complex genome architecture, and heterogeneous sample properties. Calling indels is particularly challenging relative to SNVs in both germline and somatic contexts (Barbitoff et al., 2022). Benchmarking studies indicate that no single caller performs optimally across all settings; rather, performance depends on the dataset, sequencing depth, library purity, genetic admixture, and genomic context, highlighting the need to reduce caller-specific biases rather than rely on a single algorithm (Guille et al., 2025). These limitations are amplified in polyploid organisms, where subgenome homology increases error rates and can compromise downstream population-genetic inferences (Cooke, Wedge & Lunter, 2022; Phillips, 2024). Accurate variant calling is therefore essential because errors can propagate into downstream analyses, including genome-wide association studies (GWAS) and population genetics, and ultimately lead to spurious biological conclusions.

Ensemble variant calling, which combines the outputs of multiple algorithms through consensus rules, leverages complementary strengths to improve sensitivity while reducing false positives (Trevarton, Chang & Symmans, 2023). Comparative evaluations show that voting-based ensembles can outperform individual tools, including deep-learning callers (Guille et al., 2025). Evidence from diverse datasets further suggests that simple majority or N–1 consensus rules provide robust precision–recall trade-offs without extensive parameter tuning, and that the accuracy of indel detection benefits substantially from multi-caller integration because breakpoint alignments are inherently complex (Trevarton, Chang & Symmans, 2023). Indel benchmarking studies show that combining orthogonal (tools that use different underlying methods) callers offers a practical and reliable way to improve accuracy in challenging genomic regions (Gong et al., 2024).

To date, several tools have been developed for integrating multiple variant callers into a consensus framework. Variant Detective (Charron & Kang, 2024) uses a configurable consensus approach based on three variant callers for variant identification: Clair3 (Zheng et al., 2025), Genome Analysis Toolkit (GATK) HaplotypeCaller (Van der Auwera & O’Connor, 2020), and FreeBayes (Garrison & Marth, 2012). This pipeline was implemented in Python 3 and provides end-to-end processing of individual samples; however, it was designed only for haploid calling in relatively short bacterial genomes and cannot process multiple samples in a single run. Furthermore, Clair3, which relies on deep learning for variant identification, was pretrained on human sequencing data, limiting its applicability to non-model organisms. SomaticCombiner implements a consensus ensemble approach using seven callers: LoFreq (Wilm et al., 2012), MuSE (Ji et al., 2024), Mutect2 (Benjamin et al., 2019), Mutect (Cibulskis et al., 2013), Strelka (Kim et al., 2018), VarDict (Lai et al., 2016) and Varscan (Koboldt et al., 2012). However, it was designed for somatic calling, rather than germline. Therefore, there are currently no ready-to-use, portable, species-independent frameworks for ensemble germline variant identification.

We developed ChoCallate, a Nextflow-based pipeline for consensus germline variant calling that integrates species-independent callers, including bcftools, GATK HaplotypeCaller, and FreeBayes. Consensus calling is implemented through a vote-based approach, in which a variant is retained only if it is supported by a specific number of callers. Leveraging Nextflow’s modular design, ChoCallate supports flexible consensus strategies through customizable voting thresholds, user-defined ensemble configurations, and the ability to process any number of samples in a single run. A custom Python script generates the consensus output in Binary Call Format (BCF) or Variant Call Format (VCF). One distinctive feature of this script is its awareness of sufficiently covered positions, achieved through the use of a Browser Extensible Data (BED) file containing intervals with coverage depth above a specified threshold. This capability is particularly valuable for use with sparse multi-sample reduced-representation sequencing data, such as Restriction site Associated DNA sequencing (RAD-seq) or RNA-based sequencing, commonly used for non-model organisms. By producing quality-controlled SNV and indel calls, ChoCallate simplifies downstream analysis, removes the need for *ad hoc* scripting, and makes ensemble variant calling more accessible to the broader genomics community.

## Materials & Methods

### Pipeline overview and workflow

The ChoCallate pipeline is implemented using the Nextflow Domain Specific Language version 2 (DSL2) (Di Tommaso et al., 2017), with individual processing steps utilizing established bioinformatics tools and a custom Python script (Fig. 1). Core tools include Burrows-Wheeler Alignment Tool (BWA) (Li & Durbin, 2009), minimap2 (Li, 2018; Li, 2021), Bowtie2 (Langmead & Salzberg, 2012), Samtools and Bcftools (Danecek et al., 2021), Freebayes (Garrison & Marth, 2012), BEDOPS (Neph et al., 2012), GATK4 (Van der Auwera & O’Connor, 2020) and BEDtools (Quinlan & Hall, 2010). The versions of all tools used in ChoCallate are presented in Table 1. Dependency management is implemented *via* Conda environments, and consensus generation employs a custom Python script. Nextflow enables high task parallelization and portable deployment across computational platforms.

**Figure 1:**
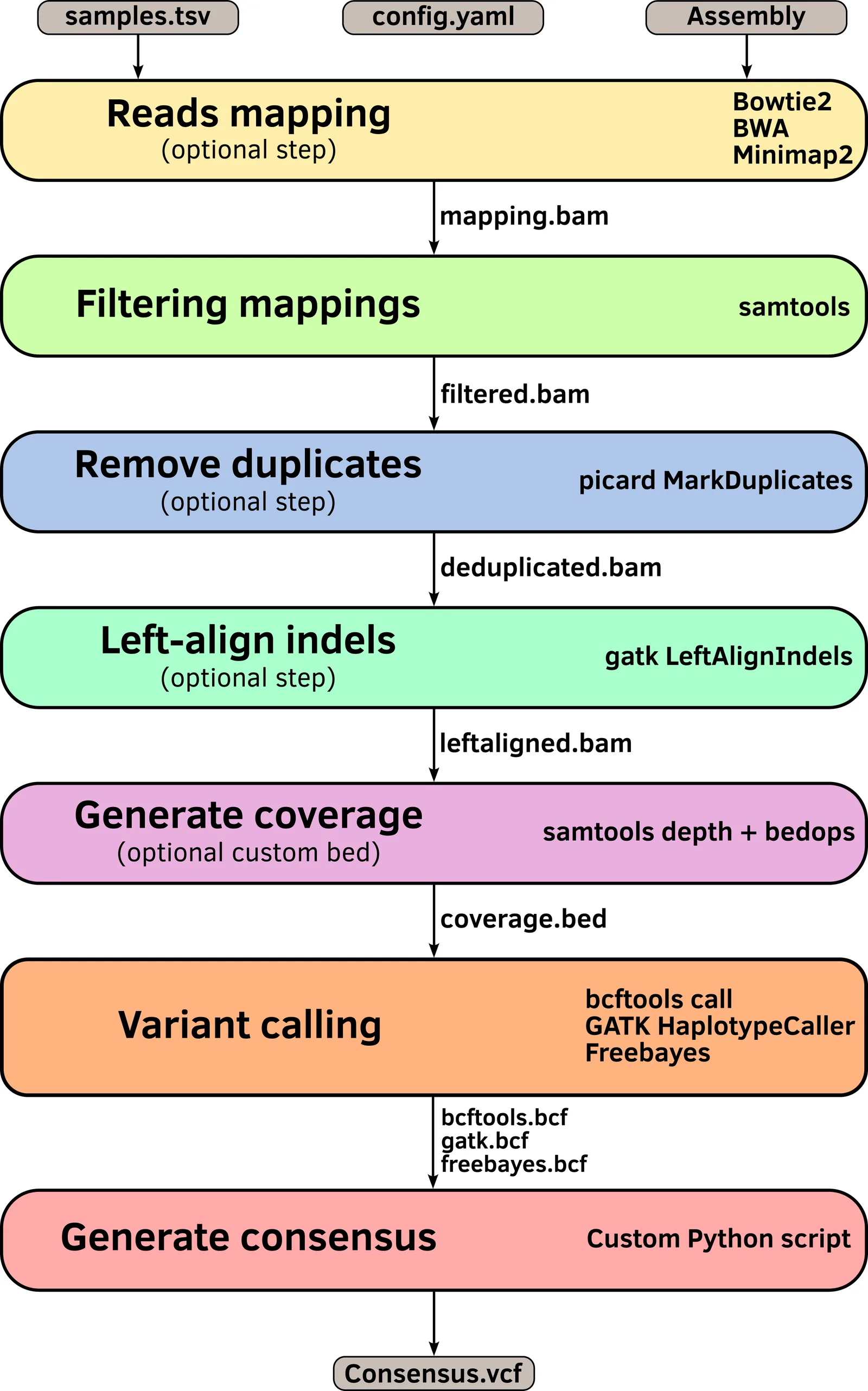
Simplified overview of the ChoCallate pipeline.

ChoCallate is configured using a YAML file. Additionally, the user must prepare a tab-delimited sample sheet with no header, containing sample names (the first column) and absolute paths to either raw reads in FASTQ format or pre-aligned BAM files, along with a reference genome assembly (gzipped or plain-text FASTA format). A detailed description of all parameters, as well as a template config file, are available in a GitHub repository (https://github.com/alermol/ChoCallate) and Zenodo (Ermolaev, 2026). A pre-built index is required for the genome assembly when the input format is FASTQ. The user can specify the mapping tool based on preference: Bowtie2 (Langmead & Salzberg, 2012), BWA (Li & Durbin, 2009) or Minimap2 (Li, 2018; Li, 2021), which will be used to map FASTQ reads. The pipeline supports mapping of paired-end (PE), single-end (SE), or hybrid (MX) reads (*i.e.,* both paired-end and single-end reads provided for a single sample). Hybrid mode is only available when Bowtie2 is the selected mapping tool, as the handling of both read types is a built-in feature of Bowtie2. Alternatively, a pre-aligned BAM file can be used as input; in this case, no mapping is performed and the BAM file is processed directly. By default, all unmapped reads are filtered from the BAM file. Duplicate removal is disabled by default, while left-alignment of indels is enabled. Left-alignment is a critical pre-processing step that ensures consistent representation of indels across different variant callers—a foundational requirement for reliable consensus variant calling.

To ensure all callers process an identical set of genomic positions, samtools and bedops are used together to generate a list of genomic intervals at which coverage depth meets or exceeds a user-specified threshold. Positions must be covered by at least 2 reads by default. This threshold was selected on the premise that for sites with low coverage depth, the calling algorithms used in the ensemble will compensate for each other’s errors, leading to better detection of variants in such positions. This BED file is also used in the consensus generation script. ChoCallate processes each sample separately but offers the option to merge samples at the end of the pipeline and output a single multisample file. Since only variable positions are included in the VCF file, the status of positions absent from the VCF cannot be reliably inferred (Table 2): a given position may either carry a reference homozygous genotype or lack sufficient read coverage entirely. The coverage BED file resolves this ambiguity: for each position in the BED file, its absence in the VCF indicates that the position is reference homozygous, while its presence in the VCF indicates a variant at that position. This approach prevents the loss of positional state information during the merging of single-sample VCFs into a single multisample file. When merging single-sample VCFs into a multisample file, bcftools assigns positions not shared between samples as either missing or reference genotypes. BED-assisted consensus VCF creation eliminates this ambiguity during multisample VCF construction, ensuring that all non-intersecting sites are designated as missing. Additionally, this approach enables the mitigation of bugs in the internal algorithm of tools that select positions based on coverage. Such an unresolved bug was previously reported for Freebayes (https://github.com/freebayes/freebayes/issues/817) and may also be present in other tools.

**Table 1:** List of versions of all tools used in ChoCallate.

**ChoCallate step**	**Tool**	**Version**
Reads mapping	BWA	0.7.19
Reads mapping	minimap2	2.30
Reads mapping	bowtie2	2.5.5
Filtering mappings	samtools	1.23
Remove duplicates	Picard Tools (MarkDuplicates)	3.4.0
Left-align indels	GATK4 (LeftAlignIndels)	4.6.2.0
Generate coverage	bedops	2.4.42
Variant calling	bcftools (mpileup + call)	1.23.1
Variant calling	GATK4 (HaplotypeCaller)	4.6.2.0
Variant calling	Freebayes	1.3.10
Generate consensus	bcftools	1.23.1

**Table 2:** Joint use of information on the presence of variants in a position from VCF and coverage depth from BED to distinguish between homozygous reference and absent variants, implemented in the ChoCallate pipeline.

**Position in VCF**	**Position in BED**	**Genotype**
+	+	Variant
-	+	RefHom

ChoCallate integrates results from three state-of-the-art germline variant callers: bcftools (Danecek et al., 2021), Freebayes (Garrison & Marth, 2012), and GATK4 (Van der Auwera & O’Connor, 2020) (Fig. 1). These tools are widely applied in both plant and human genomics research (Wu et al., 2019). All three callers are actively maintained and support large assemblies *via* Coordinate-Sorted Index (CSI) indexing for BAM and VCF/BCF files rather than Tabix index (TBI) alone, which is especially important for plant species with large genomes and long chromosomes, as TBI indices can only accommodate chromosomes no longer than 512 Mbp, while CSI supports chromosomes up to 2 Gbp. For example, in the reference genome assembly of bread wheat (*Triticum aestivum* L.) IWGSC CS RefSeq v2.1 (Zhu et al., 2021), 18 out of 21 chromosomes exceed 512 Mbp in length. The selected variant calling algorithms operate on distinct underlying principles (Table 3), and their combination can outperform any single algorithm by compensating for each other’s errors. Users can specify any combination of callers, including a single caller.

**Table 3:** Core principles of variant-calling tools used in ChoCallate.

**Caller**	**Core principle**
GATK HaplotypeCaller	Local *de novo* assembly and Bayesian scoring/genotyping
bcftools (mpileup + call)	Pileup-based model
FreeBayes	Haplotype-based Bayesian detection from aligned reads

The consensus strategy uses a voting approach. A user can specify the number of votes required to determine a variant as true: from one vote (equivalent to a simple aggregation of the callers’ results) up to three (all callers must agree). For consensus, the variants must match in coordinates, reference and alternative alleles, and genotype zygosity (GT field). Currently, other FORMAT tags, except GT, are not used for generating consensus, but it will be implemented in the future version of the pipeline. Only high-quality (QUAL≥20) variants are included in voting during consensus generation. Moreover, since each sample is processed independently, all multiallelic variants are removed, as only biallelic variants can exist in a single diploid organism, while multiallelic variants can appear only at the population level (for example, in a combined file with multiple samples). Thus, all multiallelic and low-quality variants in a single-sample VCF/BCF are considered as a reference homozygous genotype, since there is no clear evidence of a high-quality variant at this position, but there is sufficient coverage depth for calling. At present, the only tag in the consensus VCF/BCF is FORMAT/NM, which denotes the number of calling programs that agree on the genotype. For multiallelic variants in the merged VCF/BCF, the FORMAT/NM tag is provided for reference and each alternative allele. ChoCallate does not perform phasing as a post-calling processing step.

By default, ChoCallate generates a structured output directory containing a single BCF or VCF file containing consensus variants for each sample. Alternatively, pipeline can merge all per-samples BCFs/VCFs into single BCF/VCF. The output directory also contains essential log files: a main log, an error log, a pipeline summary report with execution timeline, and a process-level trace file.

Long multi-sample runs may generate numerous temporary files, potentially exhausting available disk space. To address this, all temporary files produced by each process are stored in a dedicated temporary folder within the working directory and removed upon process completion. Additionally, automatic cleanup is performed during pipeline execution *via* the Nextflow plugin nf-boost (https://github.com/bentsherman/nf-boost), which deletes temporary files (*i.e.* any files generated during processing that are not required at any of the subsequent stages and are not placed in the output directory, excluding internally generated logs and scripts) as soon as they are no longer required. Together, these measures enable multisample runs with optimal disk space utilization. One of the known limitations of using such automatic cleanup is that it interferes with Nextflow’s built-in ability to resume a pipeline from the point of interruption. This dynamic deletion of temporary files can be disabled before starting the pipeline, which restores the full ability to resume interrupted runs.

### Simulation study

We evaluated the performance of ChoCallate on a simulated dataset derived from the *Arabidopsis thaliana* TAIR12 assembly (Reiser et al., 2026). A total of 10,000 single-nucleotide polymorphisms (SNPs) and 1,000 indels were introduced at randomly selected genomic positions using simuG v1.0.1 (Yue & Liti, 2019), generating a mutated genome and the corresponding ground-truth variant set. Paired-end 250 bp reads were then simulated from the mutated genome at different coverage depths (3 ×, 5 ×, 10 ×, 30 ×, and 50 ×) using Real Time Genomics (RTG) Tools v3.13 (https://github.com/RealTimeGenomics/rtg-tools). Variants were identified with the ChoCallate pipeline using the BWA (Li & Durbin, 2009) mapper and default parameters, including coverage depth ≥2, variant QUAL≥20, disabled duplicate removal, and enabled left-alignment of indels, except for the consensus vote threshold, which was tested at thresholds of at least 1, 2, and 3 supporting votes. Each run was performed under the same limits of random access memory (RAM) and central processing units (CPUs) for each process: all available RAM (256 Gb) and 32 CPUs for mapping and consensus creation and 10 CPUs for each caller. For mapping and consensus generation all 32 CPUs were allocated, while during calling, 10 CPUs were allocated for each caller. Performance was evaluated by comparing the resulting call sets against the ground-truth variants using the vcfeval subcommand in RTG Tools v3.13. Sensitivity, precision, and F1-score were used as evaluation metrics. SNP and indels were evaluated separately. All simulated data as well as raw output files are available on Zenodo (Ermolaev, 2026). The distribution of false-negative (FN) variants across chromosomes in non-overlapping 1 Mbp windows was calculated using vcfeval output and bedtools (Quinlan & Hall, 2010). Data visialization was performed using R (R Core Team, 2025).

### Case study

We evaluated ChoCallate using a publicly available reduced-representation SNP-seq comprising 200 bread wheat samples (BioProject PRJNA1270396). Awnedness was used as the phenotypic trait for the GWAS. The analysis panel contained 200 samples, including 150 awned and 50 awnless individuals. Variants were identified with the ChoCallate pipeline using the Bowtie2 aligner and default parameters, including minimum coverage depth ≥2, variant quality *QUAL* ≥ 20, duplicate removal disabled, left alignment of indels enabled, and a consensus threshold of two votes, which had previously been identified as the optimal setting using a simulated ground-truth dataset. We allocated 10 CPUs for Bowtie2, five CPUs for each calling tool and 32 CPUs for consensus creation. Before GWAS, all multiallelic sites were split into biallelic sites, and variants were filtered based on call rate ≥95%, minor allele frequency (MAF) ≥5%, and observed heterozygosity ≤10%. We used an observed heterozygosity threshold instead of a Hardy-Weinberg equilibrium test because this sample set does not represent a natural population, but rather a collection of genetically stable varieties with, as expected, low observed heterozygosity; therefore, genotype frequencies were not expected to follow Hardy-Weinberg equilibrium. The transition/transversion (*Ts*/*Tv*) ratio was assessed both in the initial dataset and in the final dataset after filtering. The threshold for each filtering step was selected empirically from the observed pre-filtering distribution. Missing genotypes were imputed after all filtering steps using Beagle 5.5 (Browning, Zhou & Browning, 2018). Following imputation, an additional quality-control filter removed variants with MAF < 5%. This final step yielded 11,178 high-confidence polymorphic variants, including 6,861 SNPs and 4,317 indels. A set of linkage disequilibrium-pruned variants was extracted for population structure analysis using PLINK2 v2.00a6LM (Chang et al., 2015) with the *–indep-pairwise 500kb 0.5* option. The top 10 principal components (PCs) were used to account for population structure in the GWAS model. GWAS was performed using the Bayesian Information and Linkage Disequilibrium Iteratively Nested Keyway (BLINK) model (Huang et al., 2019), as implemented in the GAPIT3 package (Wang & Zhang, 2021). Previous work has shown that BLINK performs well for association mapping in wheat, as evidenced by quantile-quantile (Q-Q) plots and the recovery of simulated quantitative trait locus (QTL) across GWAS models (Sandhu et al., 2024). GWAS results were visualised in R v4.3.1. The significance threshold for GWAS was determined using a Bonferroni correction at a significance level of 0.01, yielding a threshold defined as 0.01/11,178 =8.95 × 10^−7^. Genomic inflation of *P*-values was evaluated using the genomic inflation factor *λ* and a Q-Q plot.

### Hardware and benchmark environment

All benchmarks were executed inside a Kernel-based Virtual Machine (KVM) (Proxmox VE 8.4.16) running on a dedicated host with two AMD EPYC 7742 (64 physical cores/128 threads per socket, Zen 2 microarchitecture). The virtual machine (VM) was allocated 32 virtual CPUs (vCPUs). CPU frequency was locked at the base frequency of 2.0 GHz. The VM has 256 Gb of DDR4-3200 RAM. Data on VM was stored on 2 Tb virtual hard disk drive. The guest operating system was Debian 12 Bookworm (kernel 6.1.0-28-amd64).

## Results

### Simulated dataset validation

To evaluate the performance of the ensemble variant-calling approach implemented in ChoCallate, we benchmarked it using a synthetic ground-truth dataset comprising 10,000 SNPs and 1,000 indels introduced at random positions in the *Arabidopsis thaliana* TAIR12 assembly (Reiser et al., 2026). Variant calling was performed at five average coverage depths (ACDs): 50 ×, 30 ×, 10 ×, 5 ×, and 3 ×. The results are presented in Figs. 2A–2H and Table S1. At high ACDs (50 × and 30 ×), nearly all approaches, including single-caller and consensus ones, showed similar precision, recall, F1 score, and total numbers of detected SNPs and indels. At intermediate and lower ACDs (10 ×, 5 ×, and 3 ×), differences between single-caller and consensus approaches became more apparent.

**Figure 2:**
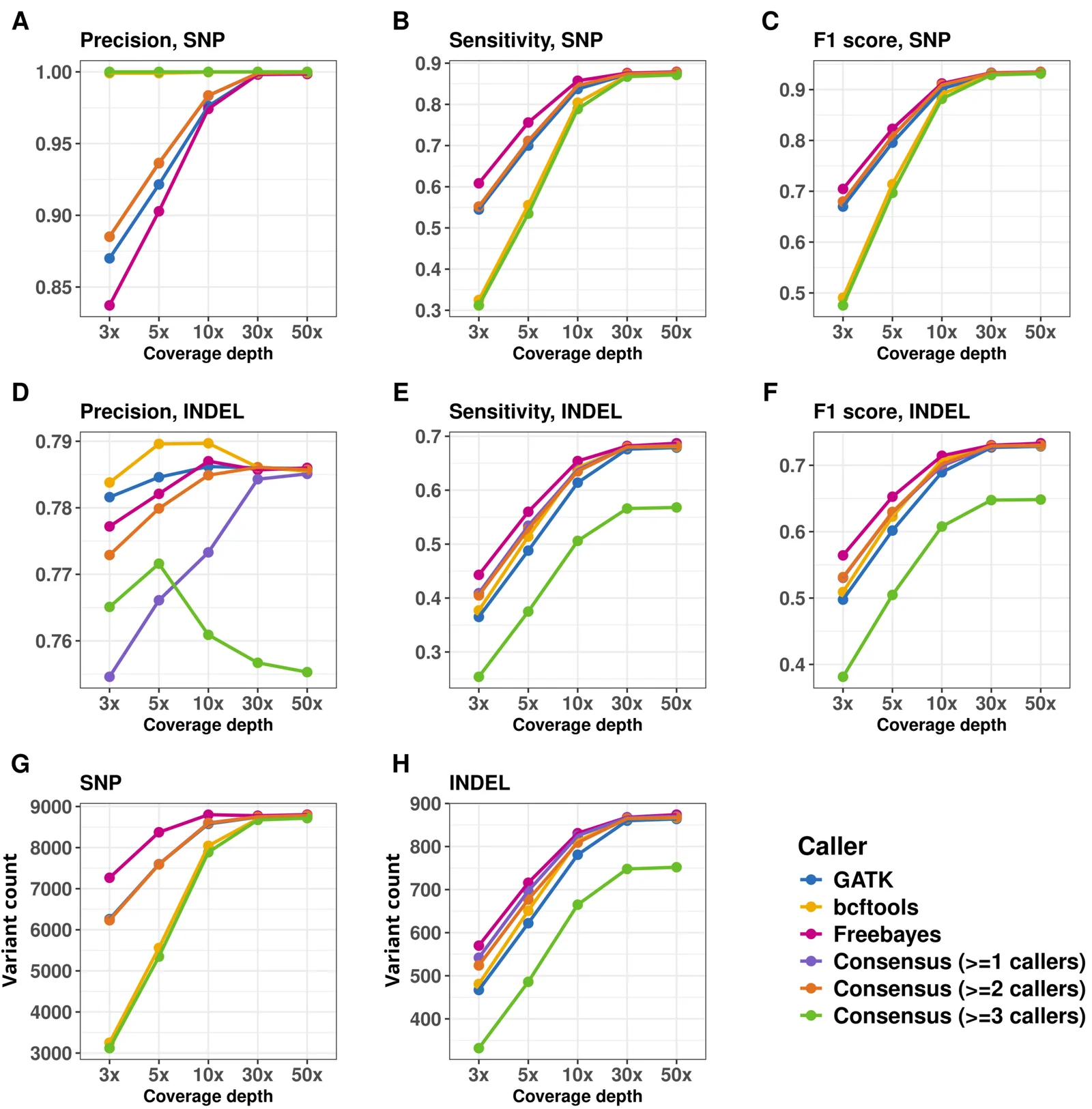
Performance comparison of variant-calling tools used in the pipeline, including GATK4, bcftools, and FreeBayes, under different voting-consensus thresholds and coverage depths. The top row shows precision (A), sensitivity (B), and F1 score (C) for SNPs across different sequencing coverage depths. The middle row shows precision (D), sensitivity (E), and F1 score (F) for indels across different sequencing coverage depths. The bottom row shows the numbers of SNPs (G) and indels (H) detected across different sequencing coverage depths.

For SNP detection, decreasing ACD led to reductions in precision, sensitivity, F1 score, and the number of identified SNPs. At low ACDs (5 × and 3 ×), Freebayes outperformed all other single callers and consensus approaches in the number of detected SNPs, sensitivity, and F1 score. However, the two-votes consensus approach outperformed Freebayes and GATK in precision (Fig. 2A). Notably, the precision of bcftools and the three-votes approach remained nearly ideal (∼1.0) across all ACDs (Fig. 2A). However, their sensitivity and numbers of detected SNPs declined more sharply than those of the other callers and consensus approaches (Figs. 2B, 2G), reflecting the conservative nature of both the three-votes approach and bcftools (Pinto, Sousa & Silva, 2026). In other words, these methods report variants only when the evidence is exceptionally clear: most reported calls are correct, but many true variants fail to pass the filters.

For indel detection, individual callers, especially FreeBayes, achieve comparable or superior precision and recall relative to all consensus strategies at the coverage depths tested. Among consensus strategies, the three-votes consensus showed lower performance than all other approaches across almost all ACDs (Figs. 2D–2F). For the remaining approaches, performance metrics were again nearly identical at high ACDs (50 × and 30 ×) and began to diverge at lower ACDs (10 ×, 5 ×, and 3 ×). bcftools achieved the highest precision for indel calling among both single callers and consensus approaches (Fig. 2D); however, it underperformed in sensitivity and in the number of detected indels. Among the consensus approaches, the two-votes strategy provided the best precision at lower ACDs (10 ×, 5 ×, and 3 ×), while maintaining nearly identical sensitivity and numbers of identified indels (Figs. 2E, 2H).

We evaluated the distribution of FN variants, defined as variants present in the ground-truth dataset but not identified by the caller, across the chromosomes of *A. thaliana* in non-overlapping 1 Mbp windows. Major peaks of FN variants were detected at centromeric coordinates on all chromosomes (Naish et al., 2021) and at the ends of chromosomes 2 and 4, which harbor the nucleolus organizer regions (NORs) (Naish et al., 2021) (Fig. 3, Fig. S1). These regions are enriched in tandem repeats, which make reads more difficult to place and align and make variants harder to represent consistently; consequently, true variants are more likely to be missed than confidently called. The numbers of FN SNPs in these repetitive regions were very similar across individual callers and consensus approaches. The full-consensus strategy (≥3 matching calls) and bcftools at 3 × ACD produced the highest numbers of FN SNPs across chromosomes, whereas the two-vote consensus substantially reduced the number of FN SNPs. For indels, we observed a similar chromosomal distribution of FNs, and all approaches, including single callers and consensus strategies, performed in a broadly similar manner (Fig. S1).

**Figure 3:**
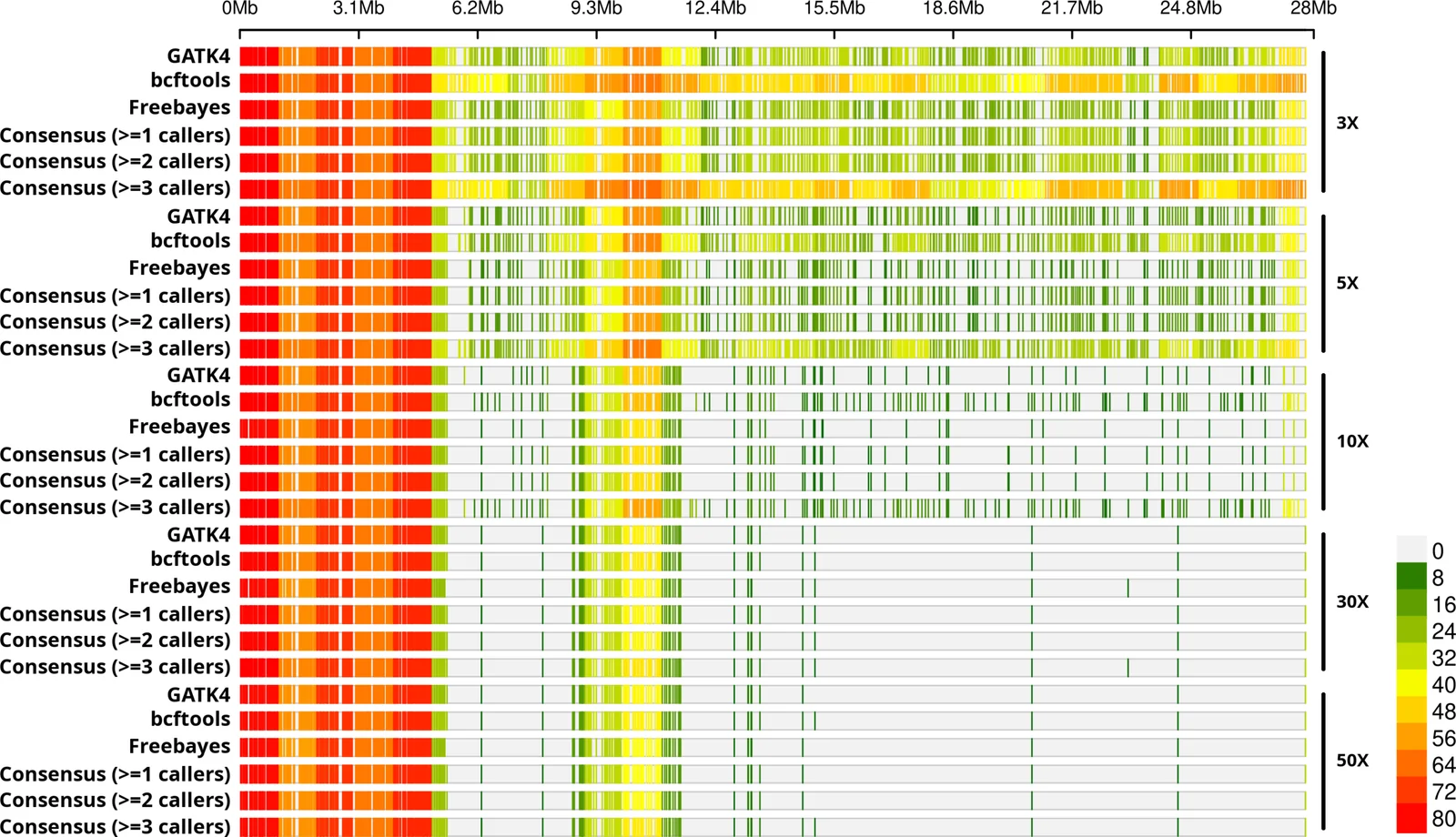
The distribution of false-negative SNPs across 1 Mbp non-overlapping windows on chromosome 2 of *A. thaliana* is shown for various coverage depths and calling strategies; distributions for all chromosomes appear in Fig. S1.

### Case study validation

To assess whether consensus output of ChoCallate can discover biologically meaningful variants, we applied it to a public dataset of 200 bread wheat samples (Bazhenov et al., 2025) and performed a GWAS of awnedness, defined as the presence or absence of awns. The run required approximately 12 h and reached a peak memory use of 35 GB during the merging of single-sample outputs into a multi-sample file. The complete output of this run, including the configuration file, log files, and consensus output, is available on Zenodo (Ermolaev, 2026). A summary of the number of variants after each filtering stage is presented in Table S2. The initial post-consensus call set comprised 57,226 variants, including 36,125 SNPs and 21,101 indels, with a *Ts*/*Tv* ratio of 1.68. After removing variants with missingness greater than 5% (Fig. 4A), 36,102 variants remained, including 21,196 SNPs and 14,933 indels, with a *Ts*/*Tv* ratio of 1.78. In the next step, variants with low MAF (<5% ) were removed. Such variants typically provide limited statistical power and can yield unstable effect estimates when the minor allele is present in only a few samples. They are also more likely to reflect genotyping or imputation noise, which may lead to false-positive associations. After removing low-MAF and monomorphic variants, 14,544 variants remained, including 8,496 SNPs and 6,048 indels, with a *Ts*/*Tv* ratio of 1.81 (Fig. 4B). The final filtering step was based on the distribution of observed heterozygosity (Fig. 4C). Because this dataset consists of genetically stable varieties, sites with high observed heterozygosity were expected to reflect systematic calling errors. Based on the empirical distribution, we set the filtering threshold at 10%. Sites with observed heterozygosity ≥10% were removed, leaving 11,179 variants, including 6,861 SNPs and 4,318 indels, with a *Ts*/*Tv* ratio of 1.77. This slight decrease in the *Ts*/*Tv* likely reflects the polyploid nature of the bread wheat genome, which contains numerous highly similar homoeologous regions. Such regions can increase mapping ambiguity and compromise variant calling accuracy, potentially resulting in spurious heterozygous genotype calls at sites that are in fact homozygous. This variant set was then used for genotype imputation, followed by an additional round of low-MAF filtering. The final high-quality dataset contained 11,178 variants, including 6,861 SNPs and 4,317 indels, with a *Ts*/*Tv* ratio of 1.77. The increase in the *Ts*/*Tv* ratio from the initial to the final dataset suggests that the filtering strategy successfully enriched the call set for cleaner and more reliable variants, making it better suited for downstream association analysis.

**Figure 4:**
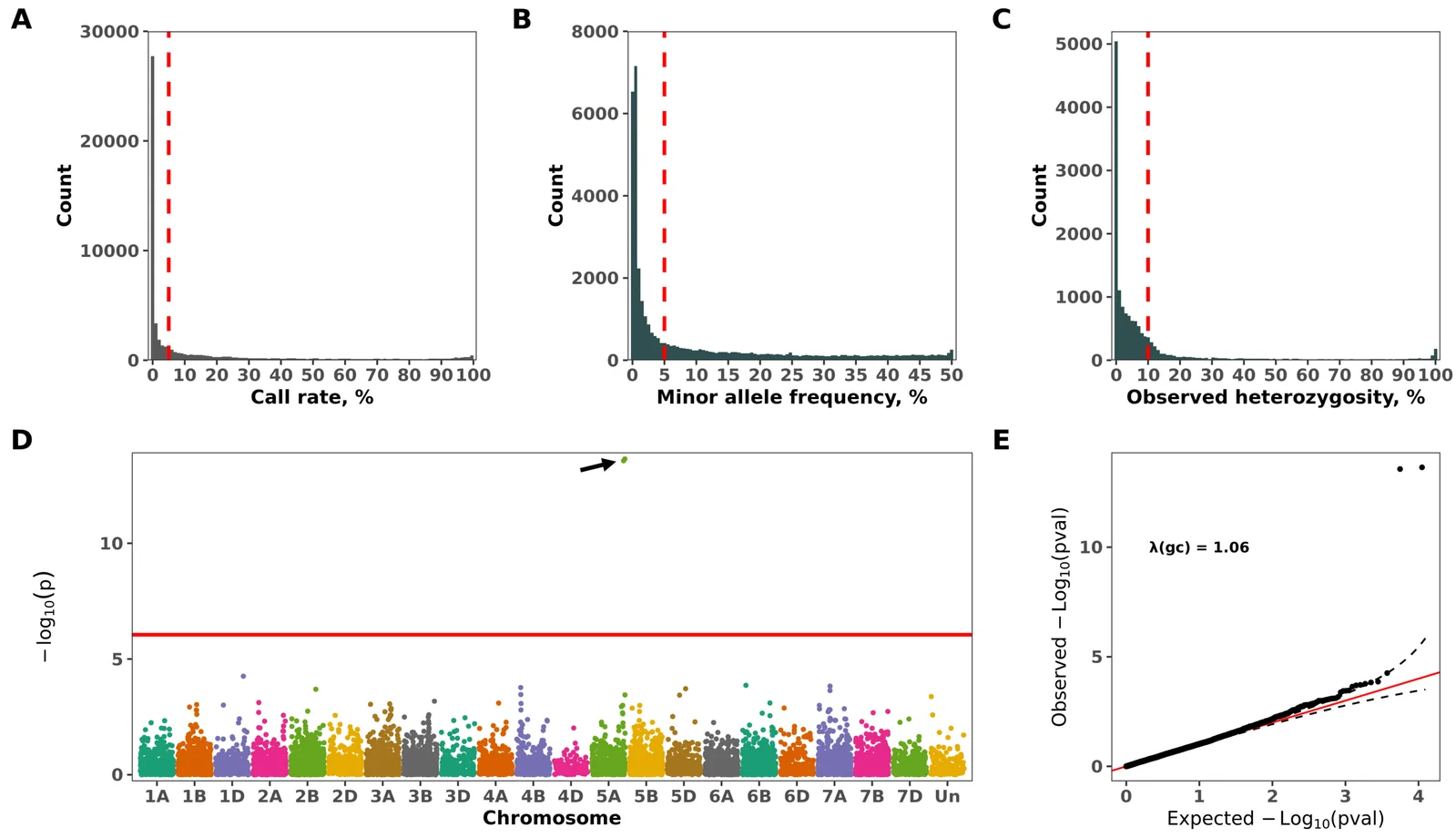
Genome-wide association study (GWAS) for awnedness. Top row: Empirical distributions of variant call rate (A), minor allele frequency (B), and observed heterozygosity (C) used for quality filtering of consensus calling prior to GWAS. Red dashed lines indicate the threshold applied at each filtering step. Bottom row: Results of the GWAS. (D) shows a Manhattan plot of the association between genetic variants identified by ChoCallate and the awnedness phenotype. Significant associations were detected on chromosome 5A. The horizontal red line denotes the genome-wide significance threshold determined by Bonferroni correction at a significance level of 0.01 (8.95 × 10^−7^). The black arrow marks variants with statistically significant association. (E) presents a quantile–quantile (Q-Q) plot of observed *versus* expected −*log*_10_ (*P*-values). The genomic inflation factor *λ*_*gc*_ exhibits a mild elevation (*λ*_*gc*_ = 1.06), likely attributable to imbalance in the phenotypic categories (150 awned and 50 awnless samples).

GWAS using the BLINK model identified a genomic locus significantly associated with awnedness on chromosome 5A (Fig. 4D, Table 4). The Q-Q plot showed close agreement between the observed *P*-values and the null distribution, indicating that the model adequately accounted for population structure and minimised spurious associations (Fig. 4E). Mild inflation of the *P*-value distribution, reflected in a slightly elevated genomic inflation factor (*λ*_*gc*_ = 1.06), is likely attributable to the imbalance between the phenotypic categories (150 awned and 50 awnless samples) and the polyploid nature of the bread wheat genome. Notably, the genomic inflation factor in our analysis was closer to 1, indicating less deviation from the null expectation, than that reported in the original study (*λ*_*gc*_ = 0.921, indicating deflation). Our analysis identified two variants associated with awnedness in bread wheat (Table 4). These variants are located near those reported in the original study (Bazhenov et al., 2025) and fall within the previously reported major quantitative trait locus B1/ALI-1, which is associated with awns suppression (DeWitt et al., 2020). Variant 5A:698003176 (S5A_698003176, DeWitt et al., 2020) was previously reported as a SNP significantly associated with awn suppression (DeWitt et al., 2020). Other SNPs reported in that study were absent from the consensus set before filtering and were not covered by sequencing reads. All SNPs reported by Bazhenov et al. (2025) were detected and included in the initial consensus dataset; however, 5A:698510016 was removed because of its low MAF (4%), and 5A:708290964 did not reach the significance threshold in the GWAS. Furthermore, the variant 5A:708290964 (Bazhenov et al., 2025) lies outside the boundaries of the previously reported B1/ALI-1 QTL (spanning from 681,455,268 bp to 706,705,101 bp; DeWitt et al., 2020) and may therefore represent a false-positive association.

**Table 4:** Comparison of marker-trait associations for awnedness identified by ChoCallate *versus* the original study (Bazhenov et al., 2025). The ChoCallate variant-calling pipeline recovered a previously published locus for awnedness, and the statistically significant SNPs detected were near previously reported markers.

**Chromosome**	**Position**	**Ref**	**Alt**	**Source**
5A	698,003,176	T	G	This study; DeWitt et al. (2020)
5A	698,510,016	T	C	Bazhenov et al. (2025)
5A	706,654,773	G	C	This study
5A	708,290,964	T	C	Bazhenov et al. (2025)

## Discussion

Our benchmarking indicates that the main limitation of variant recovery in ChoCallate is not the consensus procedure itself, but the genomic context in which variants occur. Across callers and voting thresholds, false-negative variants accumulated primarily in centromeric and NOR-associated regions (Fig. 3, Fig. S1), where repetitive sequence increases mapping ambiguity and makes true variants more difficult to align and represent consistently. The broadly similar distribution of FN calls among single callers and consensus strategies (Fig. S1) suggests that these missed variants arise mainly from shared limitations of short-read mapping in repetitive DNA rather than from the consensus design itself.

The simulation also shows that the value of consensus calling depends strongly on sequencing depth (Fig. 2). At high ACDs (50 ×, 30 ×), most approaches performed similarly for both SNPs and indels, indicating that when read support is abundant, the choice between single-caller and consensus strategies has relatively little effect on overall accuracy. In contrast, at moderate and low ACDs (10 ×, 5 ×, and 3 ×), differences between approaches became much more pronounced. Under these conditions, the two-vote consensus provided a more favourable balance between precision and sensitivity. Compared with more permissive single-caller outputs, it improved precision, while avoiding the substantial loss of sensitivity and variant yield observed for the three-vote consensus and, for SNPs, for bcftools. At low average coverage depths (ACD ≤10 ×), the recall of bcftools and the three-vote strategy declines so sharply that the resulting marker sets may be inadequate for downstream GWAS and population-genetic analyses. Conversely, while FreeBayes achieves higher F1 at low ACD, its lower precision introduces false-positive variants that can generate spurious associations in GWAS, with potentially misleading biological consequences. These results indicate that agreement between any two of three callers is sufficiently stringent to remove many low-confidence calls, but not so strict that large numbers of true variants are discarded.

Indel-detection performance showed a pattern distinct from that of SNP calling, warranting careful interpretation. At lower ACDs (≤10 ×), individual callers, particularly FreeBayes, achieve precision and recall equal to or exceeding those of the consensus strategies. Notably, the three-vote consensus shows the lowest indel precision across most conditions, which is the opposite of what might be expected from a stringent filtering strategy. This counterintuitive result likely reflects the fact that all three callers rely on the same underlying read alignments: systematic alignment artefacts near repetitive sequences or complex structural regions can produce characteristic false indel signals that all three callers independently report, resulting in high-confidence but spurious consensus calls. The two-vote strategy partially mitigates this by requiring agreement between only two callers, but indel precision under this strategy still does not consistently exceed that of FreeBayes alone. These findings indicate that the value of consensus calling is less pronounced for indels than for SNPs. For applications where indel accuracy is the primary objective and sequencing depth is sufficient, single-caller strategies, especially FreeBayes, may be preferred. The principal advantage of the two-vote consensus for indel detection lies in its robustness across varied coverage conditions, rather than in absolute precision improvement.

Together, the benchmarks on the simulated dataset indicate that the two-vote consensus provided a consistent precision-oriented profile across all tested coverage depths, without requiring dataset-specific tuning of the calling strategy. By contrast, the ranking of individual callers, particularly in terms of recall and F1, varied with coverage depth, so that no single caller dominated across all conditions. This relative robustness to coverage variation represents the practical argument for a consensus default in heterogeneous datasets. For downstream GWAS and population-genetic analyses, where false positives create spurious associations and bias summary statistics, minimising the false-discovery rate is the appropriate objective. For applications where recall is the primary objective, such as variant discovery in well-covered whole-genome data, users may prefer individual callers or a one-vote threshold, which ChoCallate also supports.

The wheat case study shows that the consensus output remains biologically informative in a much more complex genome. Successive filtering steps reduced missingness, removed low-frequency and high-heterozygosity sites, and increased the overall *Ts*/*Tv* ratio from the initial to the final dataset, consistent with progressive enrichment for higher-confidence variants. The two-vote consensus helped exclude potential false-positive association signals while retaining biologically meaningful candidate variants. Based on simulations using a dataset with known ground truth, it can reasonably be argued that applying a stricter consensus with a three-vote threshold would reduce the final set and may exclude some true-positive associations, whereas using a more liberal threshold of one-vote would increase the set of candidates, but could also raise the rate of false-positive associations. The final call set from two-vote consensus supported a well-calibrated GWAS and recovered a significant association on chromosome 5A (Fig. 4) within the previously described B1/ALI-1 QTL (DeWitt et al., 2020) controlling awn suppression. Importantly, the pipeline not only retained a known biological signal, but also identified significant variants close to previously reported markers. It should be noted that alternative consensus thresholds (one-vote and three-vote) were not quantitatively benchmarked in the wheat case study itself; therefore, this analysis should be interpreted as a demonstration of the biological feasibility of the two-vote consensus rather than as a formal benchmark of threshold performance in a polyploid genome.

From a practical perspective, these findings support the two-vote consensus as the most suitable default setting for ChoCallate. It performs comparably to single callers at high depth, improves robustness at low depth, and avoids the excessive conservatism of requiring complete agreement across callers. More broadly, our results suggest that in plant resequencing datasets with heterogeneous coverage depth, the principal obstacle to comprehensive variant discovery is not consensus filtering per se, but the persistent difficulty of recovering variants from repetitive and poorly mappable regions. For this reason, ChoCallate is likely to be especially useful for applications such as RAD-seq, low-pass resequencing, and other datasets with uneven depth, where a balance between call reliability and variant retention is essential.

Several limitations of the present study should be acknowledged. First, the benchmarking was conducted on simulated data from a single diploid model organism (*Arabidopsis thaliana*) under idealised conditions (no adapter contamination, uniform library composition, fixed read length). Performance on more complex genomes, including allopolyploids such as bread wheat, where subgenome homology substantially increases false-positive and FN rates, was assessed only through a qualitative case study, not through a ground-truth comparison. Second, the appropriate balance between precision and recall is inherently application-dependent: analyses prioritising marker discovery (*e.g.*, population diversity studies) may favour higher recall, while those prioritising association mapping quality may favour higher precision. ChoCallate supports flexible voting thresholds precisely to allow users to adjust this balance; the two-vote default represents a reasonable starting point for noisy reduced-representation data, but should not be treated as universally optimal. Finally, all callers used in ChoCallate (bcftools, GATK HaplotypeCaller, FreeBayes) rely on short-read alignments, and their consensus does not overcome the fundamental limitations of short-read mapping in repetitive and structurally complex regions, as evidenced by the clustering of false negatives in centromeric and NOR-associated regions across all strategies.

## Conclusions

We developed ChoCallate, a Nextflow-based pipeline for flexible, user-friendly consensus variant calling of germline SNVs and indels. Designed as an end-to-end solution, ChoCallate is particularly valuable for researchers in population genetics, functional genomics, and evolutionary biology working with both plant and animal species. Its Nextflow implementation ensures portability across diverse computational infrastructures, automated task parallelization, and minimal user installation and configuration. The pipeline natively processes genomic data while generating separate BCF or VCF files for SNVs and indels either separately for each sample or merged into a single multi-sample file. Simulation analyses demonstrated that a two-votes consensus strategy optimizes the precision-recall trade-off, especially at low coverage depths (3 ×–10 ×). FN variants consistently clustered in repetitive genomic regions, indicating that alignment limitations, not consensus logic, remain the primary constraint. Application of ChoCallate to a bread wheat GWAS successfully replicated a known locus for awnedness (B1/ALI-1), confirming its ability to generate high-quality variants for downstream analysis. Future developments will incorporate additional read mapping and variant calling tools, including tools specialised for long-read and polyploid variant calling, such as Octopus (Cooke, Wedge & Lunter, 2021), and implement machine learning-based post-calling filtering, such as FVC (Ren et al., 2022) of variants instead of hard filters in order to reduce the loss of true variants. A systematic comparison of downstream GWAS outcomes, including locus recovery, genomic inflation, and false-positive association rates, across individual callers and consensus thresholds on datasets with known causal variants would provide direct empirical evidence for the practical benefits of consensus calling.

## Supplemental Information

10.7717/peerj.21758/supp-1 Supplemental Information 1 Performance metrics (variant count, precision, recall and F1) of the ChoCallate pipeline on simulated dataset

10.7717/peerj.21758/supp-2 Supplemental Information 2 Variant count after each filtering step for real wheat calling data

10.7717/peerj.21758/supp-3 Supplemental Information 3 The distribution of false negative SNPs and indels across 1 Mbp non-overlapping windows across *A. thaliana* chromosomes Each figure presents the distribution of false negative SNPs and indels across 1 Mbp non-overlapping windows across *A. thaliana* chromosomes using a unique combination of single-caller and consensus approaches, as well as at a moderate coverage depth. The data for ChoCallate evaluation were simulated using a reference dataset.

## Additional Information and Declarations

### Data Availability

The following information was supplied regarding data availability: The source code for the pipeline is available on GitHub and Zenodo: - https://github.com/alermol/ChoCallate
- Aleksey Ermolaev. (2026). alermol/ChoCallate: 3.0.0 (Version v3.0.0) [Computer software]. Zenodo. https://doi.org/10.5281/zenodo.22127900
The raw phenotype data is provided as supplementary information in the associated article: - https://link.springer.com/article/10.1186/s12870-025-07322-y#Sec33
- https://doi.org/10.1186/s12870-025-07322-y
The simulated data for pipeline evaluation and raw output are available at Zenodo: - Ermolaev, A. (2026). Validation data for ChoCallate: A Computational Pipeline for Consensus Germline Variant Calling in Genomic Sequencing Data (Version 1.0.1) [Dataset]. Zenodo. https://doi.org/10.5281/zenodo.19609127
The raw sequence data are available at NCBI BioProject: PRJNA1270396.

## References

[ref-1] Barbitoff YA, Abasov R, Tvorogova VE, Glotov AS, Predeus AV (2022). Systematic benchmark of state-of-the-art variant calling pipelines identifies major factors affecting accuracy of coding sequence variant discovery. BMC Genomics.

[ref-2] Bazhenov M, Bespalova L, Samarina M, Polevikova N, Agaeva E, Debeliy A, Beliakova A, Ponisko A, Nazarova L, Chernook A, Karlov G, Divashuk M (2025). Genome-wide association study of agronomic traits in winter wheat (*Triticum aestivum* L.) using a custom SNP marker set. BMC Plant Biology.

[ref-3] Benjamin D, Sato T, Cibulskis K, Getz G, Stewart C, Lichtenstein L (2019). Calling somatic SNVs and indels with Mutect2. BioRxiv.

[ref-4] Browning BL, Zhou Y, Browning SR (2018). A one-penny imputed genome from next-generation reference panels. The American Journal of Human Genetics.

[ref-5] Chang CC, Chow CC, Tellier LC, Vattikuti S, Purcell SM, Lee JJ (2015). Second-generation PLINK: rising to the challenge of larger and richer datasets. Gigascience.

[ref-6] Charron P, Kang M (2024). VariantDetective: an accurate all-in-one pipeline for detecting consensus bacterial SNPs and SVs. Bioinformatics.

[ref-7] Cibulskis K, Lawrence MS, Carter SL, Sivachenko A, Jaffe D, Sougnez C, Gabriel S, Meyerson M, Lander ES, Getz G (2013). Sensitive detection of somatic point mutations in impure and heterogeneous cancer samples. Nature Biotechnology.

[ref-8] Cooke DP, Wedge DC, Lunter G (2021). A unified haplotype-based method for accurate and comprehensive variant calling. Nature Biotechnology.

[ref-9] Cooke DP, Wedge DC, Lunter G (2022). Benchmarking small-variant genotyping in polyploids. Genome Research.

[ref-10] Danecek P, Bonfield JK, Liddle J, Marshall J, Ohan V, Pollard MO, Whitwham A, Keane T, McCarthy SA, Davies RM, Li H (2021). Twelve years of SAMtools and BCFtools. GigaScience.

[ref-11] DeWitt N, Guedira M, Lauer E, Sarinelli M, Tyagi P, Fu D, Hao Q, Murphy JP, Marshall D, Akhunova A, Jordan K, Akhunov E, Brown-Guedira G (2020). Sequence-based mapping identifies a candidate transcription repressor underlying awn suppression at the *B1* locus in wheat. New Phytologist.

[ref-12] Di Tommaso P, Chatzou M, Floden EW, Barja PP, Palumbo E, Notredame C (2017). Nextflow enables reproducible computational workflows. Nature Biotechnology.

[ref-13] Ermolaev A (2026). Validation data for ChoCallate: a computational pipeline for consensus germline variant calling in genomic sequencing data. Zenodo.

[ref-14] Garrison E, Marth G (2012). Haplotype-based variant detection from short-read sequencing.

[ref-15] Gong B, Li D, Zhang Y, Kusko R, Lababidi S, Cao Z, Chen M, Chen N, Chen Q, Chen Q, Dai J, Gan Q, Gao Y, Guo M, Hariani G, He Y, Hou W, Jiang H, Kushwaha G, Li J-L, Li J, Li Y, Liu L-C, Liu R, Liu S, Meriaux E, Mo M, Moore M, Moss TJ, Niu Q, Patel A, Ren L, Saremi NF, Shang E, Shang J, Song P, Sun S, Urban BJ, Wang D, Wang S, Wen Z, Xiong X, Yang J, Yin L, Zhang C, Zhang R, Bhandari A, Cai W, Eterovic AK, Megherbi DB, Shi T, Suo C, Yu Y, Zheng Y, Novoradovskaya N, Sears RL, Shi L, Jones W, Tong W, Xu J (2024). Extend the benchmarking indel set by manual review using the individual cell line sequencing data from the Sequencing Quality Control 2 (SEQC2) project. Scientific Reports.

[ref-16] Guille A, Adélaïde J, Finetti P, Andre F, Birnbaum D, Mamessier E, Bertucci F, Chaffanet M (2025). A benchmarking study of individual somatic variant callers and voting-based ensembles for whole-exome sequencing. Briefings in Bioinformatics.

[ref-17] Huang M, Liu X, Zhou Y, Summers RM, Zhang Z (2019). BLINK: a package for the next level of genome-wide association studies with both individuals and markers in the millions. Gigascience.

[ref-18] Ji S, Zhu T, Sethia A, Wang W (2024). Accelerated somatic mutation calling for whole-genome and whole-exome sequencing data from heterogenous tumor samples. Genome Research.

[ref-19] Kim S, Scheffler K, Halpern AL, Bekritsky MA, Noh E, Källberg M, Chen X, Kim Y, Beyter D, Krusche P, Saunders CT (2018). Strelka2: fast and accurate calling of germline and somatic variants. Nature Methods.

[ref-20] Koboldt DC, Zhang Q, Larson DE, Shen D, McLellan MD, Lin L, Miller CA, Mardis ER, Ding L, Wilson RK (2012). VarScan 2: somatic mutation and copy number alteration discovery in cancer by exome sequencing. Genome Research.

[ref-21] Lai Z, Markovets A, Ahdesmaki M, Chapman B, Hofmann O, McEwen R, Johnson J, Dougherty B, Barrett JC, Dry JR (2016). VarDict: a novel and versatile variant caller for next-generation sequencing in cancer research. Nucleic Acids Research.

[ref-22] Langmead B, Salzberg SL (2012). Fast gapped-read alignment with Bowtie 2. Nature Methods.

[ref-23] Li H (2018). Minimap2: pairwise alignment for nucleotide sequences. Bioinformatics.

[ref-24] Li H (2021). New strategies to improve minimap2 alignment accuracy. Bioinformatics.

[ref-25] Li H, Durbin R (2009). Fast and accurate short read alignment with Burrows–Wheeler transform. Bioinformatics.

[ref-26] Naish M, Alonge M, Wlodzimierz P, Tock AJ, Abramson BW, Schmücker A, Mandáková T, Jamge B, Lambing C, Kuo P, Yelina N, Hartwick N, Colt K, Smith LM, Ton J, Kakutani T, Martienssen RA, Schneeberger K, Lysak MA, Berger F, Bousios A, Michael TP, Schatz MC, Henderson IR (2021). The genetic and epigenetic landscape of the *Arabidopsis* centromeres. Science.

[ref-27] Neph S, Kuehn MS, Reynolds AP, Haugen E, Thurman RE, Johnson AK, Rynes E, Maurano MT, Vierstra J, Thomas S, Sandstrom R, Humbert R, Stamatoyannopoulos JA (2012). BEDOPS: high-performance genomic feature operations. Bioinformatics.

[ref-28] Phillips AR (2024). Variant calling in polyploids for population and quantitative genetics. Applications in Plant Sciences.

[ref-29] Pinto V, Sousa L, Silva C (2026). Variant calling in genomics: a comparative performance analysis and decision guide. PLOS ONE.

[ref-30] Quinlan AR, Hall IM (2010). BEDTools: a flexible suite of utilities for comparing genomic features. Bioinformatics.

[ref-31] R Core Team (2025). R: a language and environment for statistical computing. https://www.r-project.org.

[ref-32] Reiser L, Proia A, Bakker E, Subramaniam S, Khosa K, Sawant S, Chen X, Prithvi T, Berardini TZ (2026). Recent major changes to TAIR: updates to the database, website, and *Arabidopsis* genome. Genetics.

[ref-33] Ren Y, Kong Y, Zhou X, Genchev GZ, Zhou C, Zhao H, Lu H (2022). FVC as an adaptive and accurate method for filtering variants from popular NGS analysis pipelines. Communications Biology.

[ref-34] Sandhu KS, Burke AB, Merrick LF, Pumphrey MO, Carter AH (2024). Comparing performances of different statistical models and multiple threshold methods in a nested association mapping population of wheat. Frontiers in Plant Science.

[ref-35] Trevarton AJ, Chang JT, Symmans WF (2023). Simple combination of multiple somatic variant callers to increase accuracy. Scientific Reports.

[ref-36] Van der Auwera GA, O’Connor BD (2020). Genomics in the cloud: using Docker, GATK, and WDL in Terra.

[ref-37] Wang J, Zhang Z (2021). GAPIT version 3: boosting power and accuracy for genomic association and prediction. Genomics, Proteomics & Bioinformatics.

[ref-38] Wilm A, Aw PPK, Bertrand D, Yeo GHT, Ong SH, Wong CH, Khor CC, Petric R, Hibberd ML, Nagarajan N (2012). LoFreq: a sequence-quality aware, ultra-sensitive variant caller for uncovering cell-population heterogeneity from high-throughput sequencing datasets. Nucleic Acids Research.

[ref-39] Wu X, Heffelfinger C, Zhao H, Dellaporta SL (2019). Benchmarking variant identification tools for plant diversity discovery. BMC Genomics.

[ref-40] Yue J-X, Liti G (2019). simuG: a general-purpose genome simulator. Bioinformatics.

[ref-41] Zheng Z, Yu X, Chen L, Lee Y-L, Xin C, Wong AOK, Jain M, Kesharwani RK, Sedlazeck FJ, Luo R (2025). Clair3-RNA: a deep learning-based small variant caller for long-read RNA sequencing data. Nature Communications.

[ref-42] Zhu T, Wang L, Rimbert H, Rodriguez JC, Deal KR, De Oliveira R, Choulet F, Keeble-Gagnère G, Tibbits J, Rogers J, Eversole K, Appels R, Gu YQ, Mascher M, Dvorak J, Luo M-C (2021). Optical maps refine the bread wheat *Triticum aestivum* cv. Chinese Spring genome assembly. The Plant Journal.

