# Supplementary material for: ChoCallate: a computational pipeline for consensus germline variant calling in genomic sequencing data
Source: PeerJ. 2026 Sep 29;14:e21758. doi: 10.7717/peerj.21758 (PMC13632679; doi:10.7717/peerj.21758)
Supplement: Supplementary file 1 [file peerj-14-21758-s003.pdf]

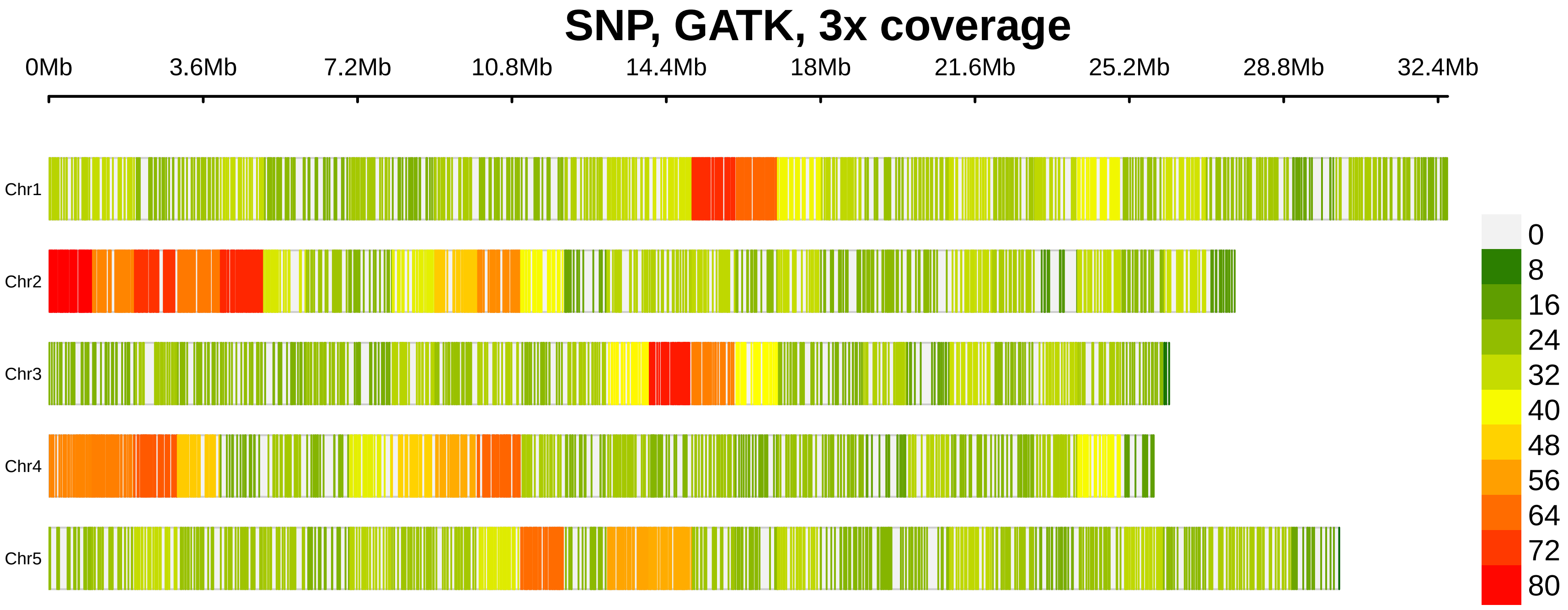
SNP, GATK, 3x coverage
0Mb
3.6Mb
7.2Mb
10.8Mb
14.4Mb
18Mb
21.6Mb
25.2Mb
28.8Mb
32.4Mb
Chr1
Chr2
Chr3
Chr4
Chr5
0
8
16
24
32
40
48
56
64
72
80

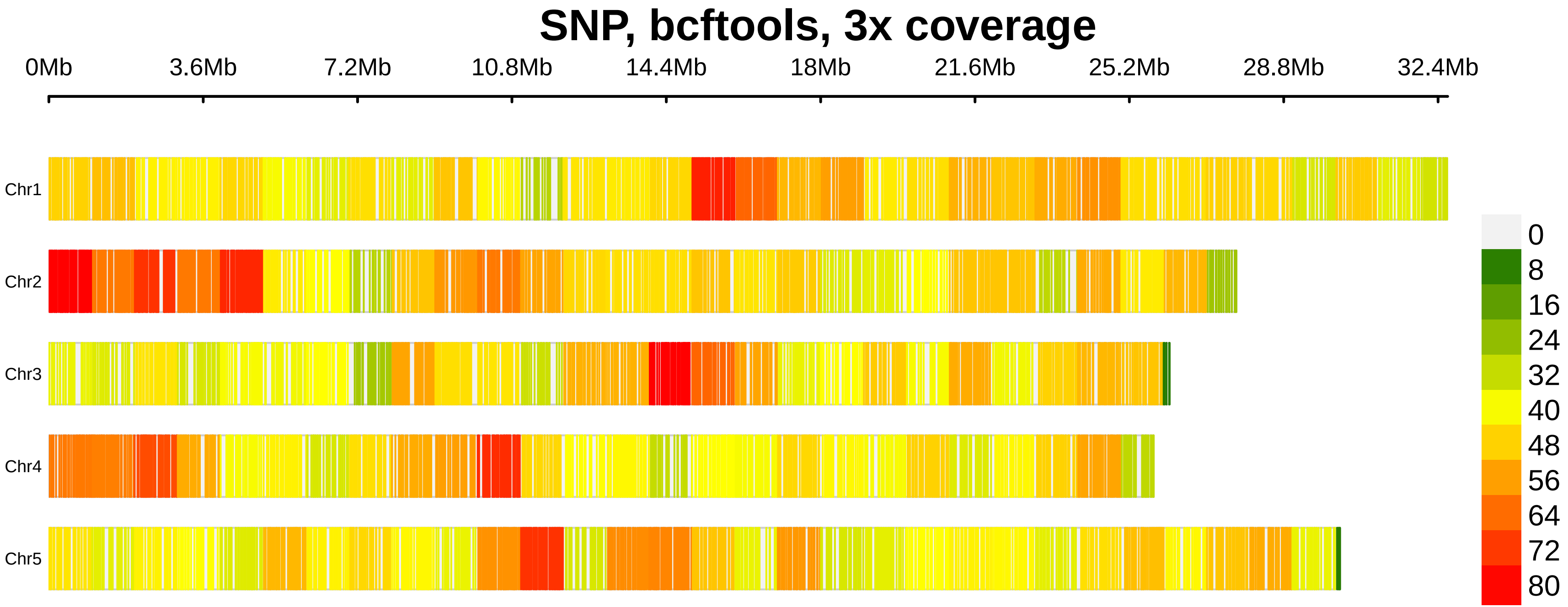

SNP, bcftools, 3x coverage
0Mb
3.6Mb
7.2Mb
10.8Mb
14.4Mb
18Mb
21.6Mb
25.2Mb
28.8Mb
32.4Mb
Chr1
Chr2
Chr3
Chr4
Chr5
0
8
16
24
32
40
48
56
64
72
80

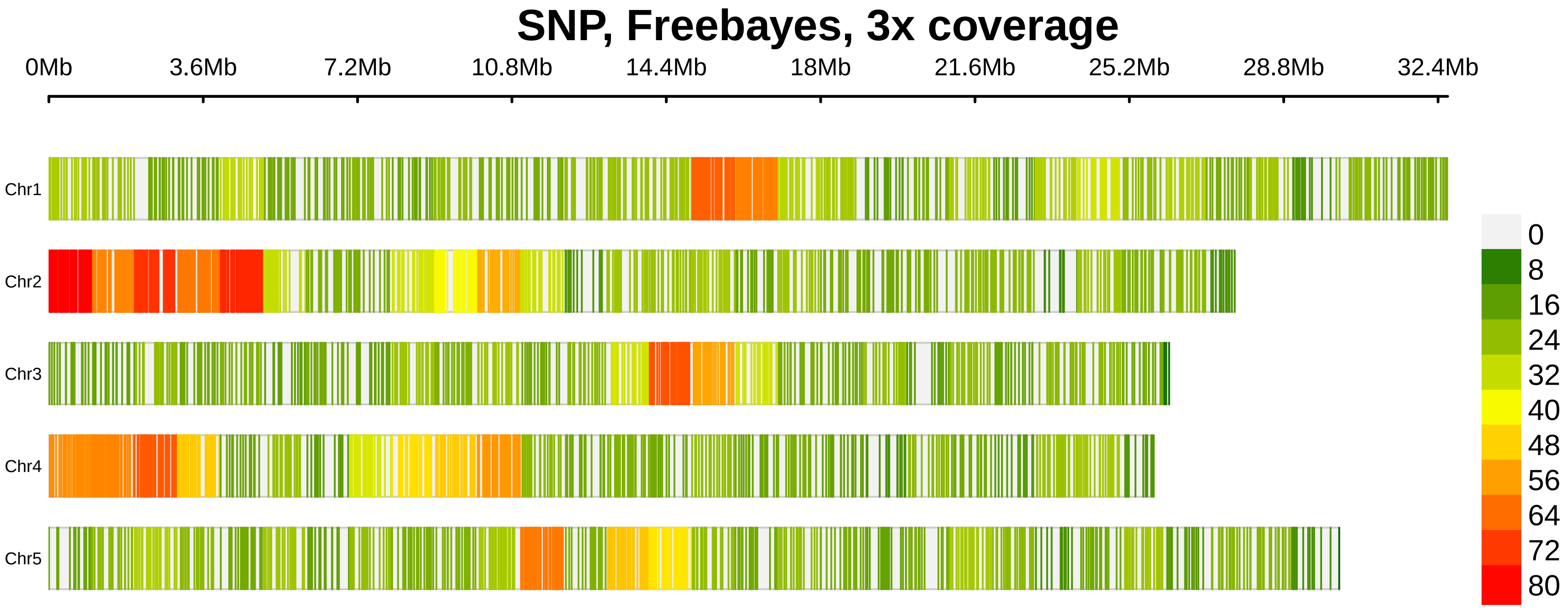
SNP, Freebayes, 3x coverage
0Mb
3.6Mb
7.2Mb
10.8Mb
14.4Mb
18Mb
21.6Mb
25.2Mb
28.8Mb
32.4Mb
Chr1
Chr2
Chr3
Chr4
Chr5
0
8
16
24
32
40
48
56
64
72
80

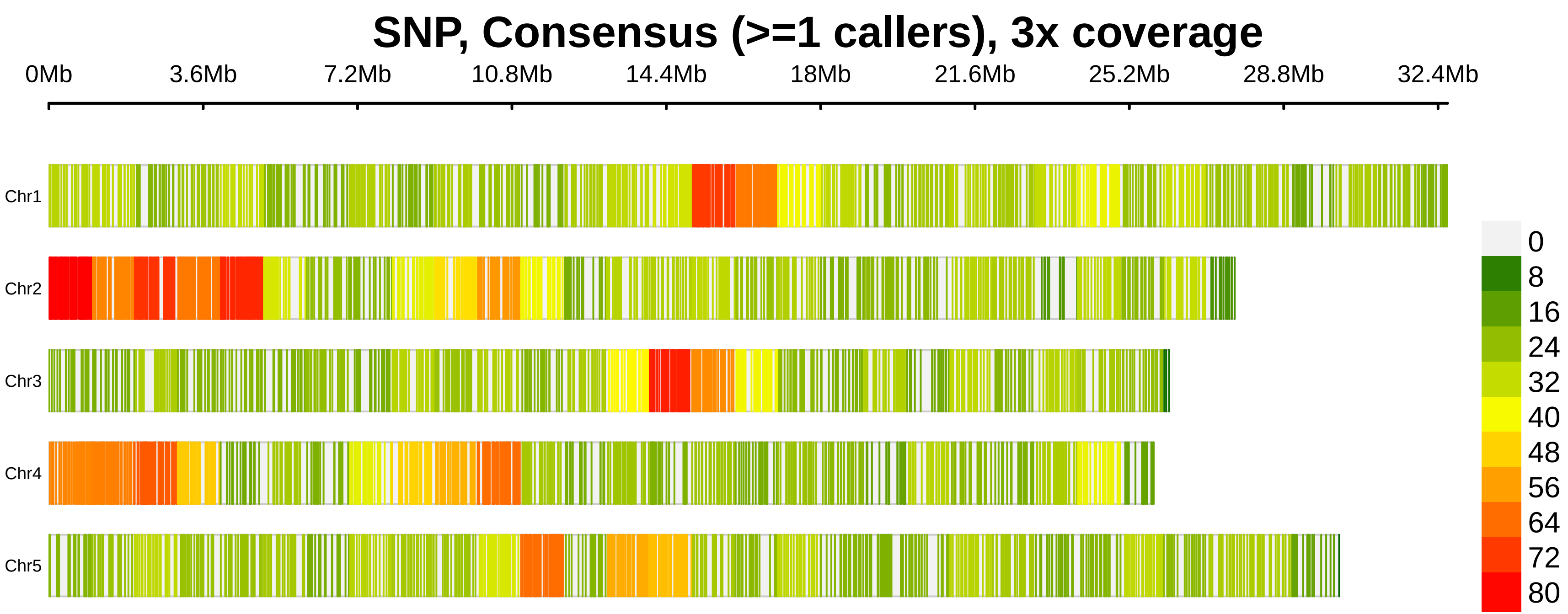
SNP, Consensus (>=1 callers), 3x coverage
0Mb
3.6Mb
7.2Mb
10.8Mb
14.4Mb
18Mb
21.6Mb
25.2Mb
28.8Mb
32.4Mb
Chr1
Chr2
Chr3
Chr4
Chr5
0
8
16
24
32
40
48
56
64
72
80

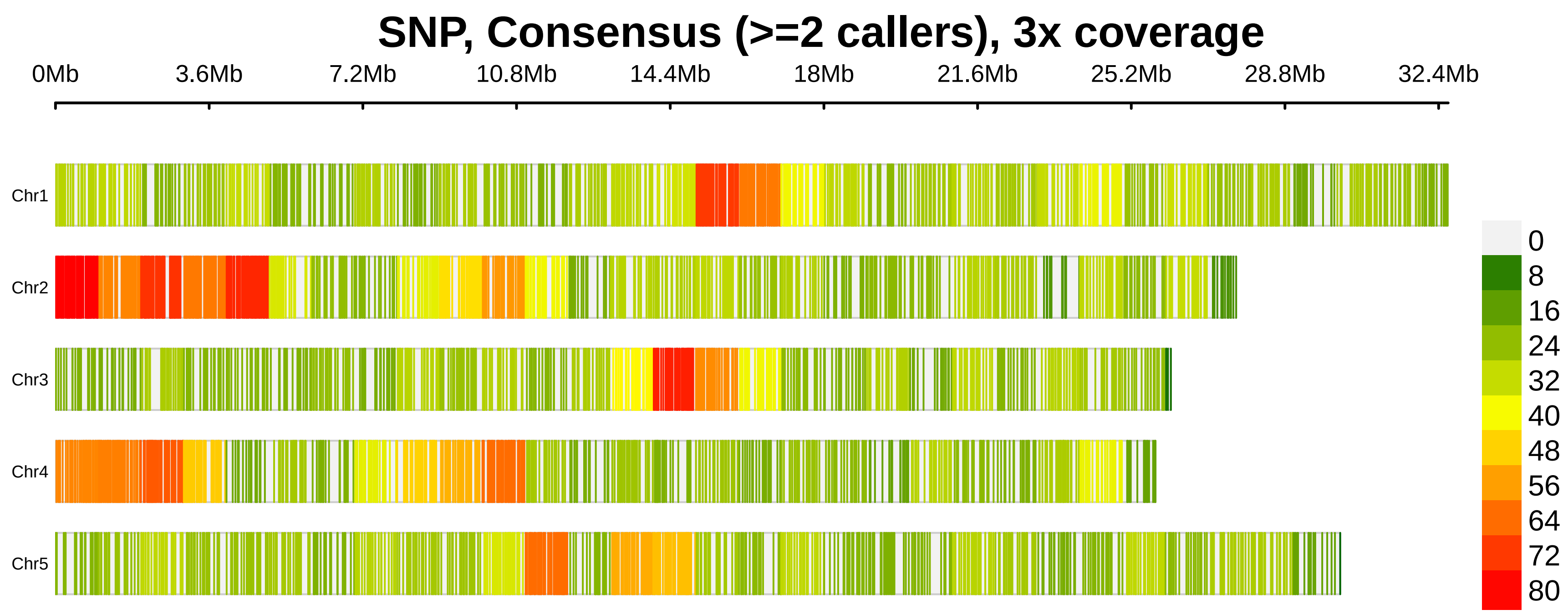
SNP, Consensus (>=2 callers), 3x coverage
0Mb
3.6Mb
7.2Mb
10.8Mb
14.4Mb
18Mb
21.6Mb
25.2Mb
28.8Mb
32.4Mb
Chr1
Chr2
Chr3
Chr4
Chr5
0
8
16
24
32
40
48
56
64
72
80

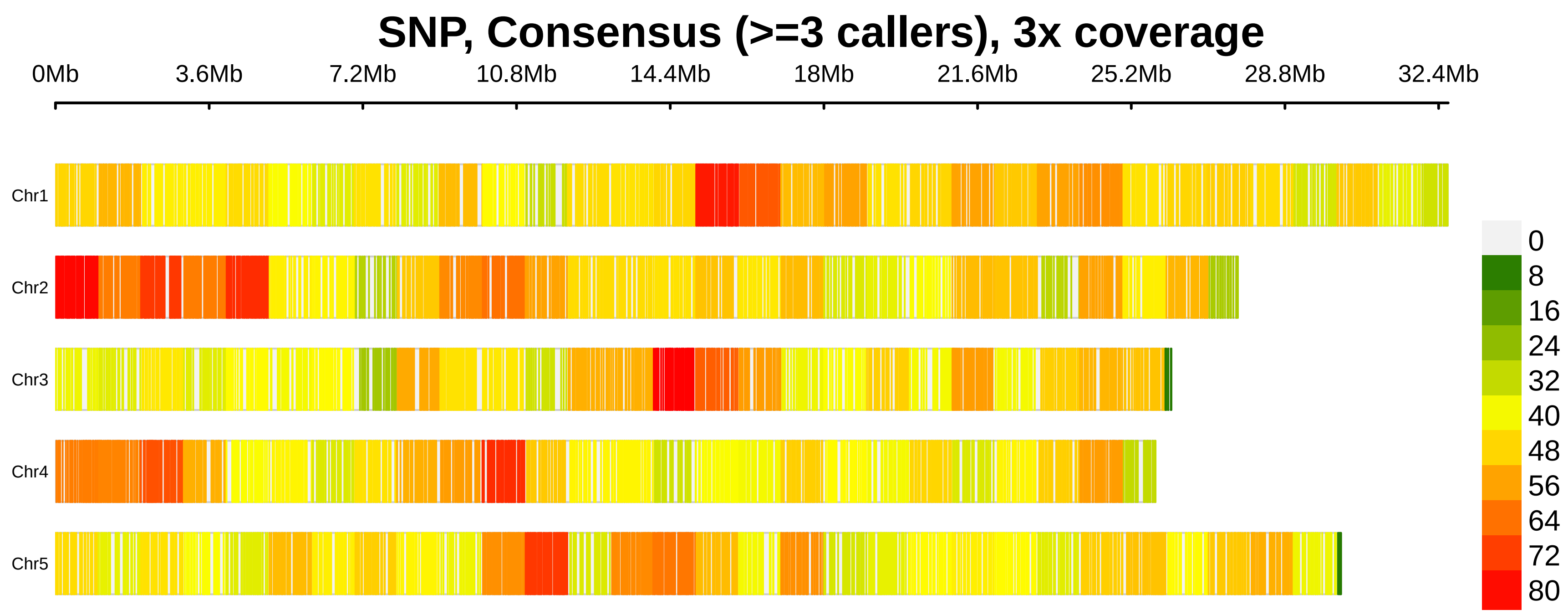
SNP, Consensus (>=3 callers), 3x coverage
0Mb
3.6Mb
7.2Mb
10.8Mb
14.4Mb
18Mb
21.6Mb
25.2Mb
28.8Mb
32.4Mb
Chr1
Chr2
Chr3
Chr4
Chr5
0
8
16
24
32
40
48
56
64
72
80

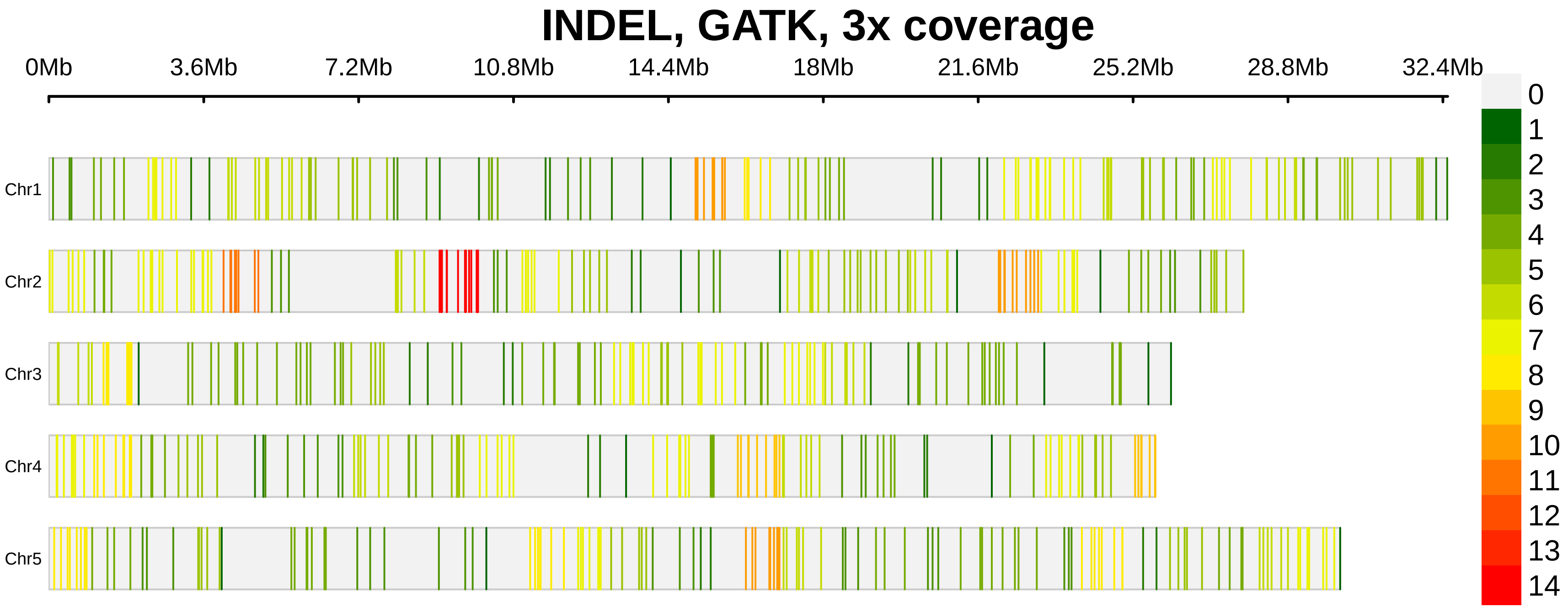
INDEL, GATK, 3x coverage
0Mb
3.6Mb
7.2Mb
10.8Mb
14.4Mb
18Mb
21.6Mb
25.2Mb
28.8Mb
32.4Mb
Chr1
Chr2
Chr3
Chr4
Chr5
0
1
2
3
4
5
6
7
8
9
10
11
12
13
14

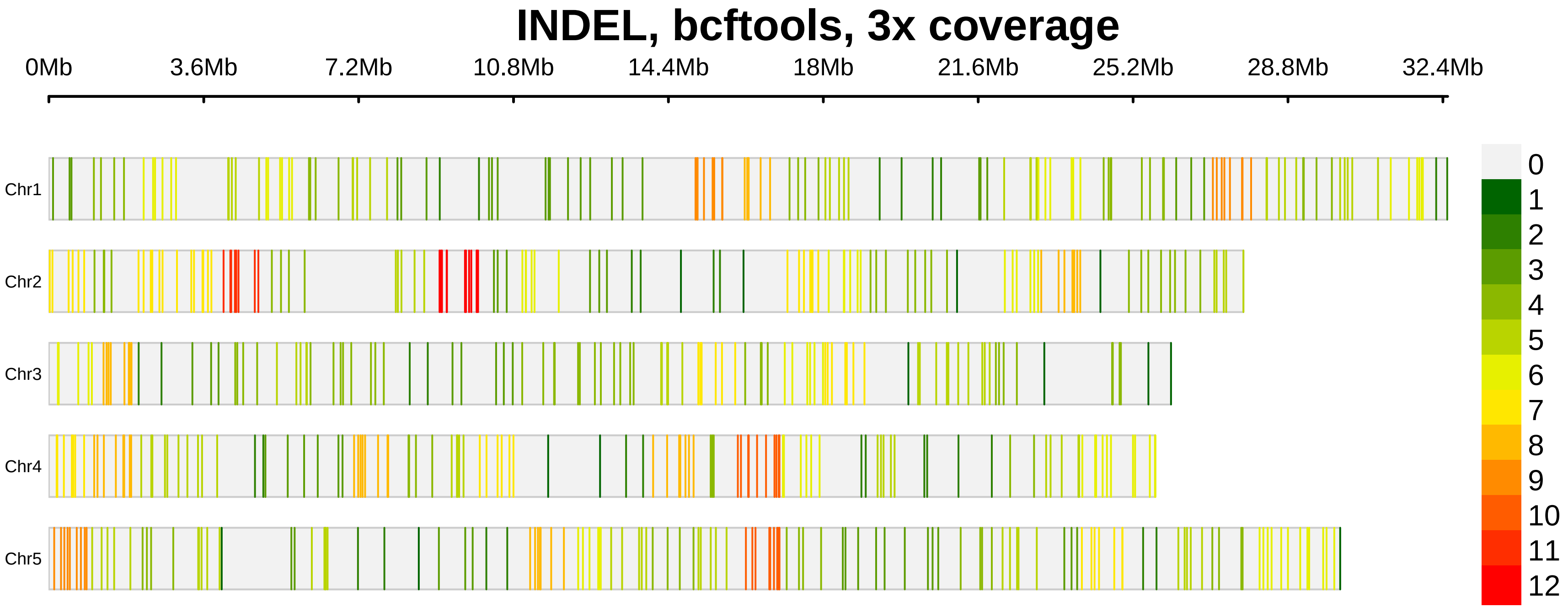

INDEL, bcftools, 3x coverage
0Mb
3.6Mb
7.2Mb
10.8Mb
14.4Mb
18Mb
21.6Mb
25.2Mb
28.8Mb
32.4Mb
Chr1
Chr2
Chr3
Chr4
Chr5
0
1
2
3
4
5
6
7
8
9
10
11
12

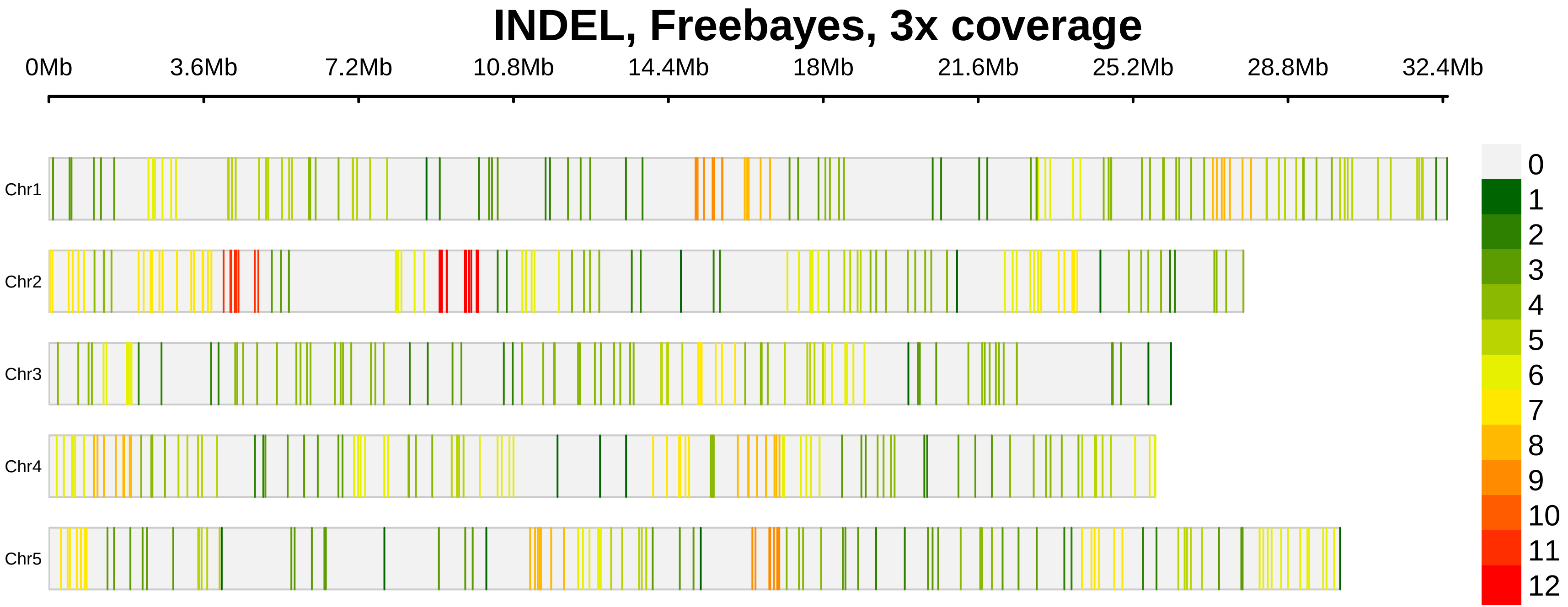
INDEL, Freebayes, 3x coverage
0Mb
3.6Mb
7.2Mb
10.8Mb
14.4Mb
18Mb
21.6Mb
25.2Mb
28.8Mb
32.4Mb
Chr1
Chr2
Chr3
Chr4
Chr5
0
1
2
3
4
5
6
7
8
9
10
11
12

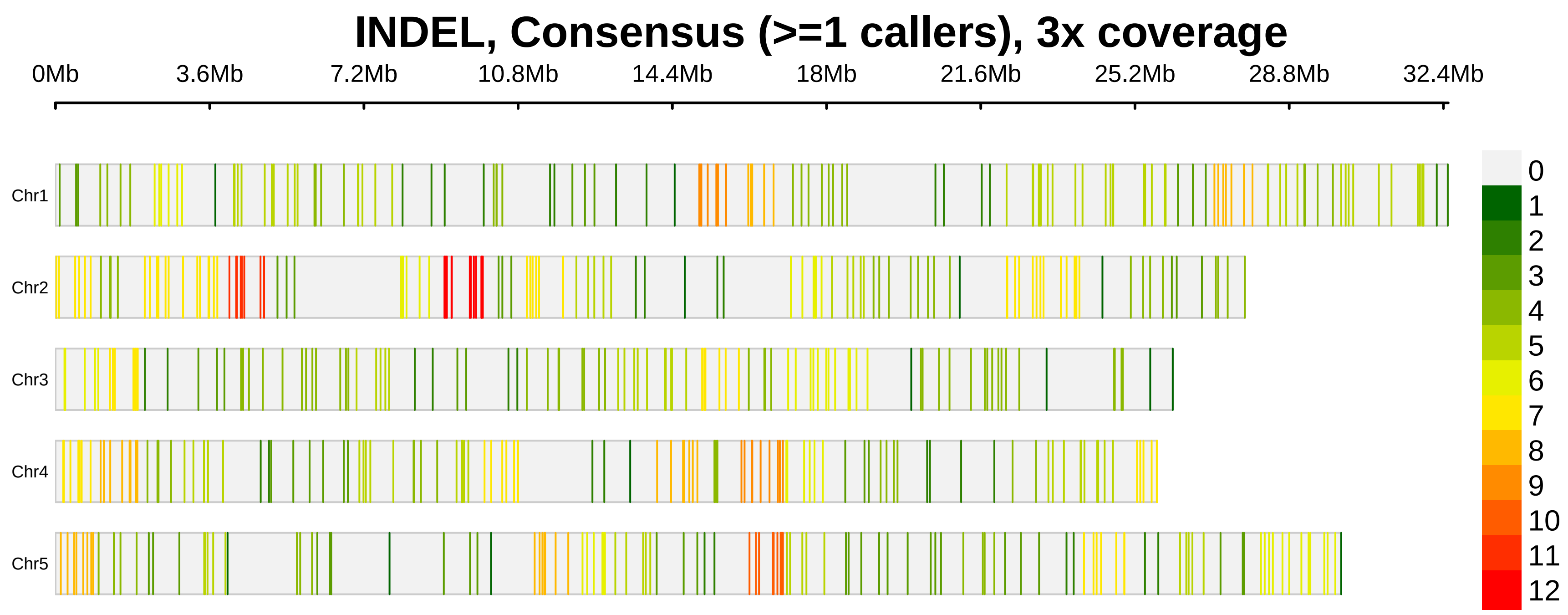
INDEL, Consensus (>=1 callers), 3x coverage
0Mb
3.6Mb
7.2Mb
10.8Mb
14.4Mb
18Mb
21.6Mb
25.2Mb
28.8Mb
32.4Mb
Chr1
Chr2
Chr3
Chr4
Chr5
0
1
2
3
4
5
6
7
8
9
10
11
12

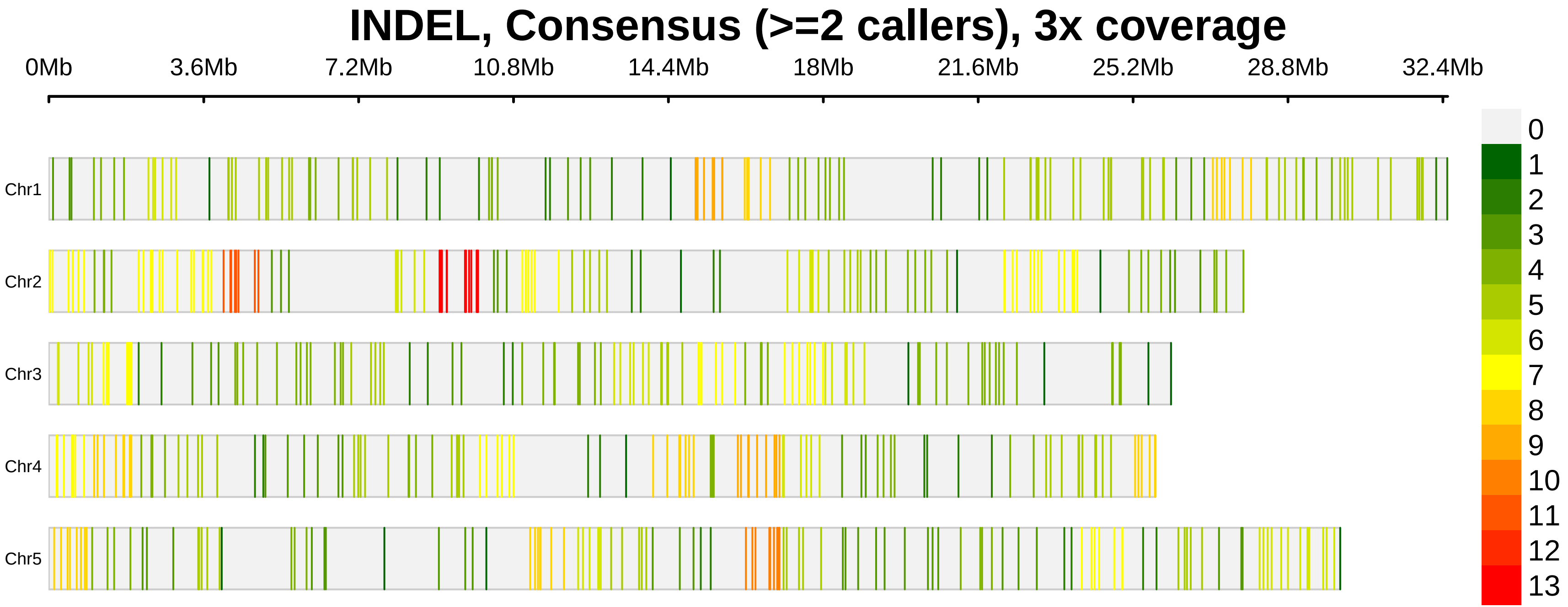
INDEL, Consensus (>=2 callers), 3x coverage
0Mb
3.6Mb
7.2Mb
10.8Mb
14.4Mb
18Mb
21.6Mb
25.2Mb
28.8Mb
32.4Mb
Chr1
Chr2
Chr3
Chr4
Chr5
0
1
2
3
4
5
6
7
8
9
10
11
12
13

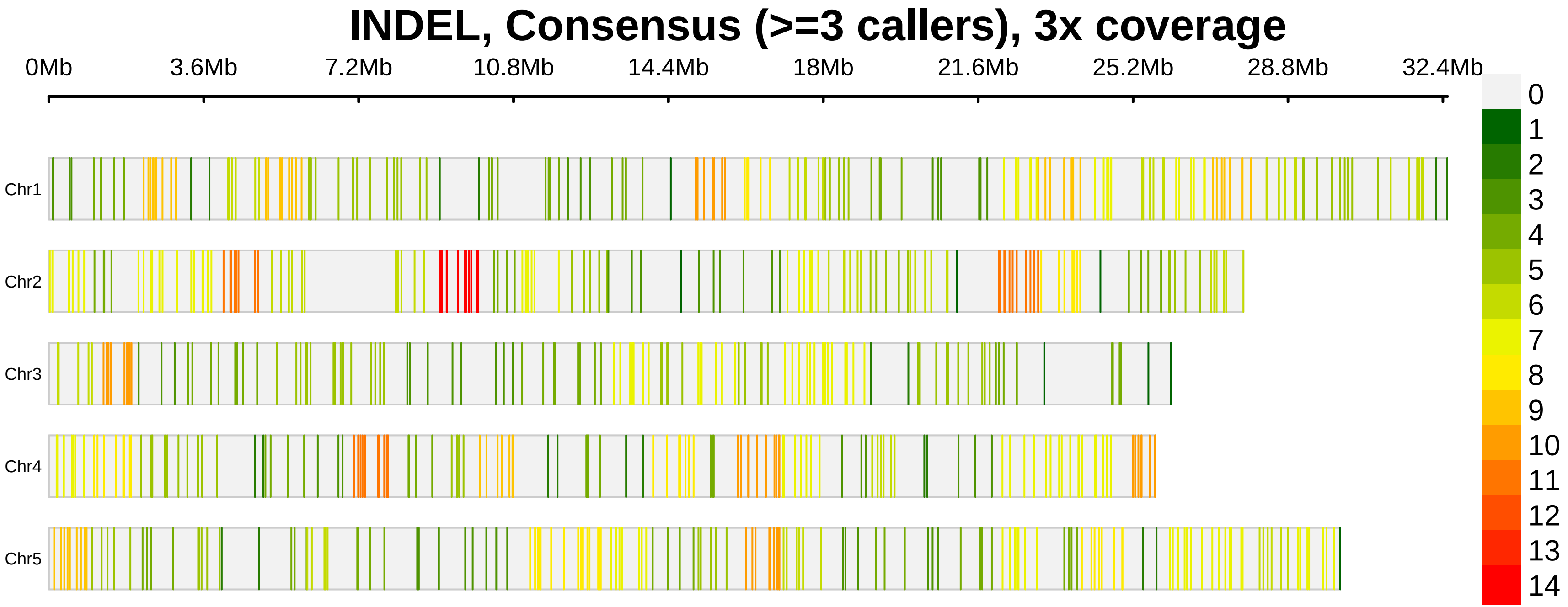
INDEL, Consensus (>=3 callers), 3x coverage
0Mb
3.6Mb
7.2Mb
10.8Mb
14.4Mb
18Mb
21.6Mb
25.2Mb
28.8Mb
32.4Mb
Chr1
Chr2
Chr3
Chr4
Chr5
0
1
2
3
4
5
6
7
8
9
10
11
12
13
14

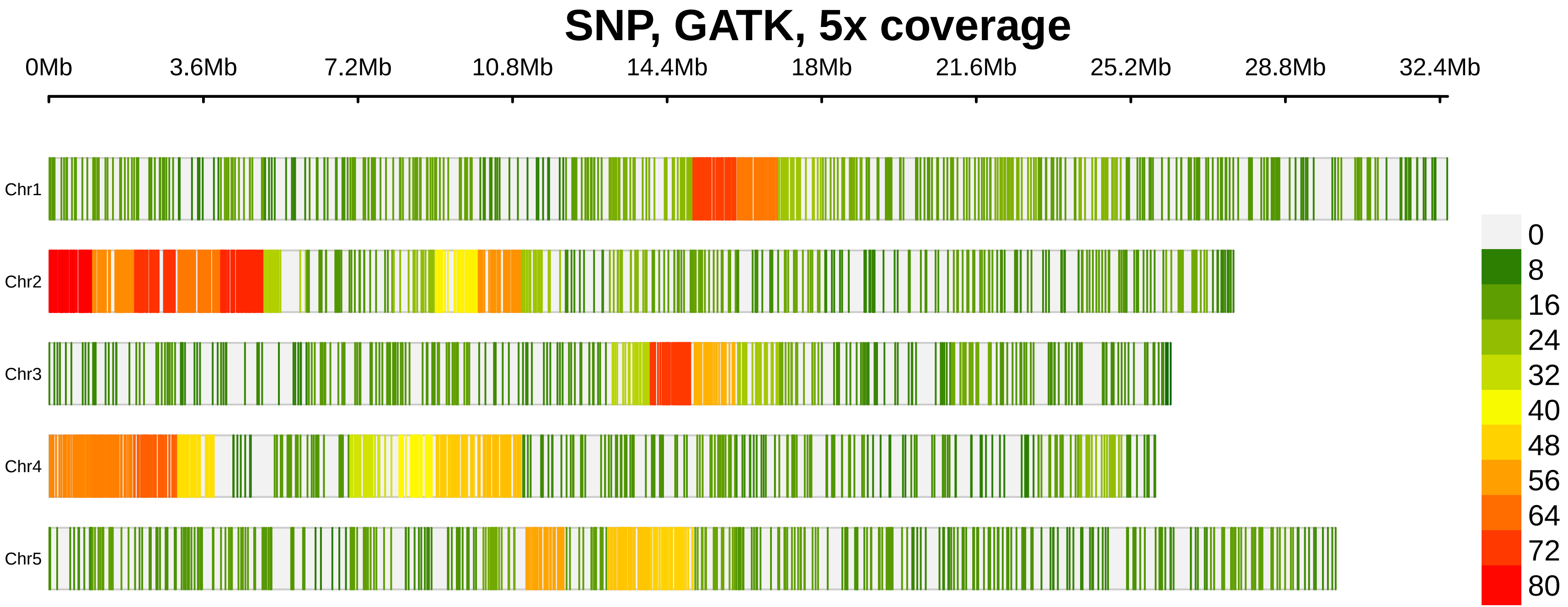

SNP, GATK, 5x coverage
0Mb
3.6Mb
7.2Mb
10.8Mb
14.4Mb
18Mb
21.6Mb
25.2Mb
28.8Mb
32.4Mb
Chr1
Chr2
Chr3
Chr4
Chr5
0
8
16
24
32
40
48
56
64
72
80

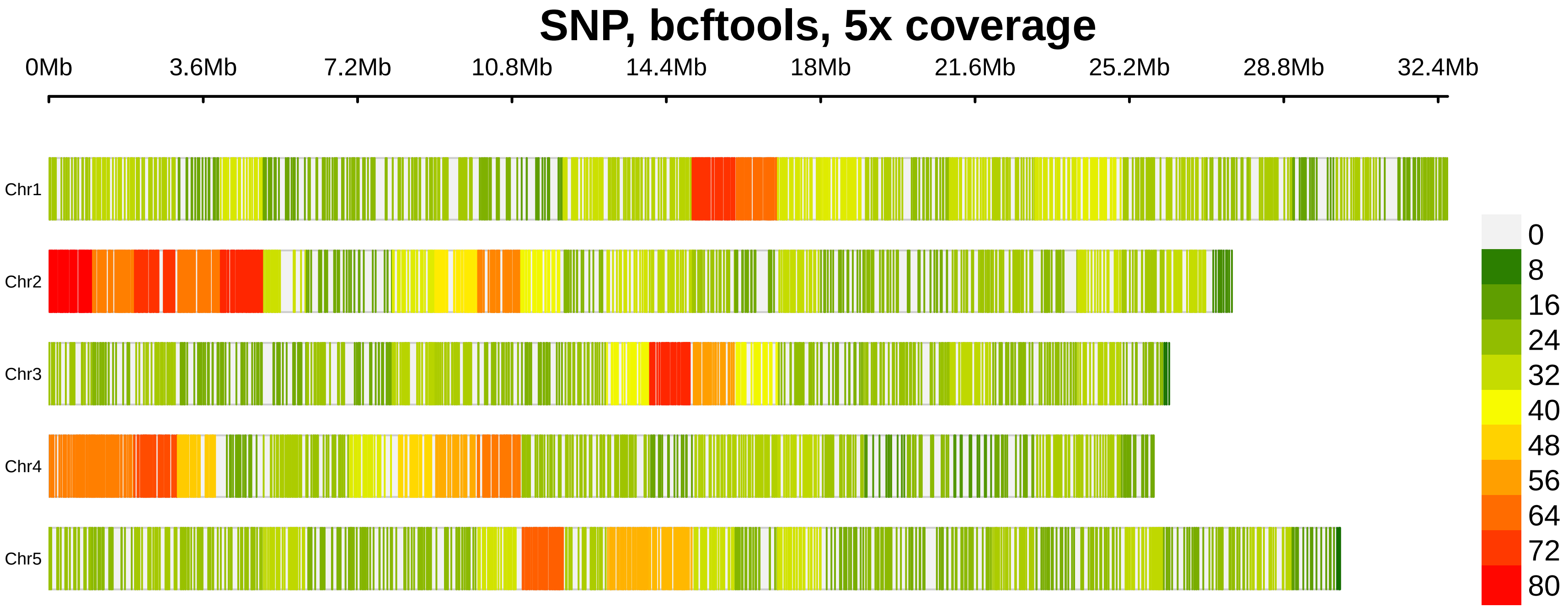
SNP, bcftools, 5x coverage
0Mb
3.6Mb
7.2Mb
10.8Mb
14.4Mb
18Mb
21.6Mb
25.2Mb
28.8Mb
32.4Mb
Chr1
Chr2
Chr3
Chr4
Chr5
0
8
16
24
32
40
48
56
64
72
80

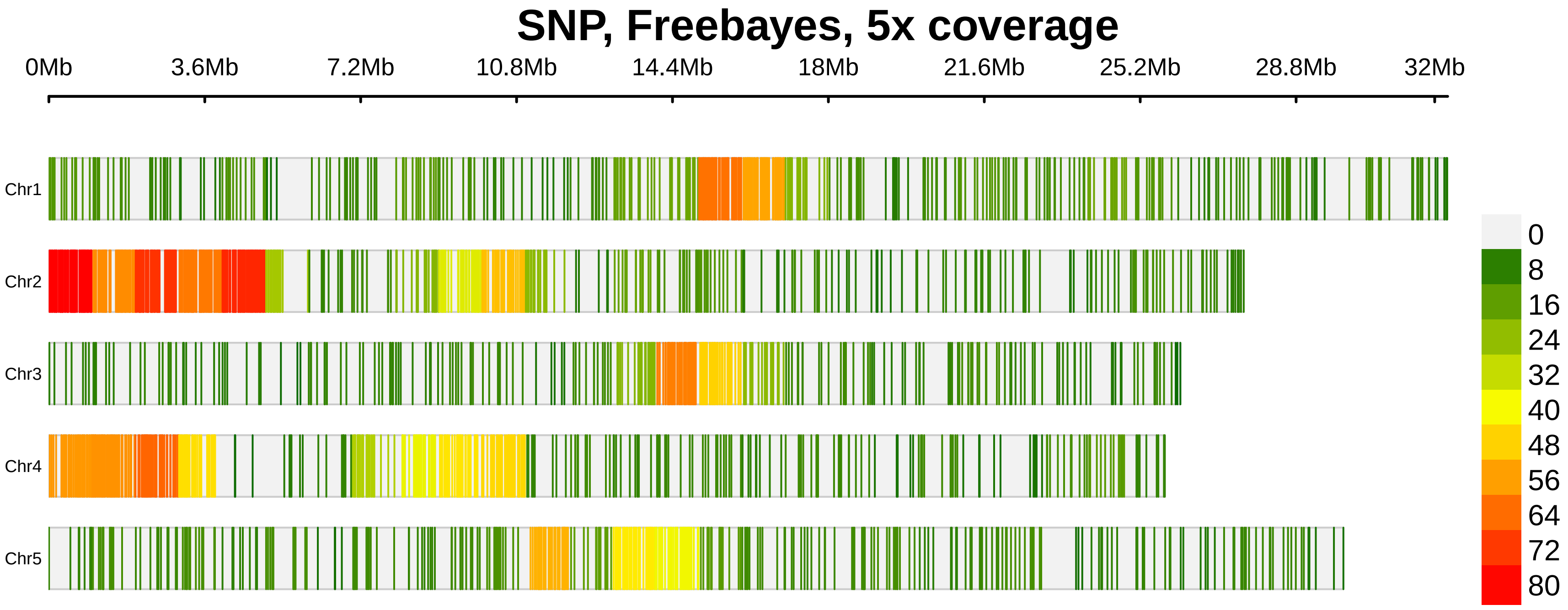
SNP, Freebayes, 5x coverage
0Mb
3.6Mb
7.2Mb
10.8Mb
14.4Mb
18Mb
21.6Mb
25.2Mb
28.8Mb
32Mb
Chr1
Chr2
Chr3
Chr4
Chr5
0
8
16
24
32
40
48
56
64
72
80

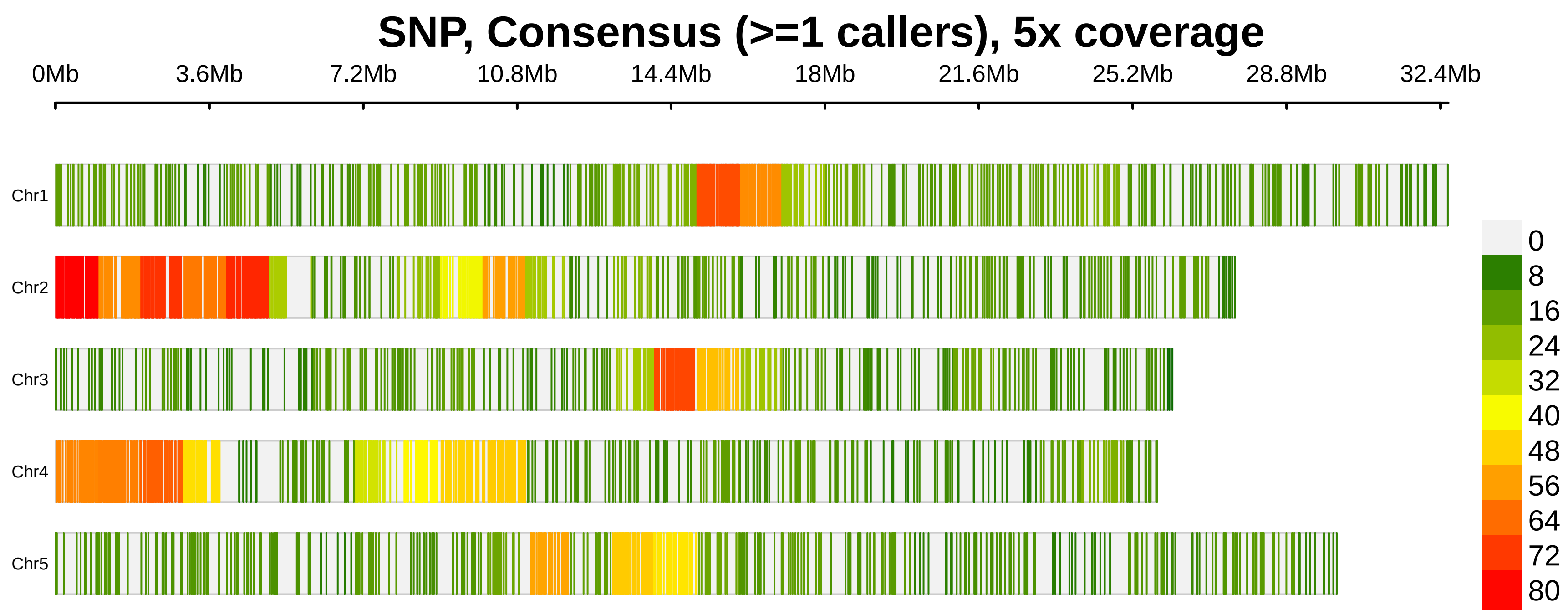
SNP, Consensus (>=1 callers), 5x coverage
0Mb
3.6Mb
7.2Mb
10.8Mb
14.4Mb
18Mb
21.6Mb
25.2Mb
28.8Mb
32.4Mb
Chr1
Chr2
Chr3
Chr4
Chr5
0
8
16
24
32
40
48
56
64
72
80

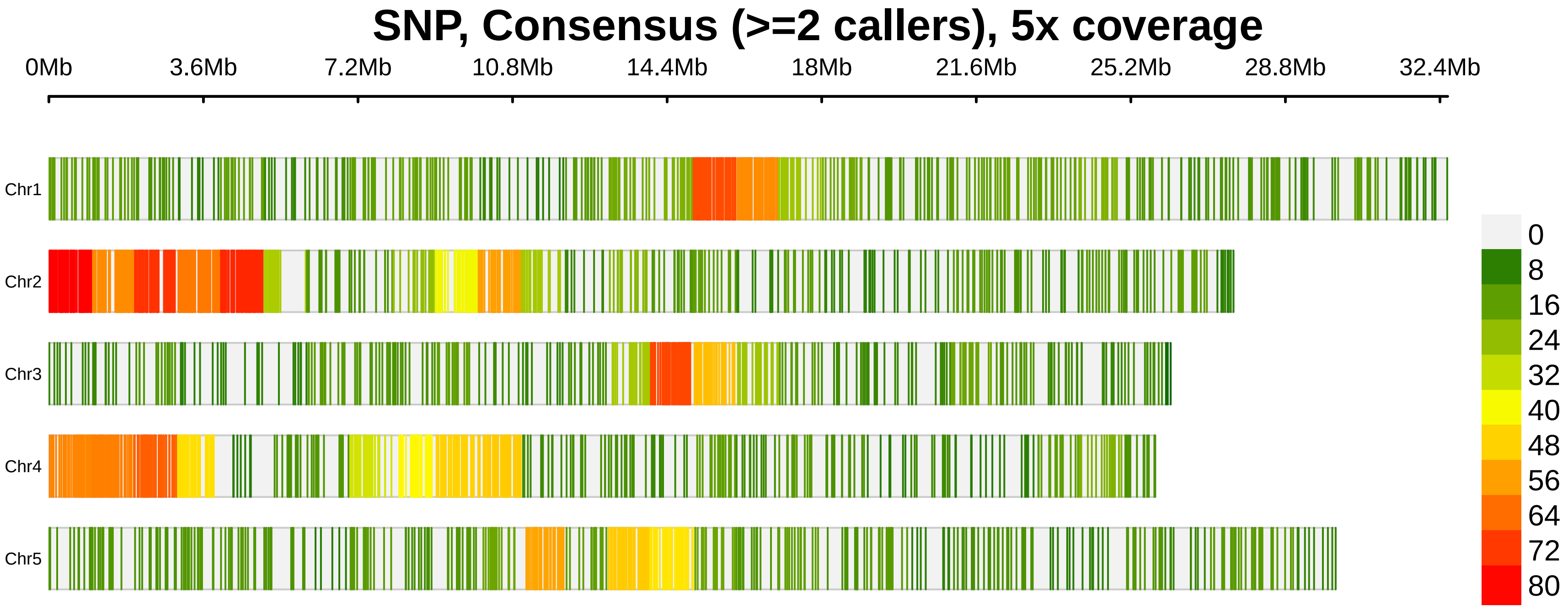

SNP, Consensus (>=2 callers), 5x coverage
0Mb
3.6Mb
7.2Mb
10.8Mb
14.4Mb
18Mb
21.6Mb
25.2Mb
28.8Mb
32.4Mb
Chr1
Chr2
Chr3
Chr4
Chr5
0
8
16
24
32
40
48
56
64
72
80

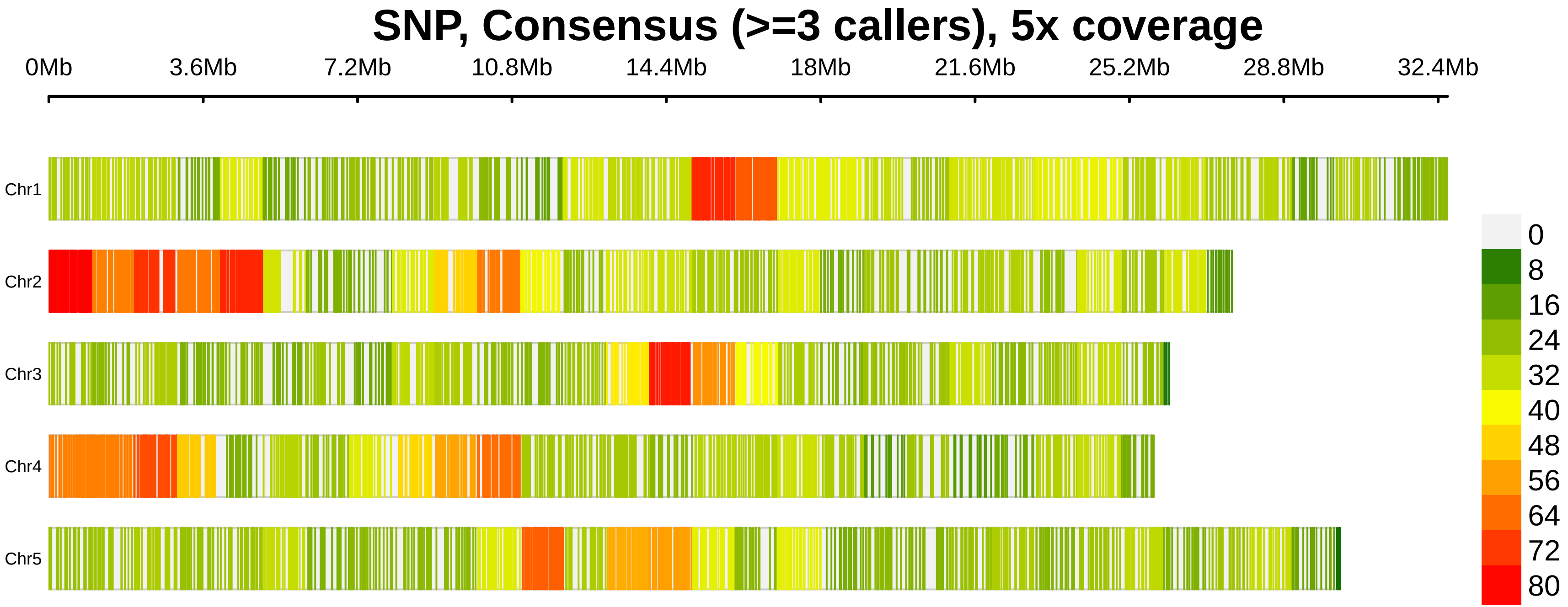
SNP, Consensus (>=3 callers), 5x coverage
0Mb
3.6Mb
7.2Mb
10.8Mb
14.4Mb
18Mb
21.6Mb
25.2Mb
28.8Mb
32.4Mb
Chr1
Chr2
Chr3
Chr4
Chr5
0
8
16
24
32
40
48
56
64
72
80

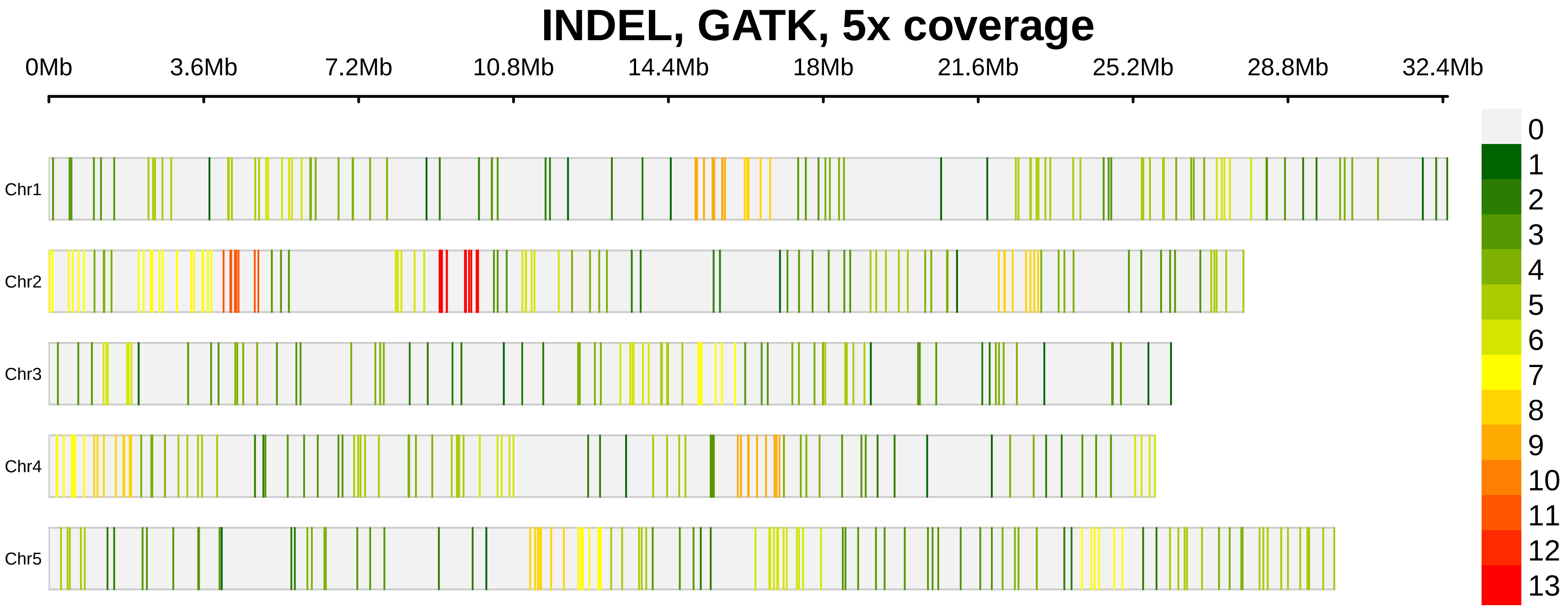
INDEL, GATK, 5x coverage
0Mb
3.6Mb
7.2Mb
10.8Mb
14.4Mb
18Mb
21.6Mb
25.2Mb
28.8Mb
32.4Mb
Chr1
Chr2
Chr3
Chr4
Chr5
0
1
2
3
4
5
6
7
8
9
10
11
12
13

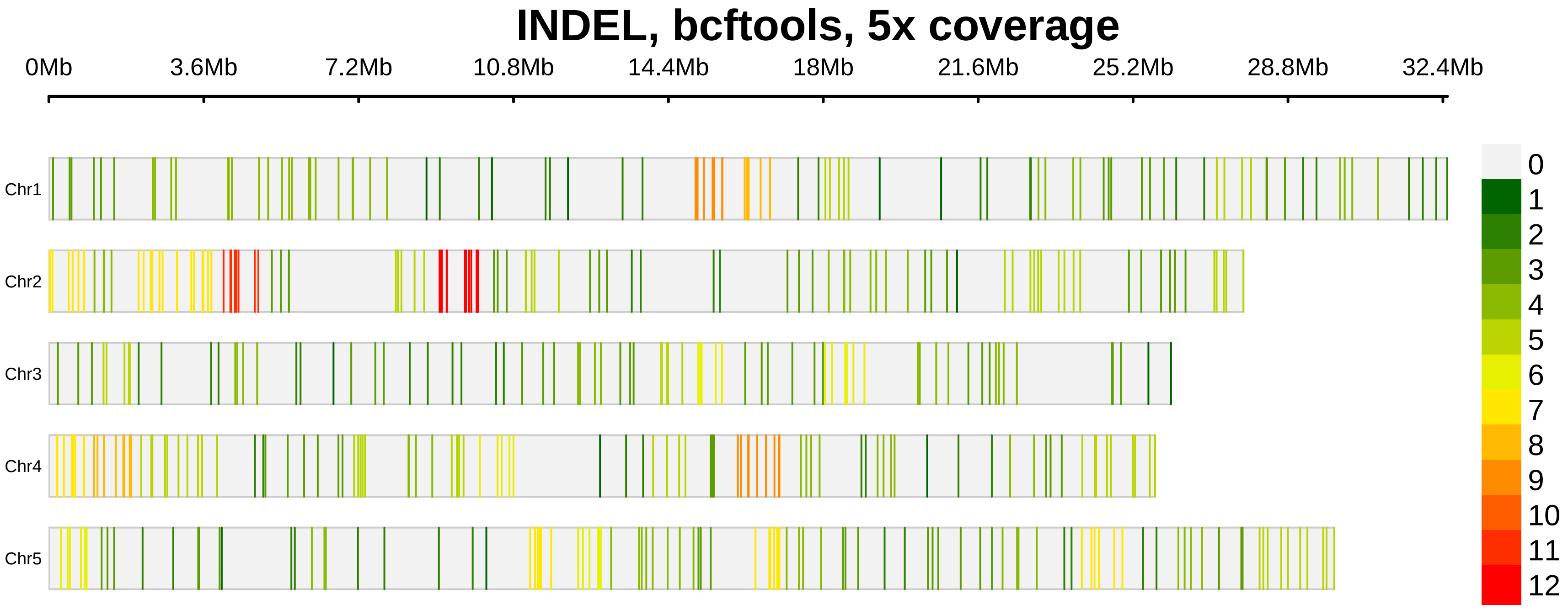

INDEL, bcftools, 5x coverage
0Mb
3.6Mb
7.2Mb
10.8Mb
14.4Mb
18Mb
21.6Mb
25.2Mb
28.8Mb
32.4Mb
Chr1
Chr2
Chr3
Chr4
Chr5
0
1
2
3
4
5
6
7
8
9
10
11
12

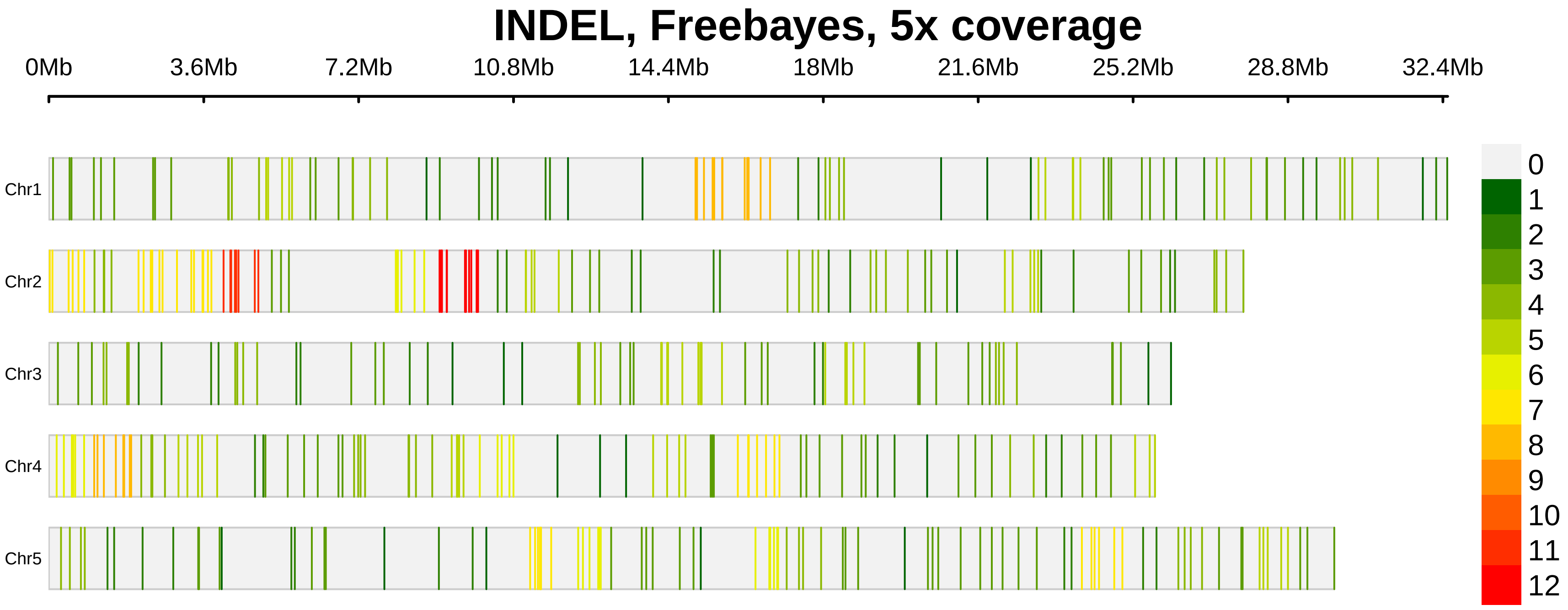
INDEL, Freebayes, 5x coverage
0Mb
3.6Mb
7.2Mb
10.8Mb
14.4Mb
18Mb
21.6Mb
25.2Mb
28.8Mb
32.4Mb
Chr1
Chr2
Chr3
Chr4
Chr5
0
1
2
3
4
5
6
7
8
9
10
11
12

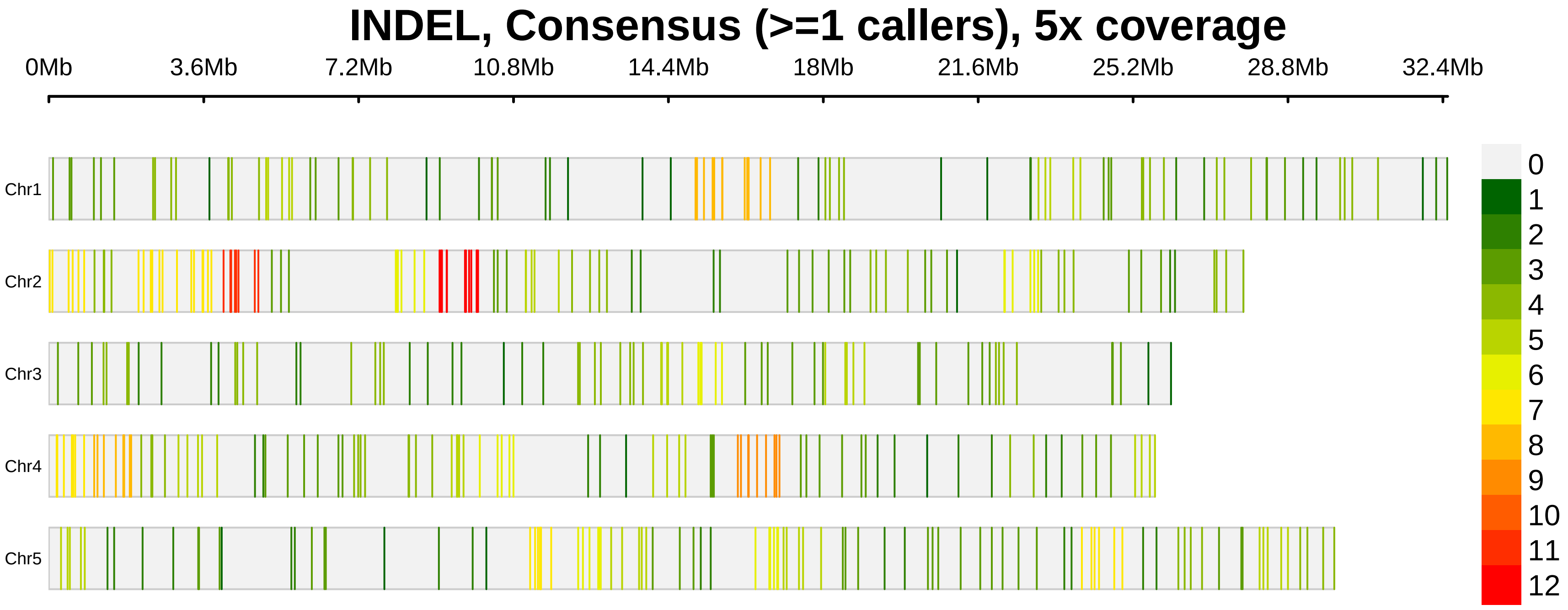
INDEL, Consensus (>=1 callers), 5x coverage
0Mb
3.6Mb
7.2Mb
10.8Mb
14.4Mb
18Mb
21.6Mb
25.2Mb
28.8Mb
32.4Mb
Chr1
Chr2
Chr3
Chr4
Chr5
0
1
2
3
4
5
6
7
8
9
10
11
12

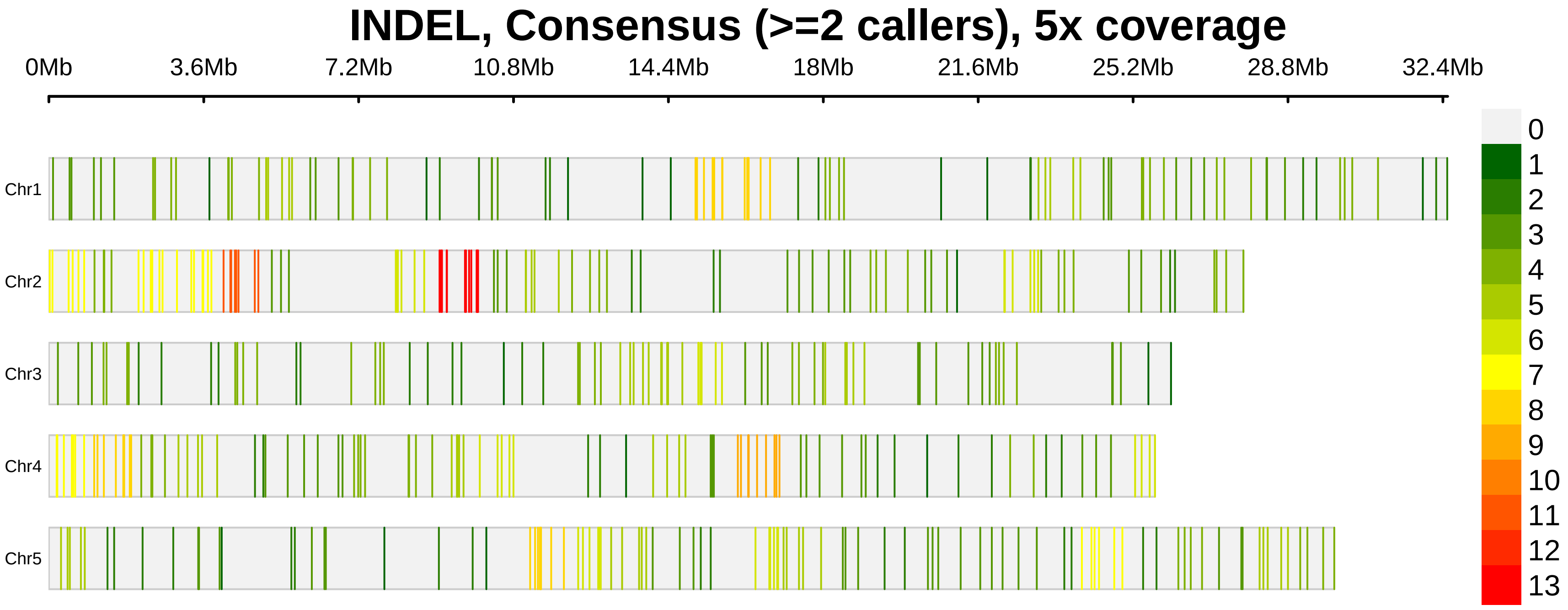

INDEL, Consensus (>=2 callers), 5x coverage
0Mb
3.6Mb
7.2Mb
10.8Mb
14.4Mb
18Mb
21.6Mb
25.2Mb
28.8Mb
32.4Mb
Chr1
Chr2
Chr3
Chr4
Chr5
0
1
2
3
4
5
6
7
8
9
10
11
12
13

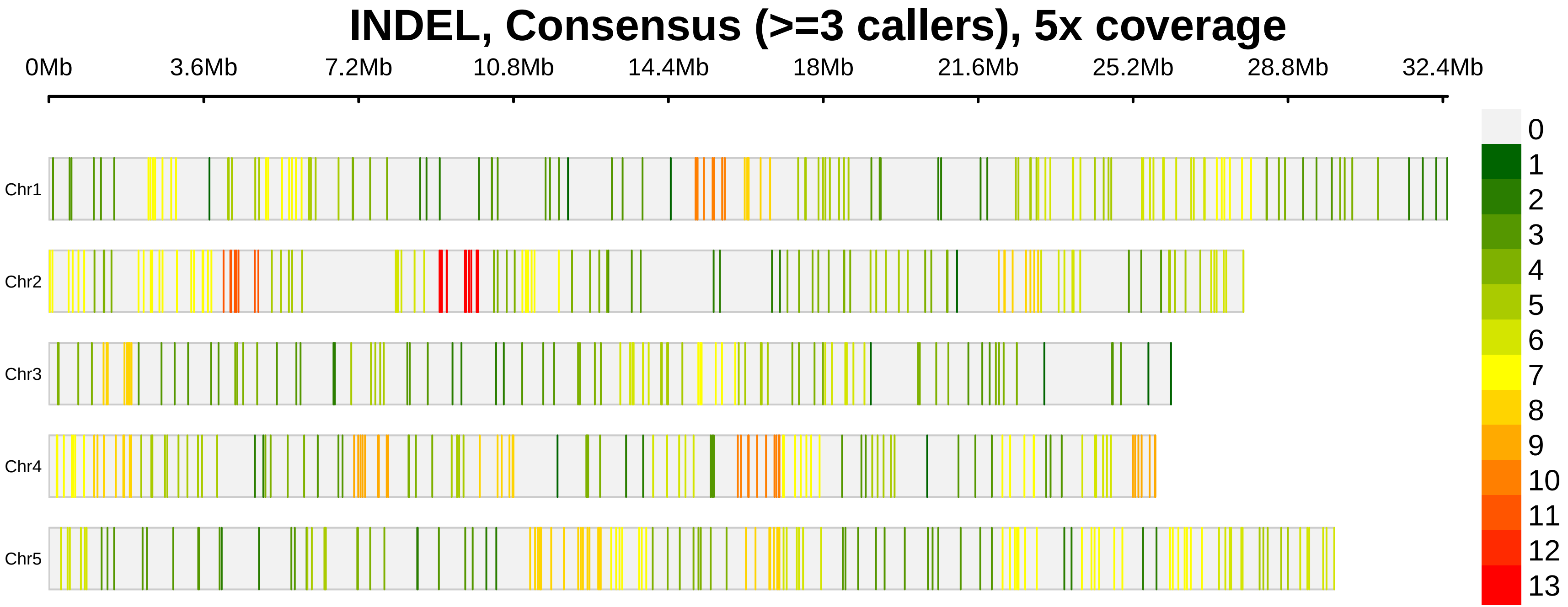
INDEL, Consensus (>=3 callers), 5x coverage
0Mb
3.6Mb
7.2Mb
10.8Mb
14.4Mb
18Mb
21.6Mb
25.2Mb
28.8Mb
32.4Mb
Chr1
Chr2
Chr3
Chr4
Chr5
0
1
2
3
4
5
6
7
8
9
10
11
12
13

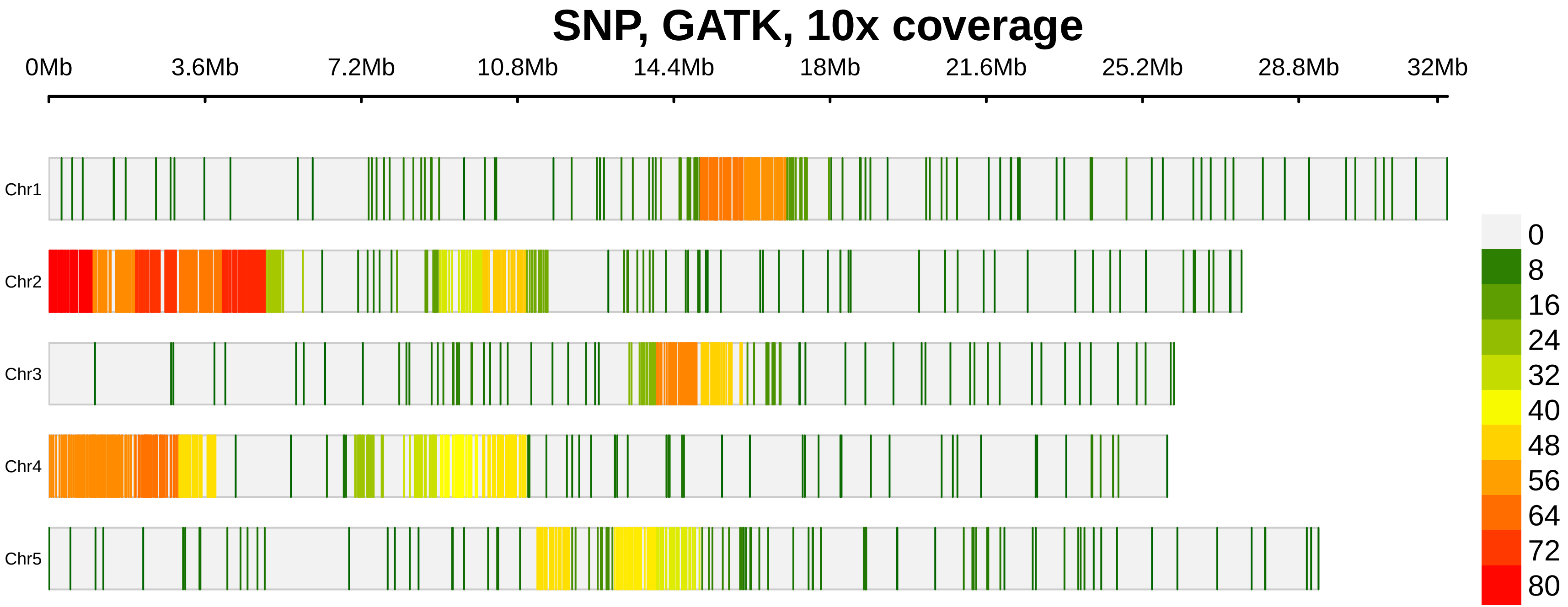

SNP, GATK, 10x coverage
0Mb
3.6Mb
7.2Mb
10.8Mb
14.4Mb
18Mb
21.6Mb
25.2Mb
28.8Mb
32Mb
Chr1
Chr2
Chr3
Chr4
Chr5
0
8
16
24
32
40
48
56
64
72
80

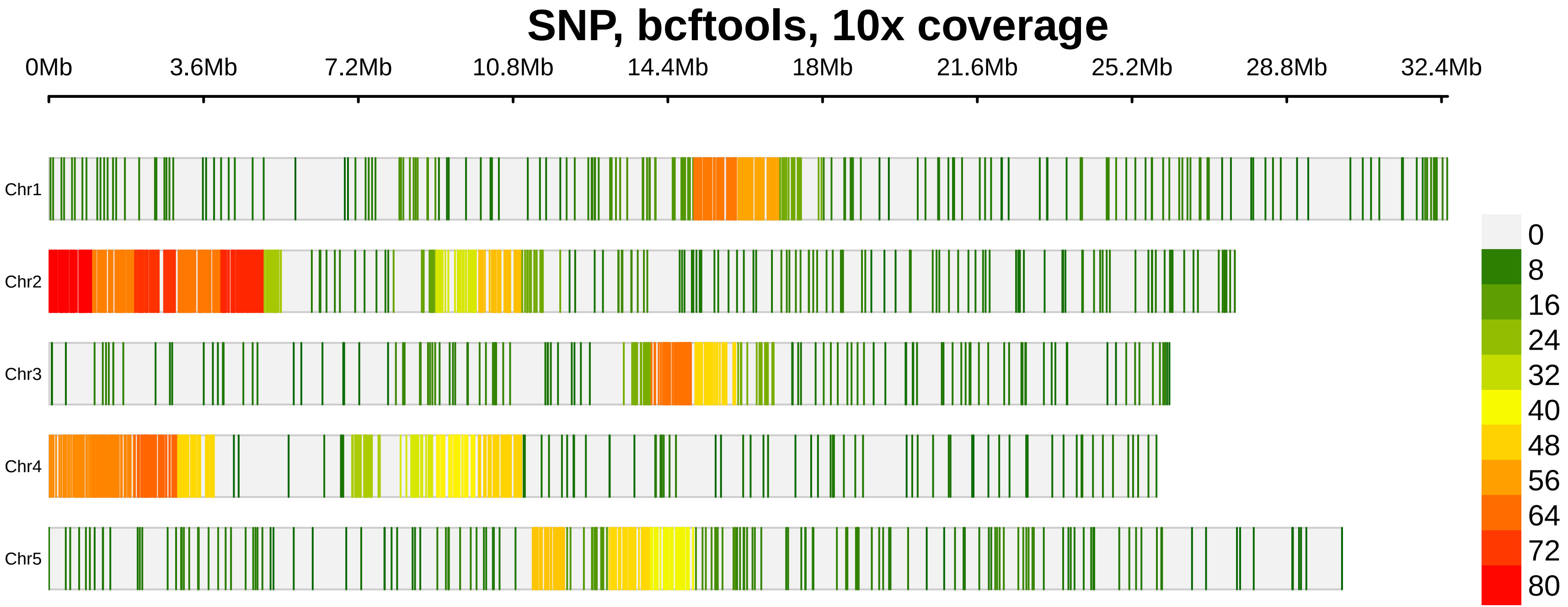
SNP, bcftools, 10x coverage
0Mb
3.6Mb
7.2Mb
10.8Mb
14.4Mb
18Mb
21.6Mb
25.2Mb
28.8Mb
32.4Mb
Chr1
Chr2
Chr3
Chr4
Chr5
0
8
16
24
32
40
48
56
64
72
80

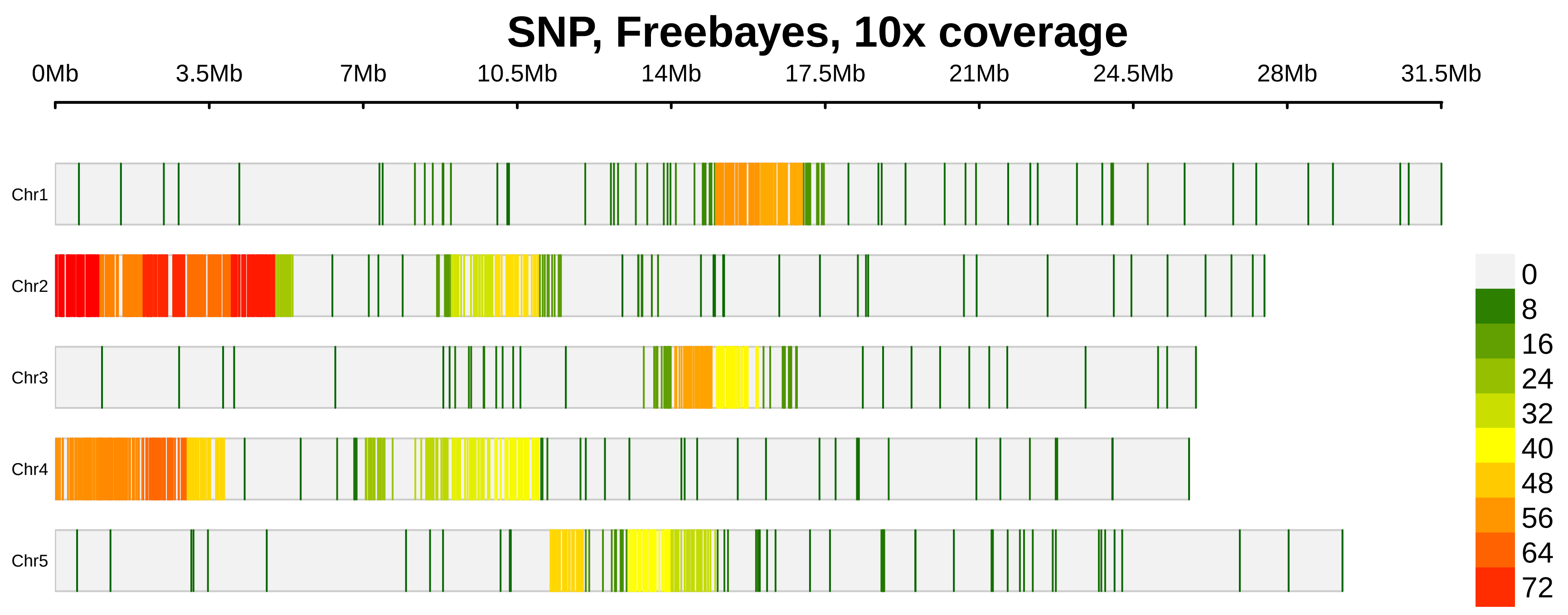
SNP, Freebayes, 10x coverage
0Mb
3.5Mb
7Mb
10.5Mb
14Mb
17.5Mb
21Mb
24.5Mb
28Mb
31.5Mb
Chr1
Chr2
Chr3
Chr4
Chr5
0
8
16
24
32
40
48
56
64
72

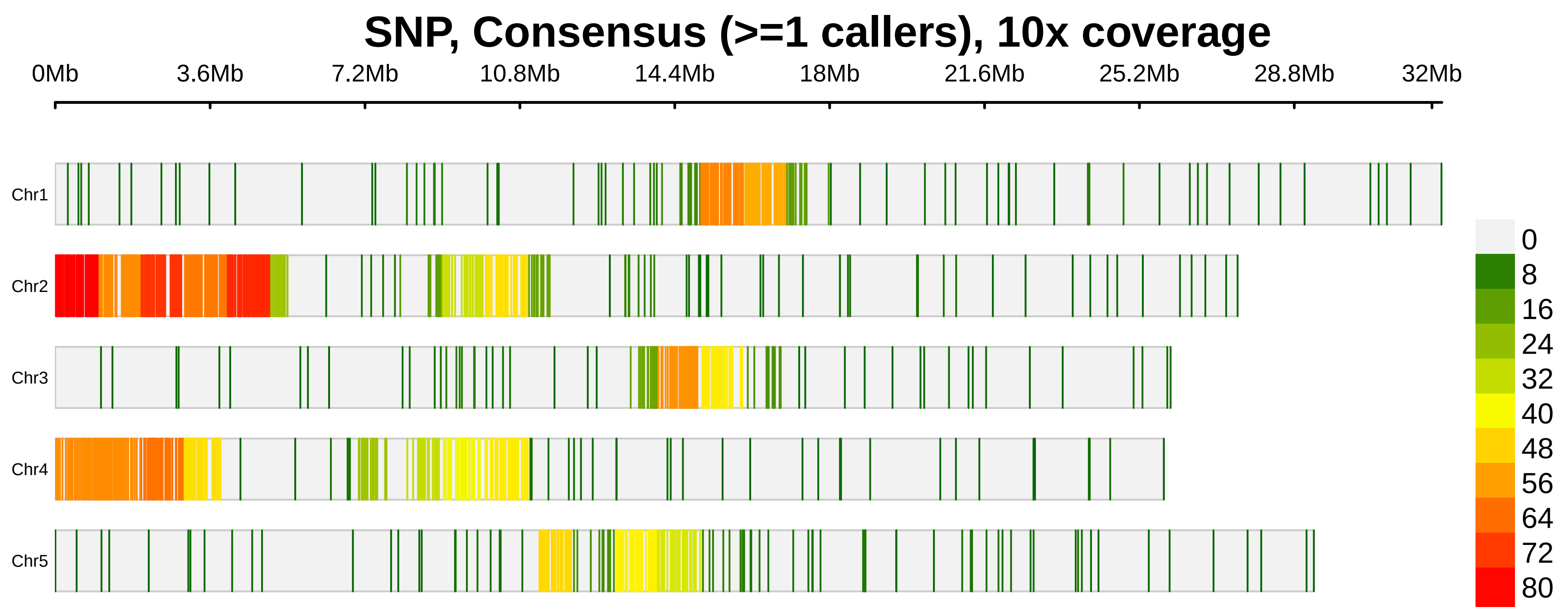
SNP, Consensus (>=1 callers), 10x coverage
0Mb
3.6Mb
7.2Mb
10.8Mb
14.4Mb
18Mb
21.6Mb
25.2Mb
28.8Mb
32Mb
Chr1
Chr2
Chr3
Chr4
Chr5
0
8
16
24
32
40
48
56
64
72
80

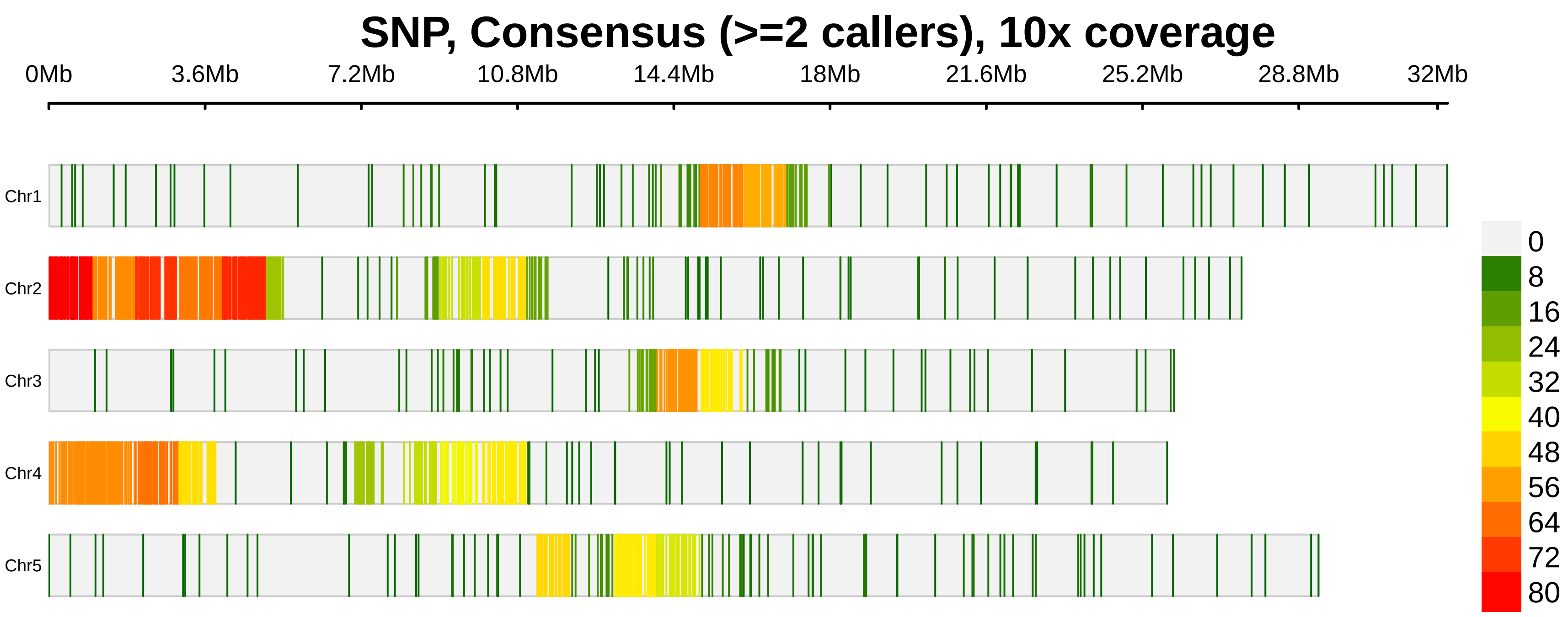
SNP, Consensus (>=2 callers), 10x coverage
0Mb
3.6Mb
7.2Mb
10.8Mb
14.4Mb
18Mb
21.6Mb
25.2Mb
28.8Mb
32Mb
Chr1
Chr2
Chr3
Chr4
Chr5
0
8
16
24
32
40
48
56
64
72
80

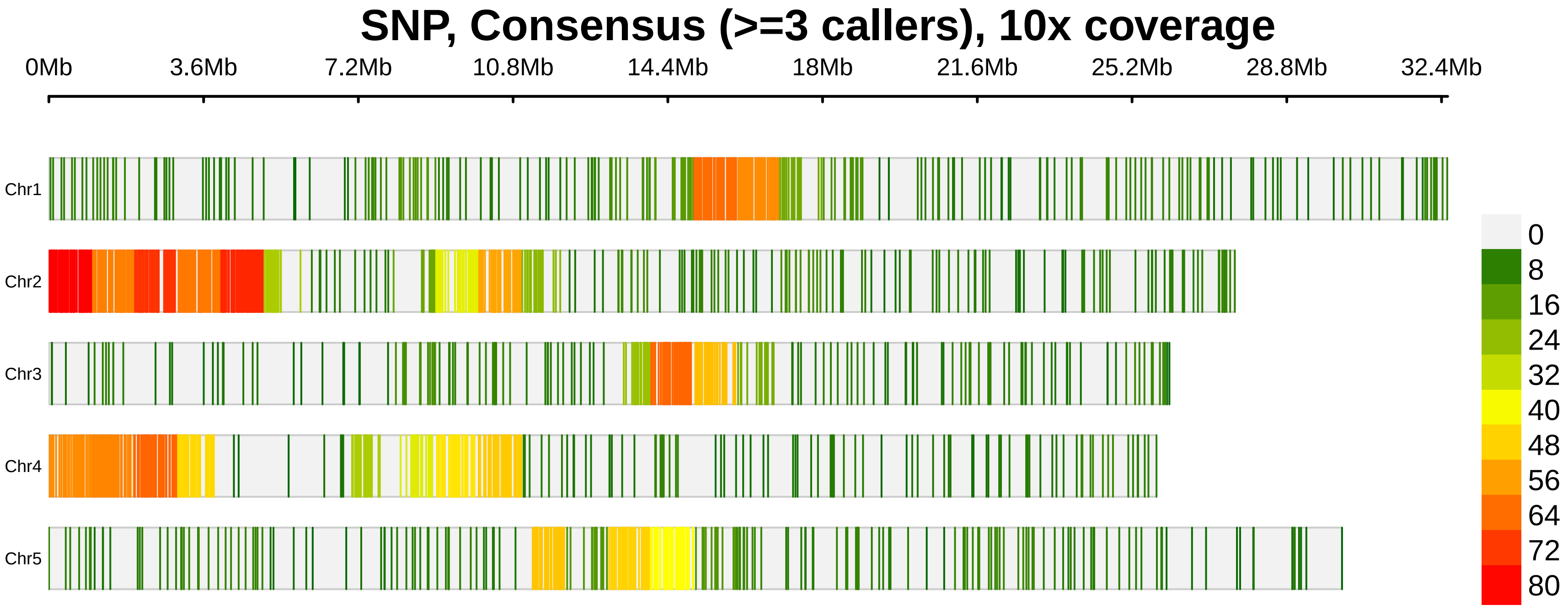
SNP, Consensus (>=3 callers), 10x coverage
0Mb
3.6Mb
7.2Mb
10.8Mb
14.4Mb
18Mb
21.6Mb
25.2Mb
28.8Mb
32.4Mb
Chr1
Chr2
Chr3
Chr4
Chr5
0
8
16
24
32
40
48
56
64
72
80

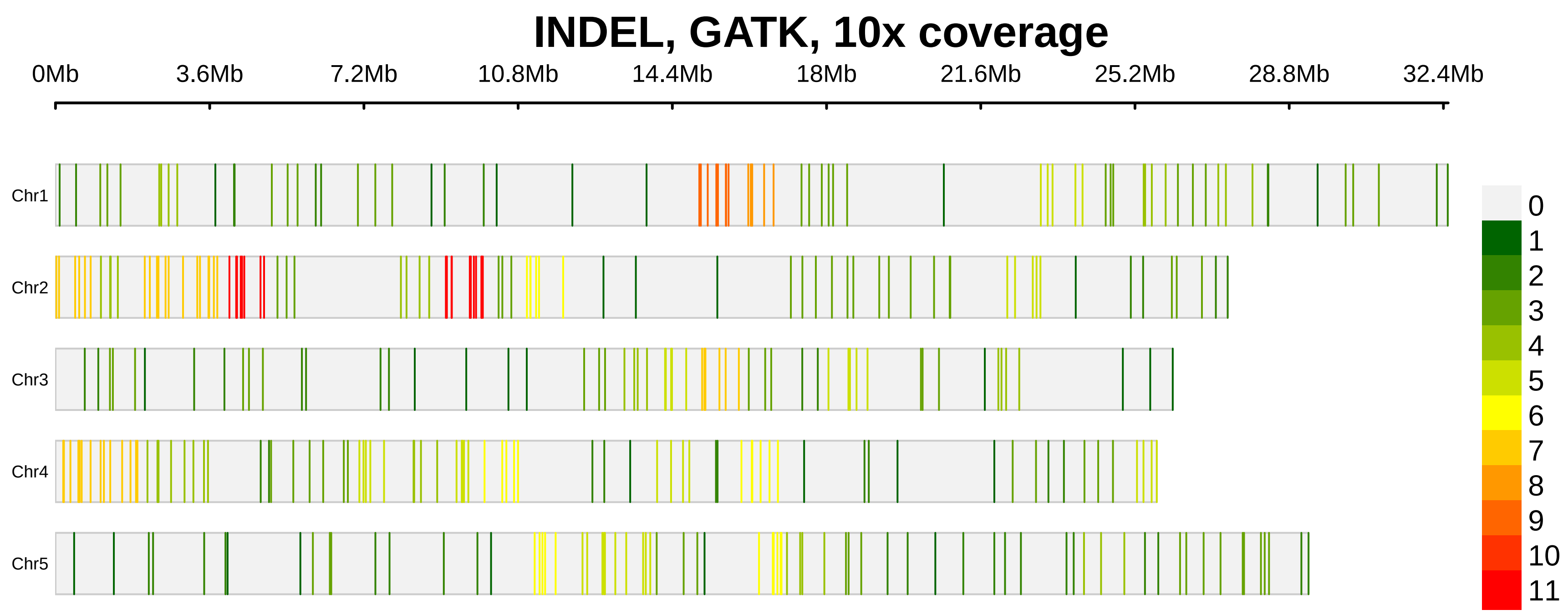
INDEL, GATK, 10x coverage
0Mb
3.6Mb
7.2Mb
10.8Mb
14.4Mb
18Mb
21.6Mb
25.2Mb
28.8Mb
32.4Mb
Chr1
Chr2
Chr3
Chr4
Chr5
0
1
2
3
4
5
6
7
8
9
10
11

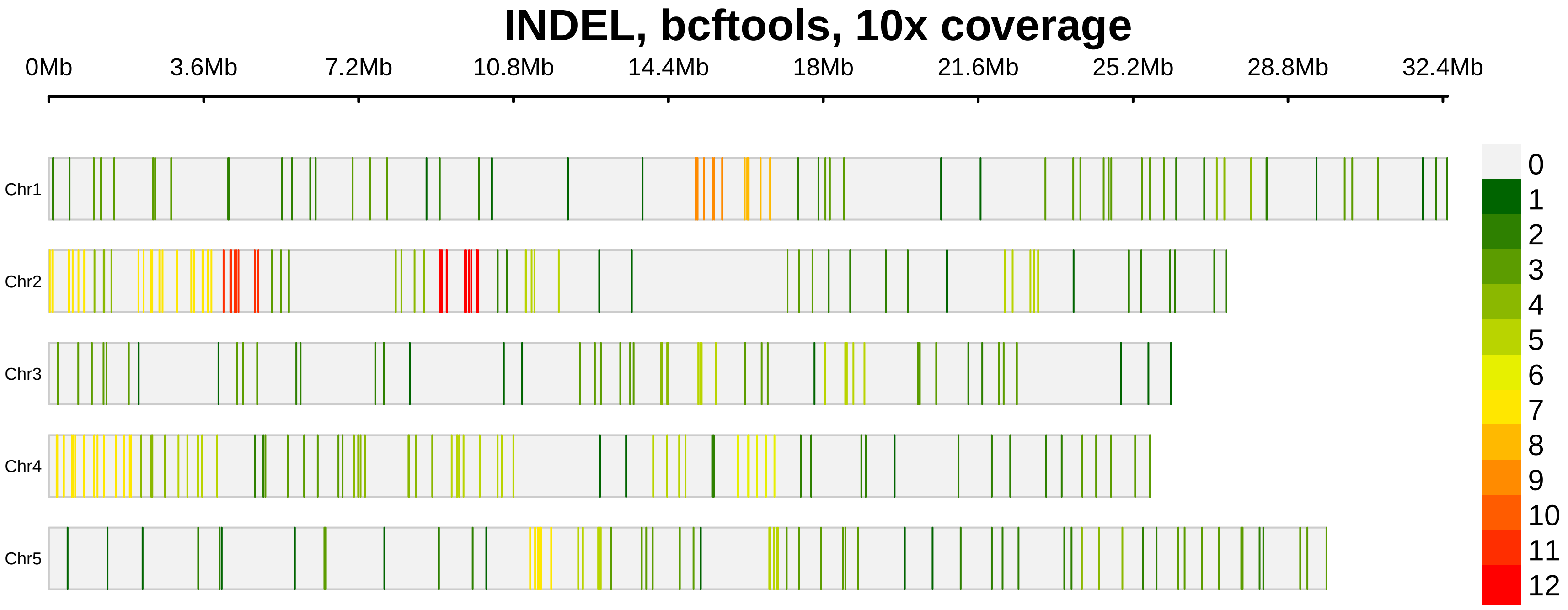
INDEL, bcftools, 10x coverage
0Mb
3.6Mb
7.2Mb
10.8Mb
14.4Mb
18Mb
21.6Mb
25.2Mb
28.8Mb
32.4Mb
Chr1
Chr2
Chr3
Chr4
Chr5
0
1
2
3
4
5
6
7
8
9
10
11
12

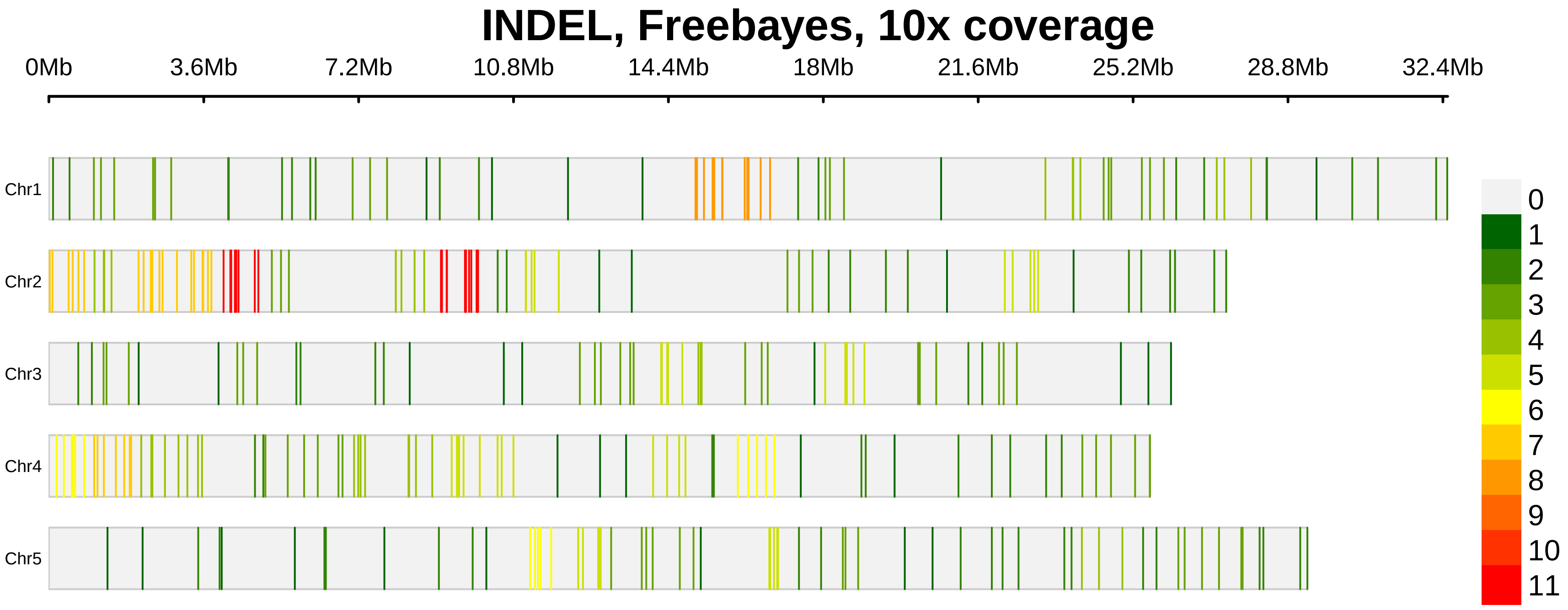
INDEL, Freebayes, 10x coverage
0Mb
3.6Mb
7.2Mb
10.8Mb
14.4Mb
18Mb
21.6Mb
25.2Mb
28.8Mb
32.4Mb
Chr1
Chr2
Chr3
Chr4
Chr5
0
1
2
3
4
5
6
7
8
9
10
11

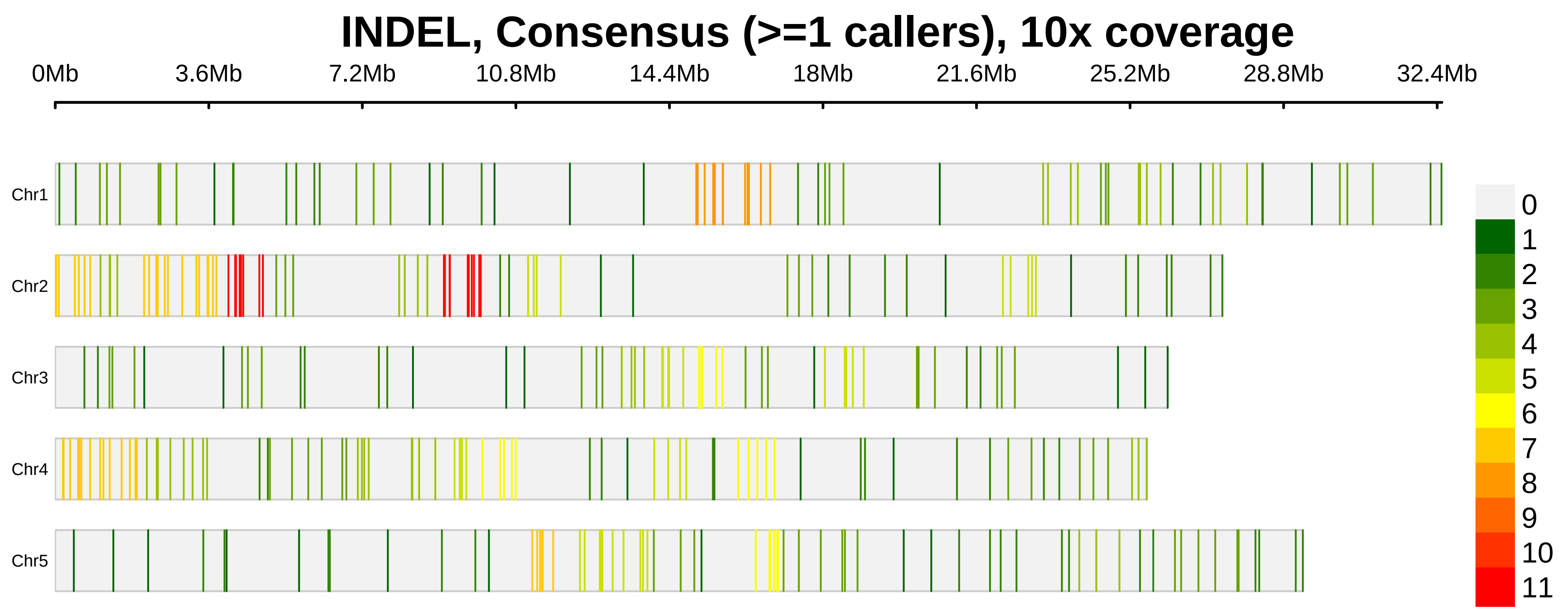

INDEL, Consensus (>=1 callers), 10x coverage
0Mb
3.6Mb
7.2Mb
10.8Mb
14.4Mb
18Mb
21.6Mb
25.2Mb
28.8Mb
32.4Mb
Chr1
Chr2
Chr3
Chr4
Chr5
0
1
2
3
4
5
6
7
8
9
10
11

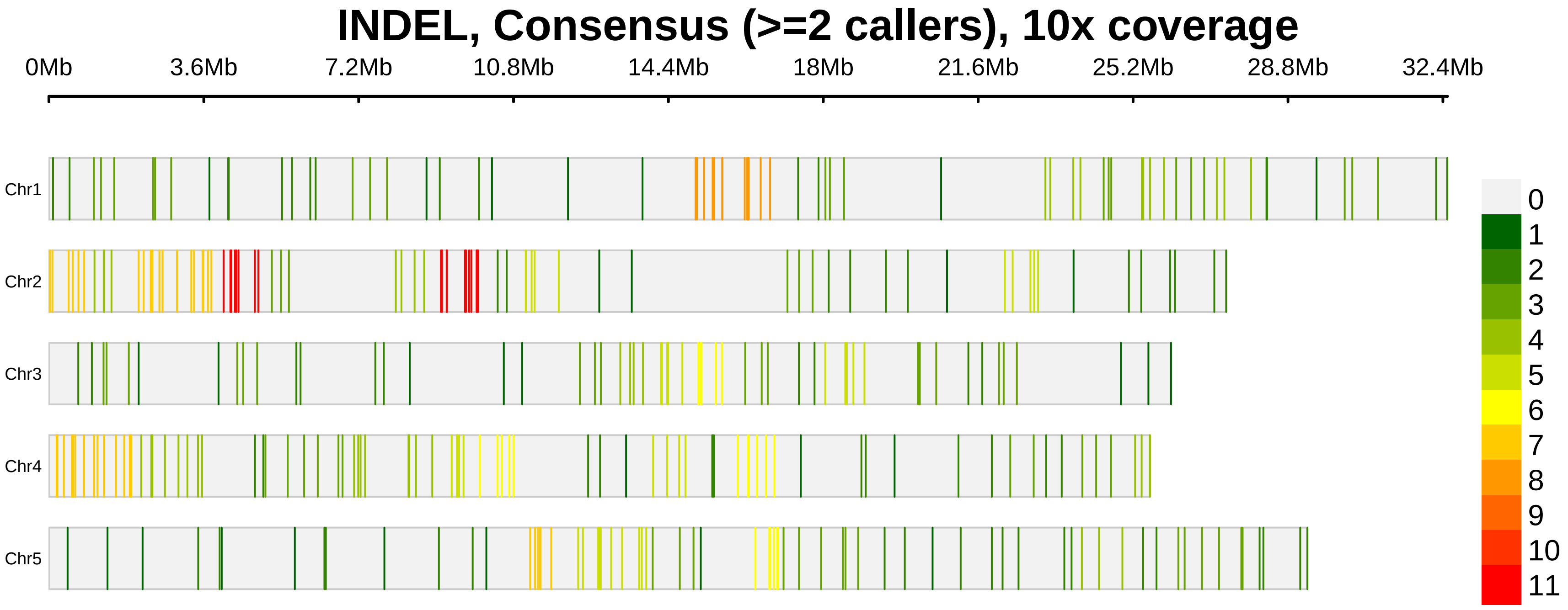

INDEL, Consensus (>=2 callers), 10x coverage
0Mb
3.6Mb
7.2Mb
10.8Mb
14.4Mb
18Mb
21.6Mb
25.2Mb
28.8Mb
32.4Mb
Chr1
Chr2
Chr3
Chr4
Chr5
0
1
2
3
4
5
6
7
8
9
10
11

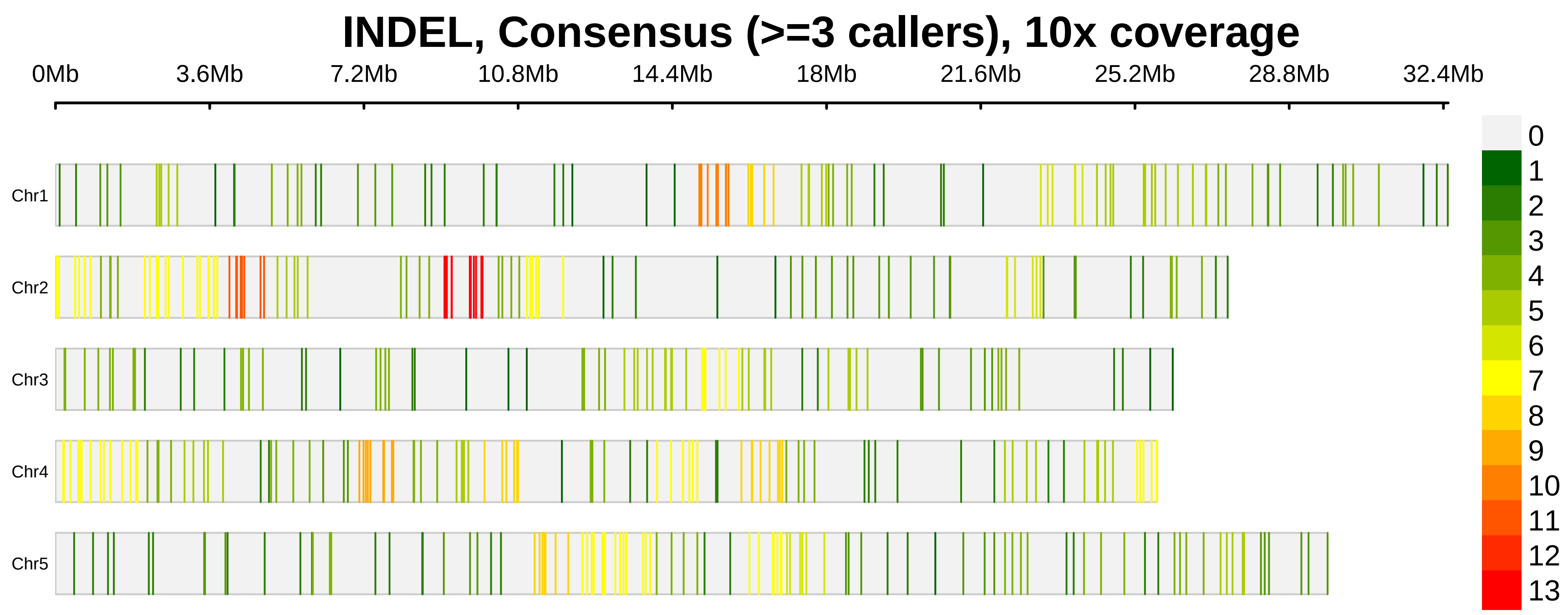
INDEL, Consensus (>=3 callers), 10x coverage
0Mb
3.6Mb
7.2Mb
10.8Mb
14.4Mb
18Mb
21.6Mb
25.2Mb
28.8Mb
32.4Mb
Chr1
Chr2
Chr3
Chr4
Chr5
0
1
2
3
4
5
6
7
8
9
10
11
12
13

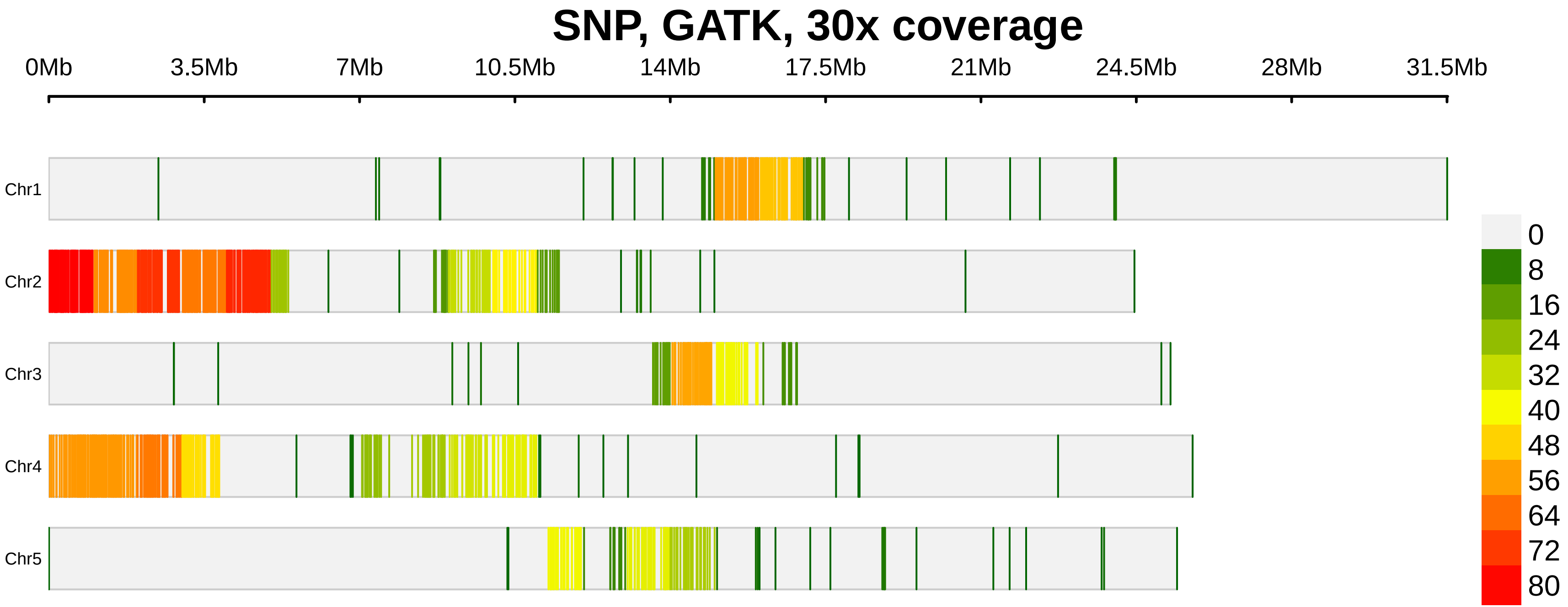
SNP, GATK, 30x coverage
0Mb
3.5Mb
7Mb
10.5Mb
14Mb
17.5Mb
21Mb
24.5Mb
28Mb
31.5Mb
Chr1
Chr2
Chr3
Chr4
Chr5
0
8
16
24
32
40
48
56
64
72
80

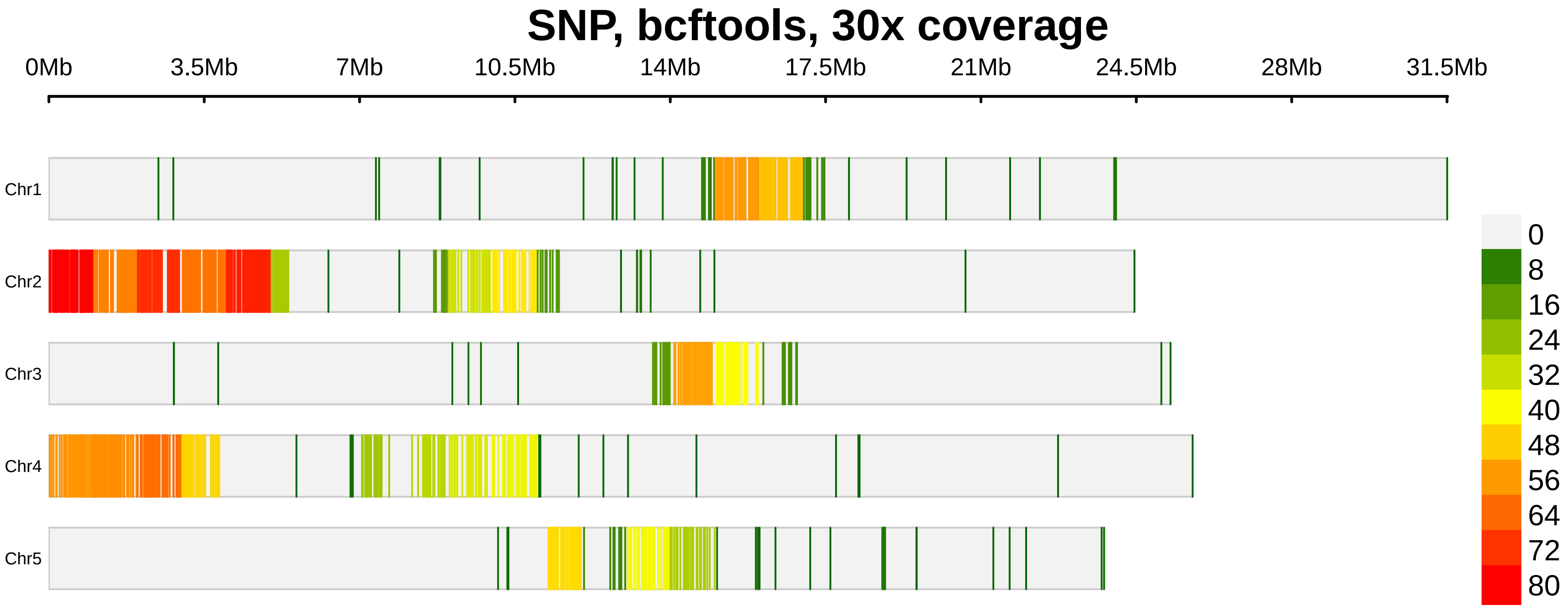

SNP, bcftools, 30x coverage
0Mb
3.5Mb
7Mb
10.5Mb
14Mb
17.5Mb
21Mb
24.5Mb
28Mb
31.5Mb
Chr1
Chr2
Chr3
Chr4
Chr5
0
8
16
24
32
40
48
56
64
72
80

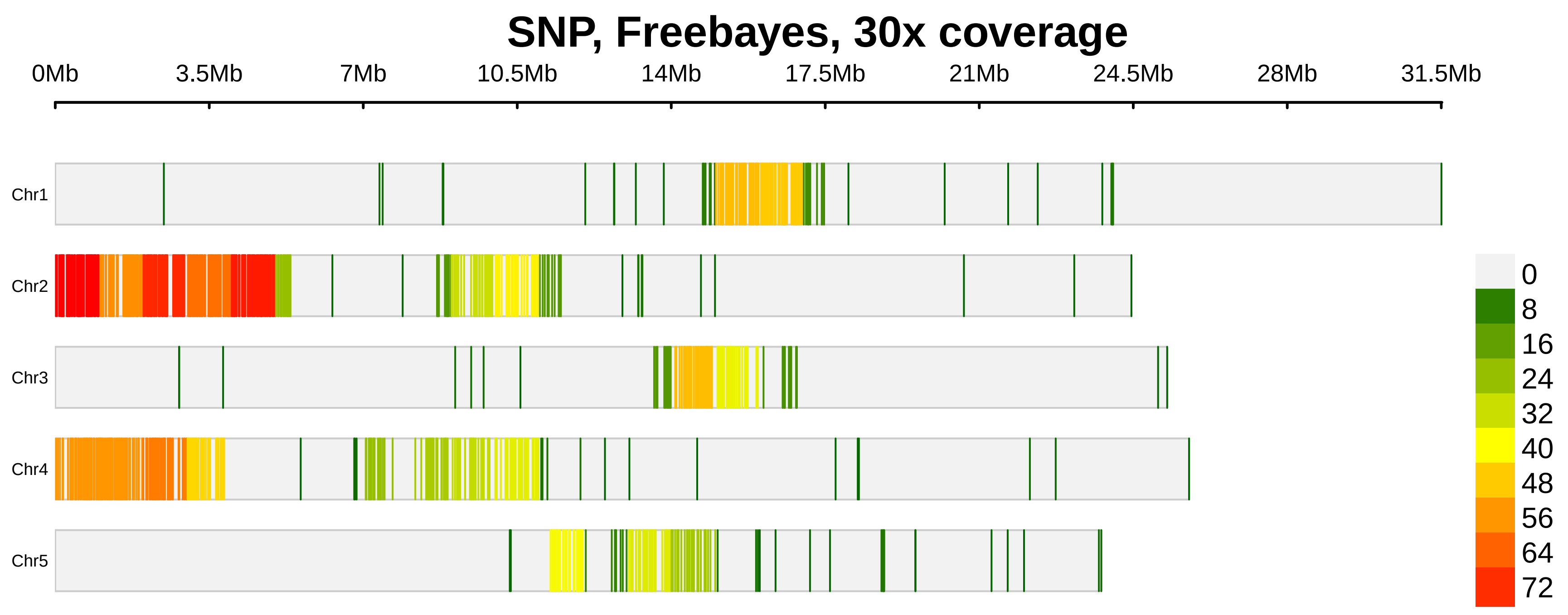
SNP, Freebayes, 30x coverage
0Mb
3.5Mb
7Mb
10.5Mb
14Mb
17.5Mb
21Mb
24.5Mb
28Mb
31.5Mb
Chr1
Chr2
Chr3
Chr4
Chr5
0
8
16
24
32
40
48
56
64
72

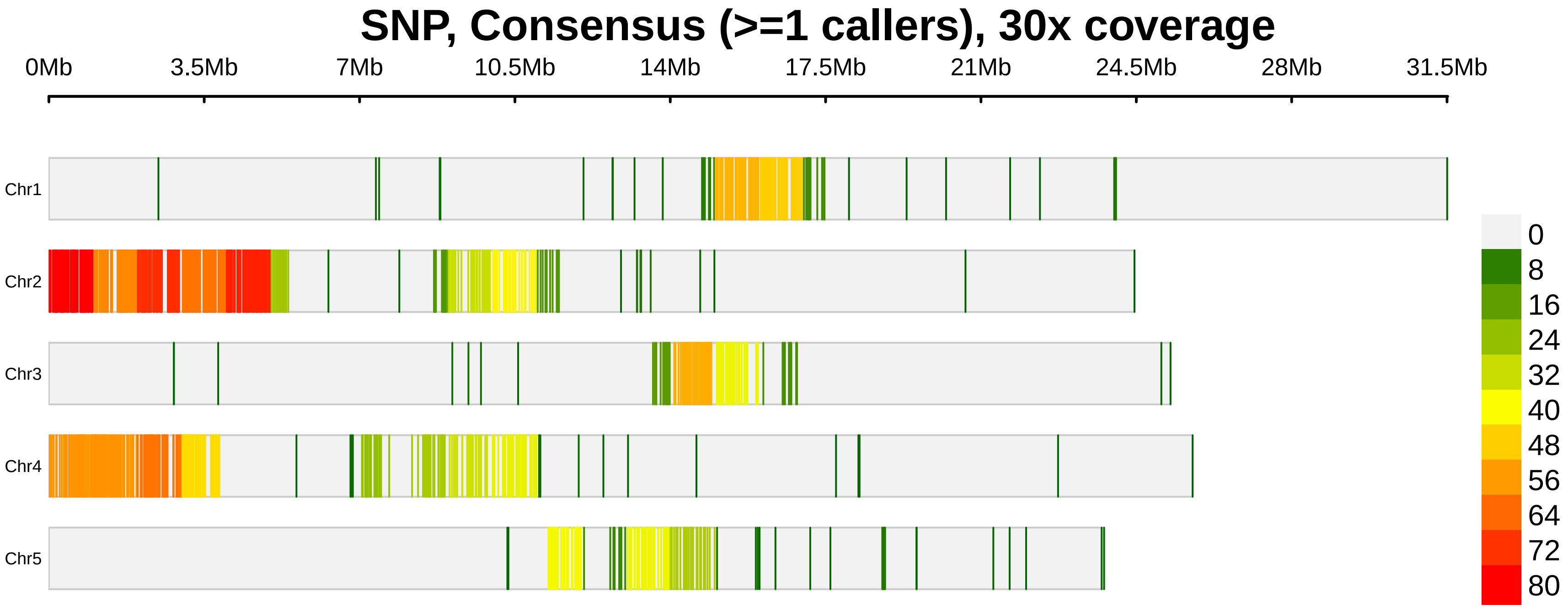
SNP, Consensus (>=1 callers), 30x coverage
0Mb
3.5Mb
7Mb
10.5Mb
14Mb
17.5Mb
21Mb
24.5Mb
28Mb
31.5Mb
Chr1
Chr2
Chr3
Chr4
Chr5
0
8
16
24
32
40
48
56
64
72
80

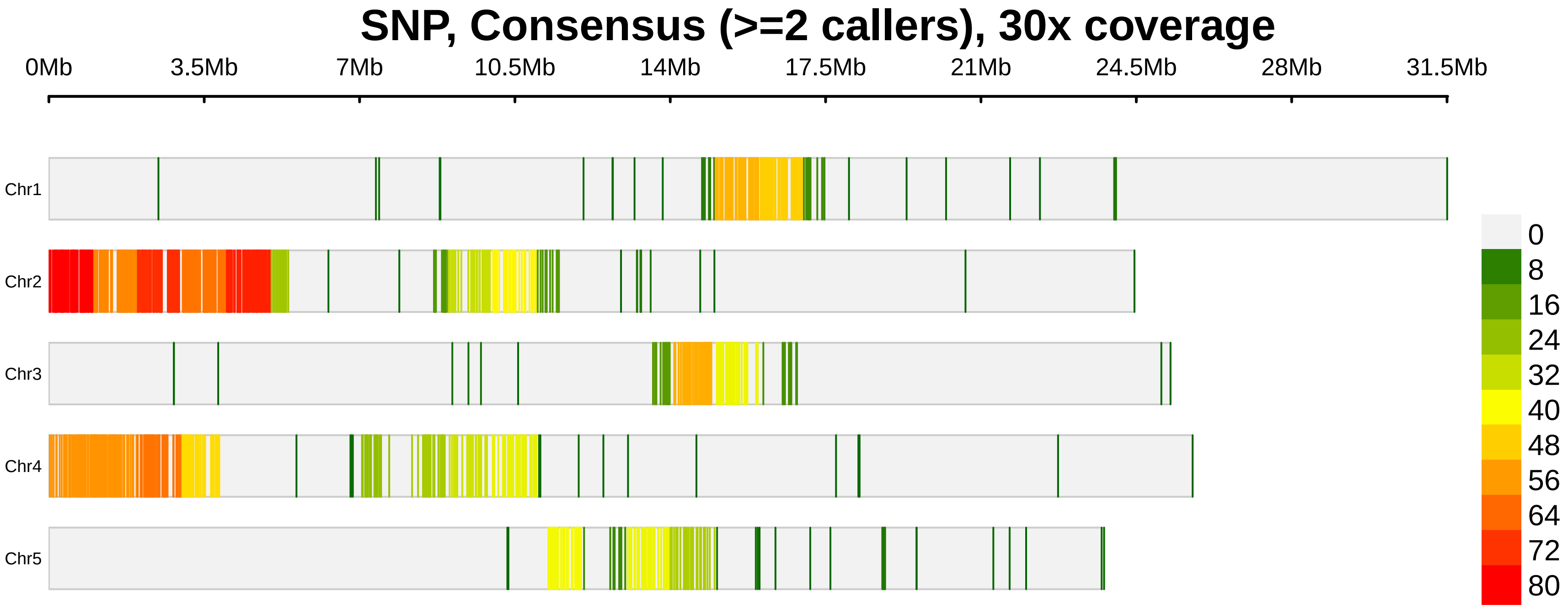
SNP, Consensus (>=2 callers), 30x coverage
0Mb
3.5Mb
7Mb
10.5Mb
14Mb
17.5Mb
21Mb
24.5Mb
28Mb
31.5Mb
Chr1
Chr2
Chr3
Chr4
Chr5
0
8
16
24
32
40
48
56
64
72
80

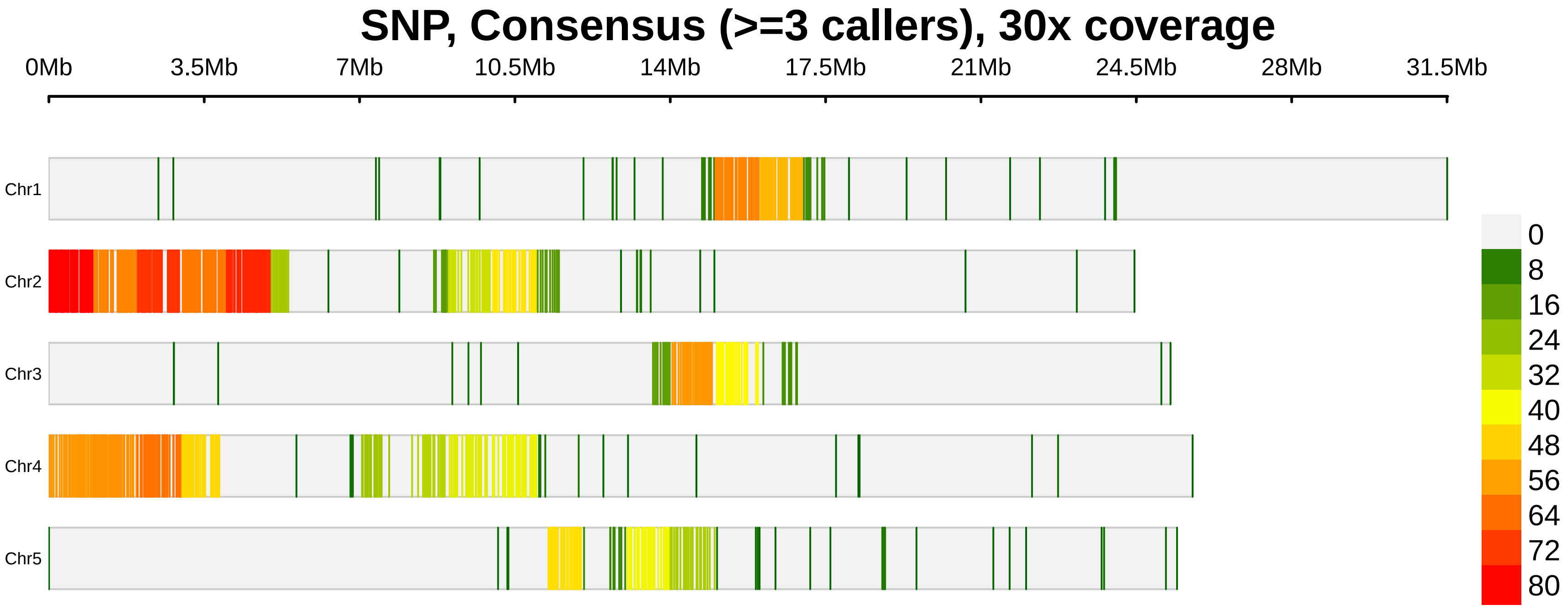
SNP, Consensus (>=3 callers), 30x coverage
0Mb
3.5Mb
7Mb
10.5Mb
14Mb
17.5Mb
21Mb
24.5Mb
28Mb
31.5Mb
Chr1
Chr2
Chr3
Chr4
Chr5
0
8
16
24
32
40
48
56
64
72
80

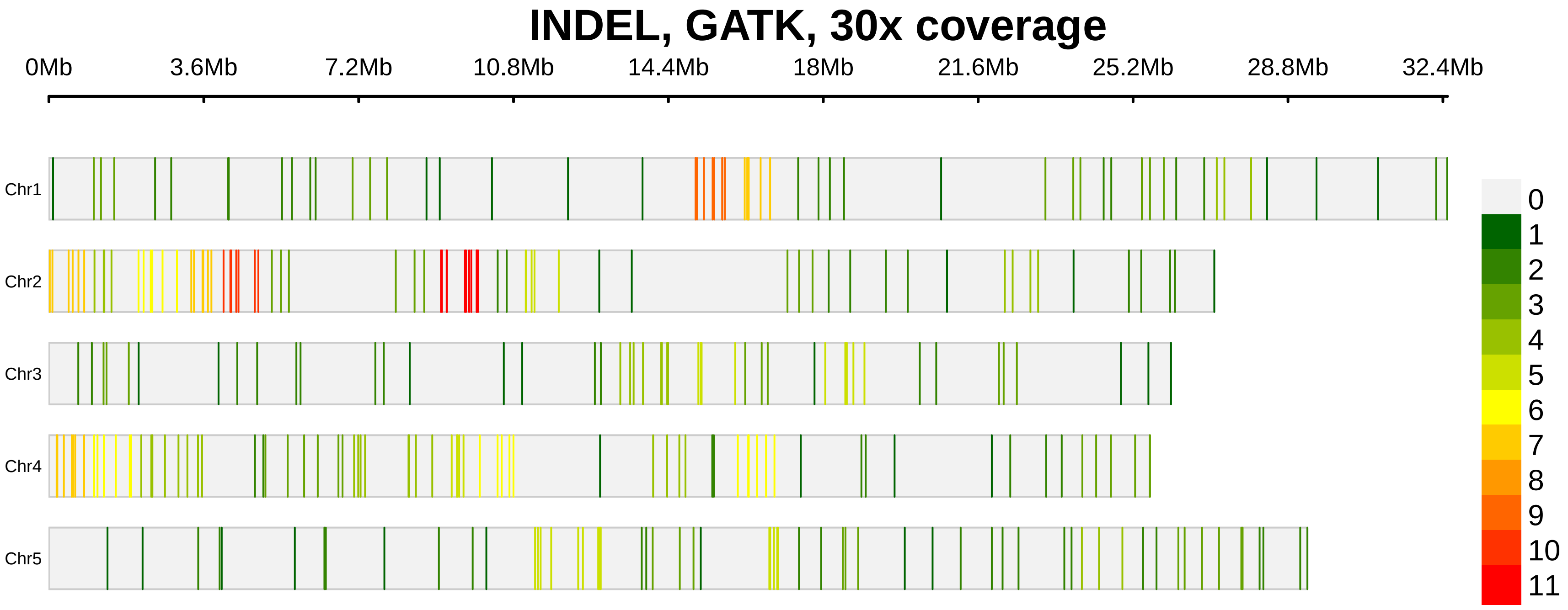
INDEL, GATK, 30x coverage
0Mb
3.6Mb
7.2Mb
10.8Mb
14.4Mb
18Mb
21.6Mb
25.2Mb
28.8Mb
32.4Mb
Chr1
Chr2
Chr3
Chr4
Chr5
0
1
2
3
4
5
6
7
8
9
10
11

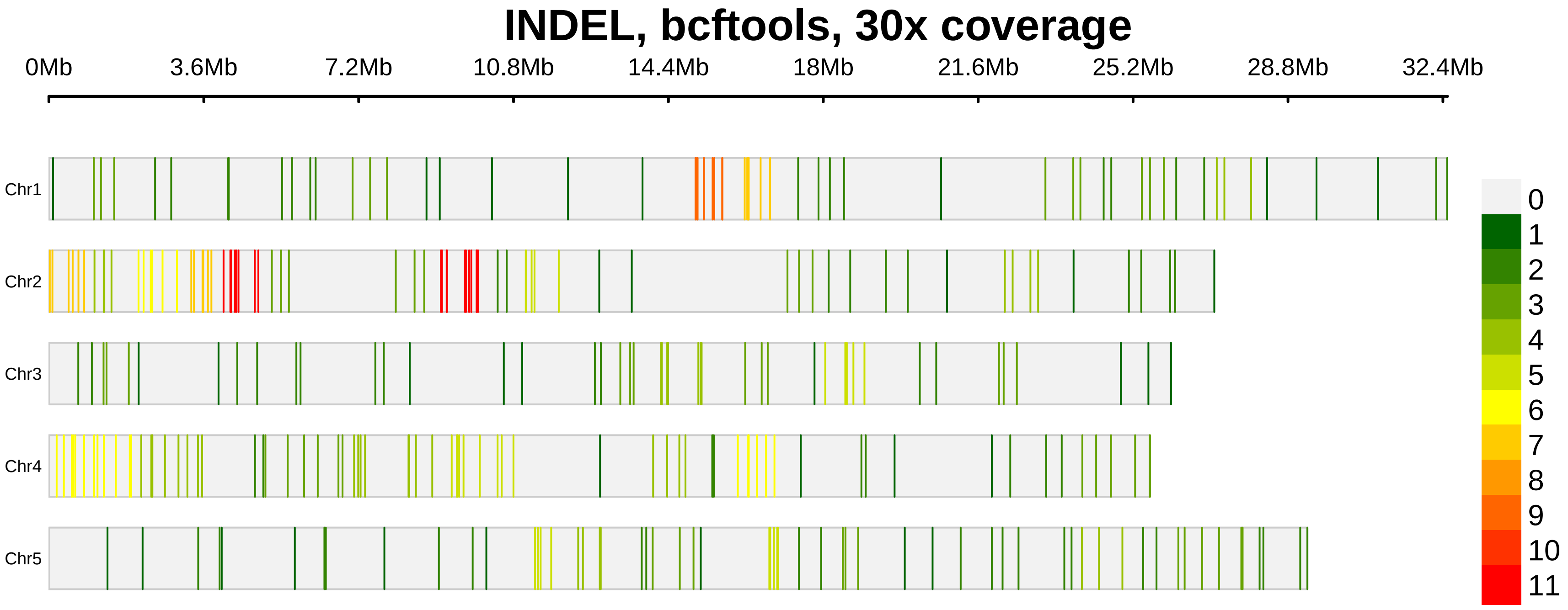
INDEL, bcftools, 30x coverage
0Mb
3.6Mb
7.2Mb
10.8Mb
14.4Mb
18Mb
21.6Mb
25.2Mb
28.8Mb
32.4Mb
Chr1
Chr2
Chr3
Chr4
Chr5
0
1
2
3
4
5
6
7
8
9
10
11

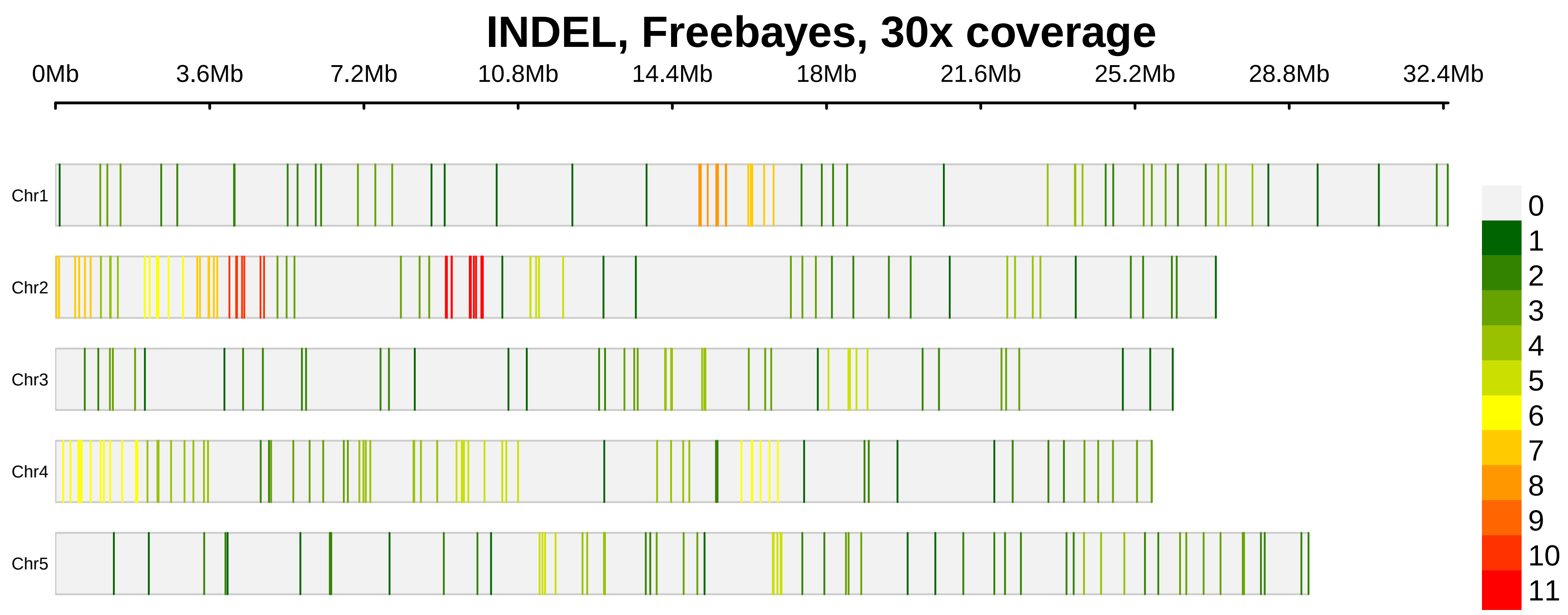
INDEL, Freebayes, 30x coverage
0Mb
3.6Mb
7.2Mb
10.8Mb
14.4Mb
18Mb
21.6Mb
25.2Mb
28.8Mb
32.4Mb
Chr1
Chr2
Chr3
Chr4
Chr5
0
1
2
3
4
5
6
7
8
9
10
11

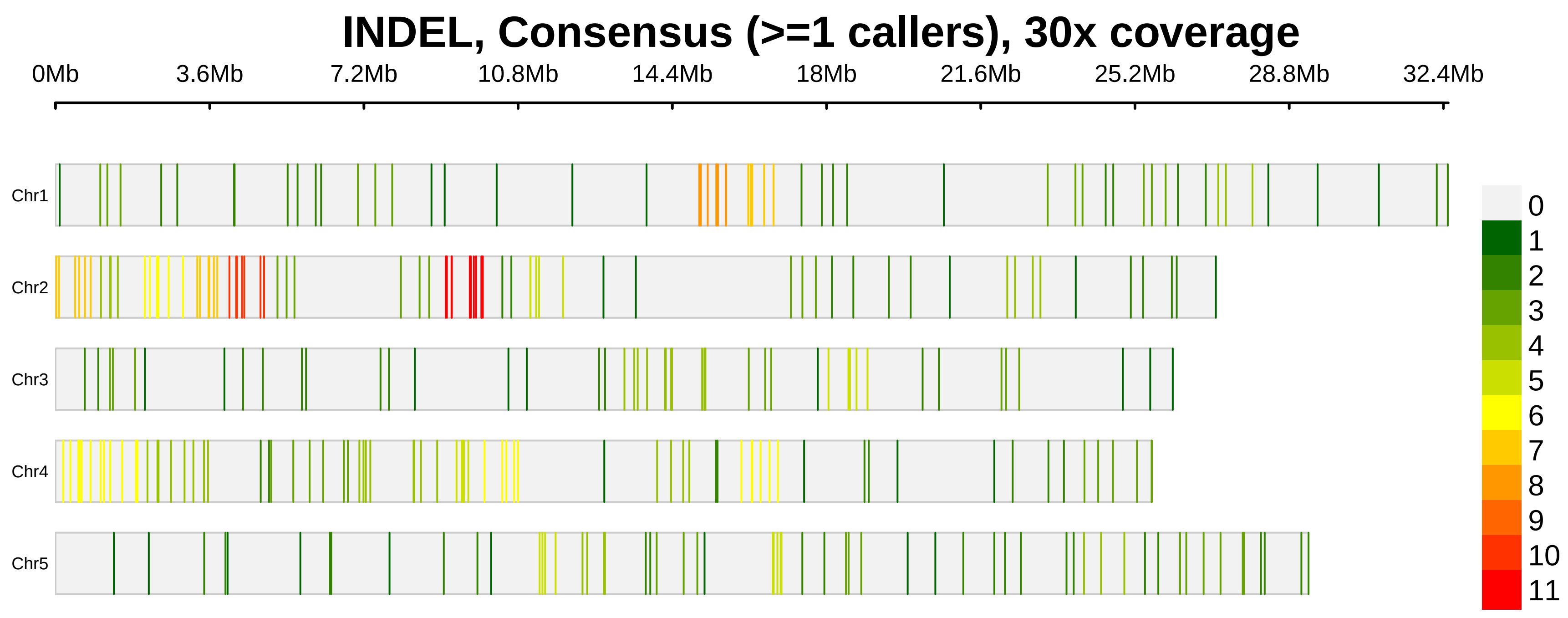
INDEL, Consensus (>=1 callers), 30x coverage
0Mb
3.6Mb
7.2Mb
10.8Mb
14.4Mb
18Mb
21.6Mb
25.2Mb
28.8Mb
32.4Mb
Chr1
Chr2
Chr3
Chr4
Chr5
0
1
2
3
4
5
6
7
8
9
10
11

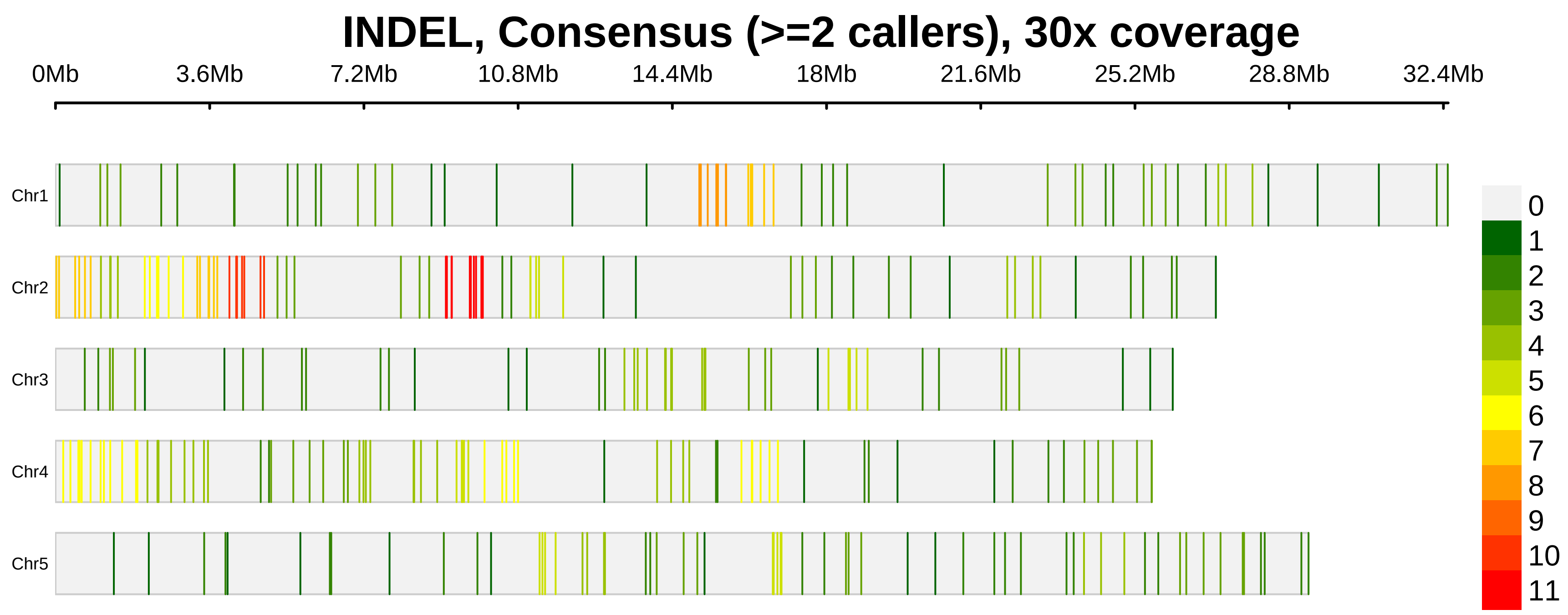
INDEL, Consensus (>=2 callers), 30x coverage
0Mb
3.6Mb
7.2Mb
10.8Mb
14.4Mb
18Mb
21.6Mb
25.2Mb
28.8Mb
32.4Mb
Chr1
Chr2
Chr3
Chr4
Chr5
0
1
2
3
4
5
6
7
8
9
10
11

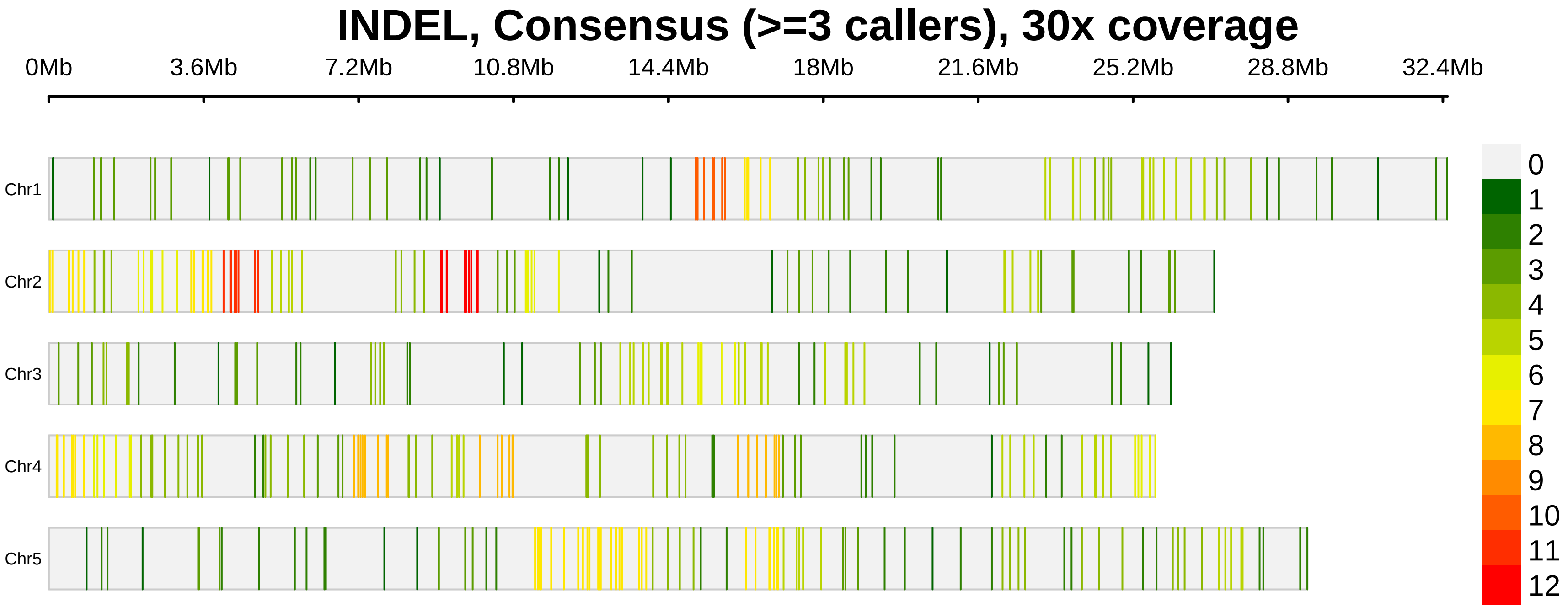
INDEL, Consensus (>=3 callers), 30x coverage
0Mb
3.6Mb
7.2Mb
10.8Mb
14.4Mb
18Mb
21.6Mb
25.2Mb
28.8Mb
32.4Mb
Chr1
Chr2
Chr3
Chr4
Chr5
0
1
2
3
4
5
6
7
8
9
10
11
12

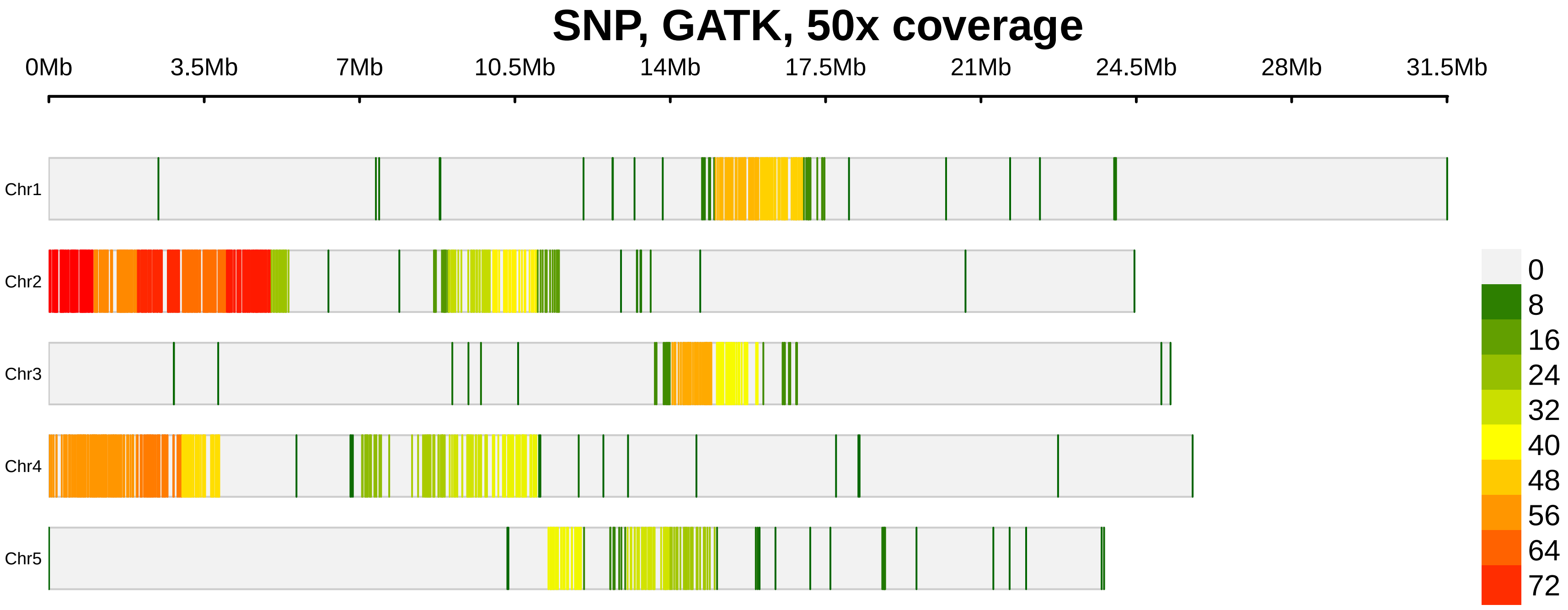

SNP, GATK, 50x coverage
0Mb
3.5Mb
7Mb
10.5Mb
14Mb
17.5Mb
21Mb
24.5Mb
28Mb
31.5Mb
Chr1
Chr2
Chr3
Chr4
Chr5
0
8
16
24
32
40
48
56
64
72

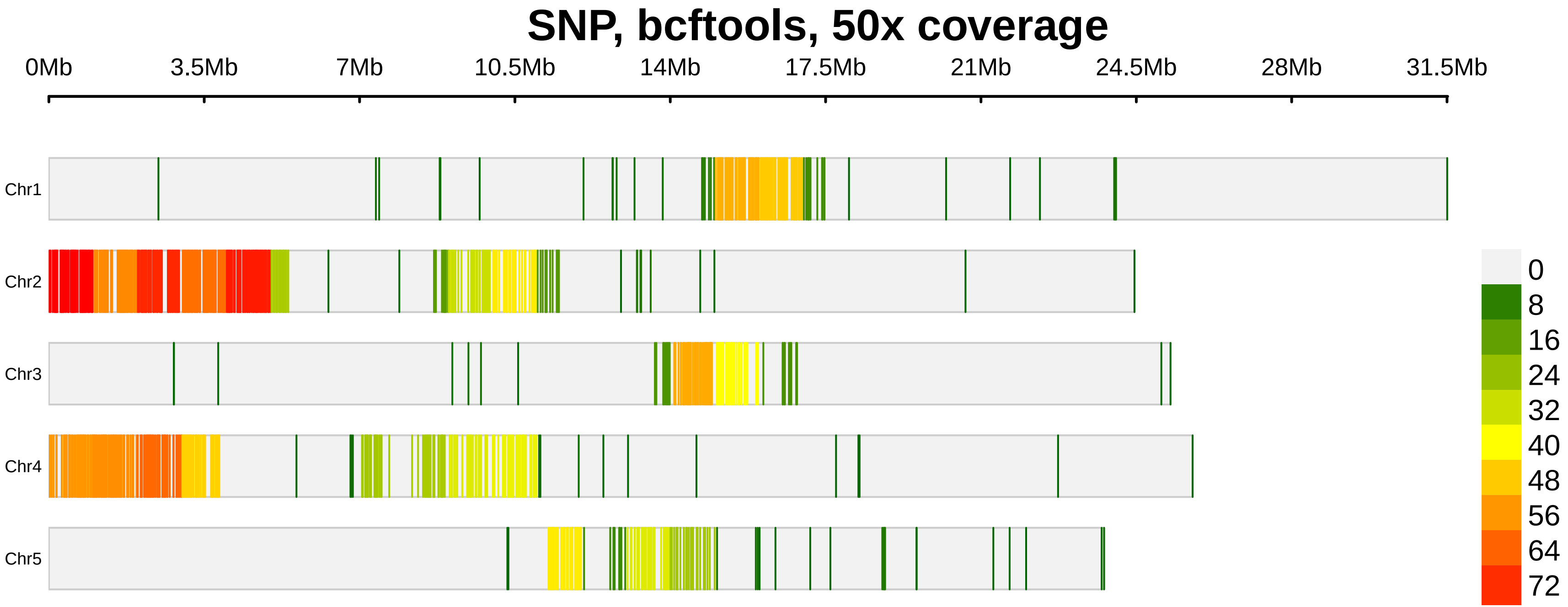

SNP, bcftools, 50x coverage
0Mb
3.5Mb
7Mb
10.5Mb
14Mb
17.5Mb
21Mb
24.5Mb
28Mb
31.5Mb
Chr1
Chr2
Chr3
Chr4
Chr5
0
8
16
24
32
40
48
56
64
72

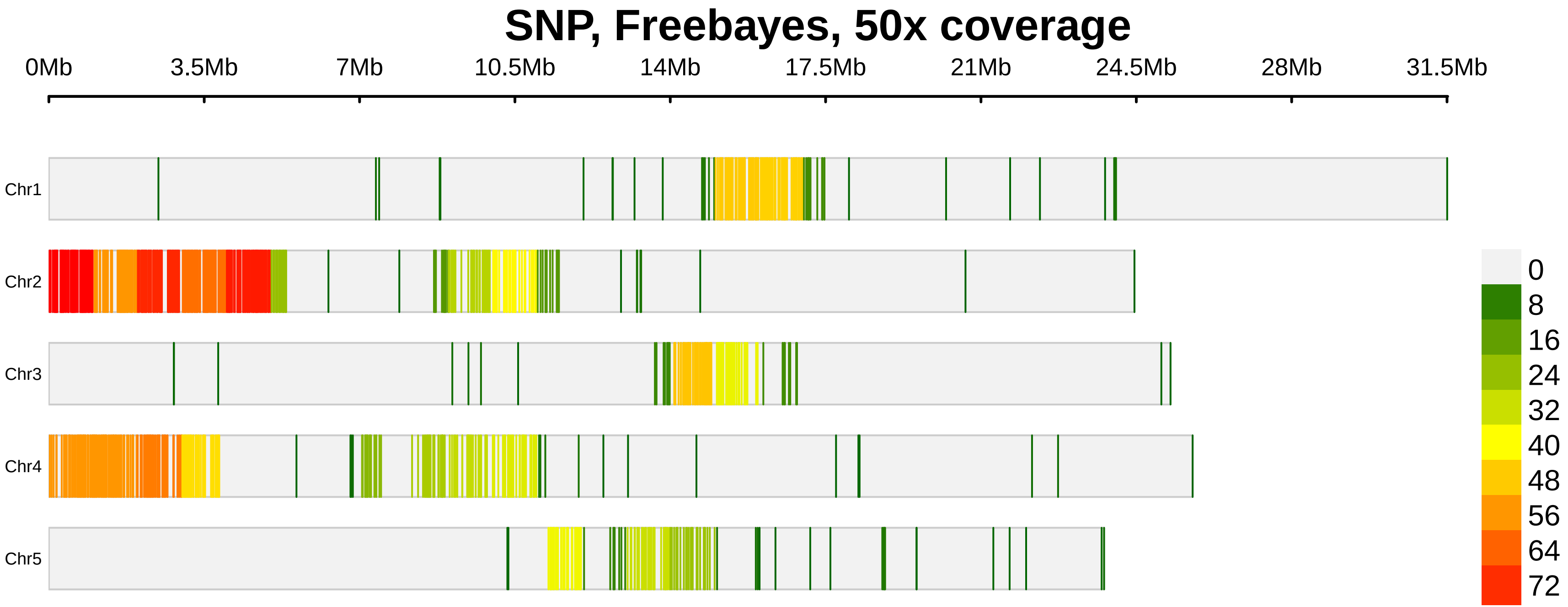

SNP, Freebayes, 50x coverage
0Mb
3.5Mb
7Mb
10.5Mb
14Mb
17.5Mb
21Mb
24.5Mb
28Mb
31.5Mb
Chr1
Chr2
Chr3
Chr4
Chr5
0
8
16
24
32
40
48
56
64
72

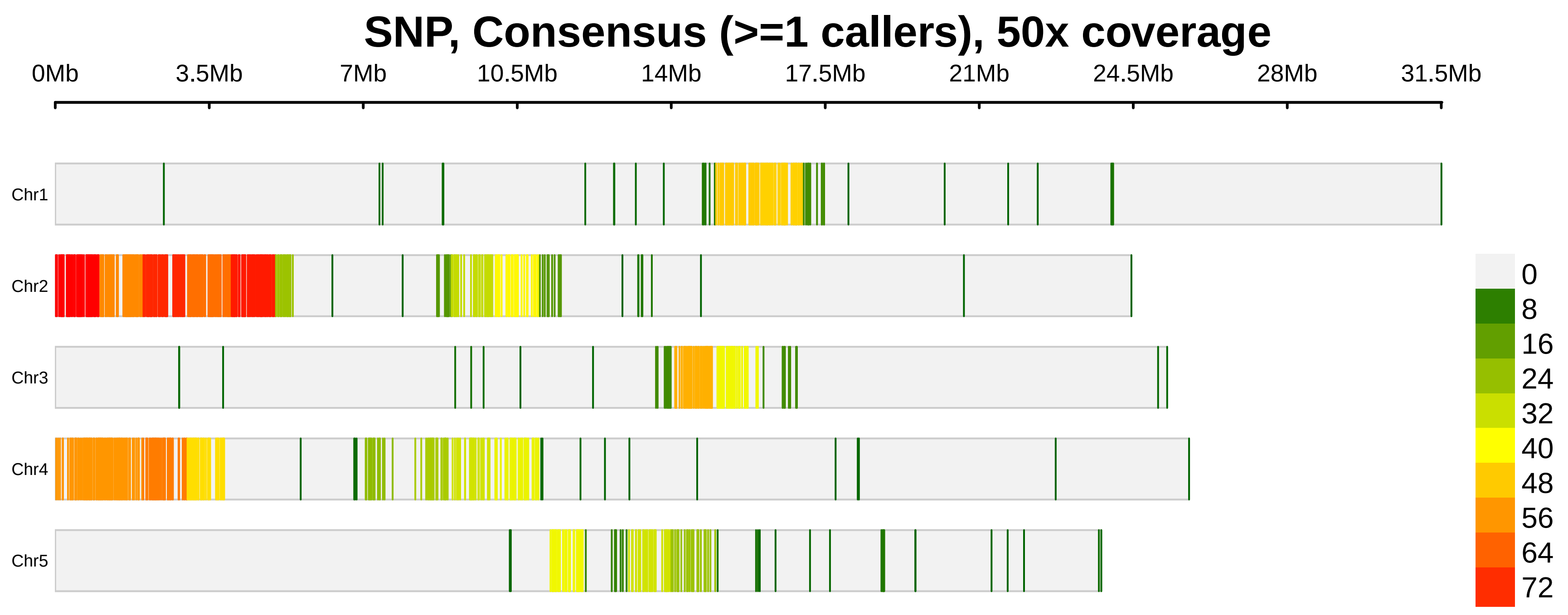
SNP, Consensus (>=1 callers), 50x coverage
0Mb
3.5Mb
7Mb
10.5Mb
14Mb
17.5Mb
21Mb
24.5Mb
28Mb
31.5Mb
Chr1
Chr2
Chr3
Chr4
Chr5
0
8
16
24
32
40
48
56
64
72

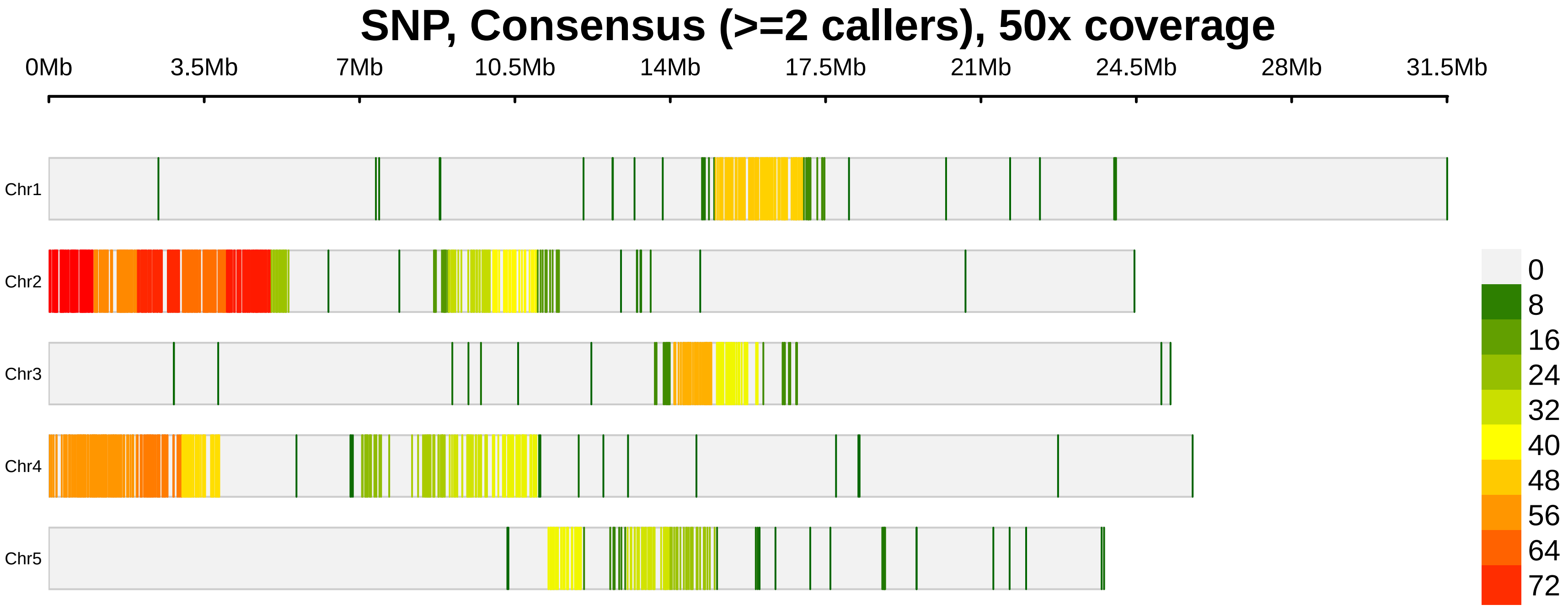

SNP, Consensus (>=2 callers), 50x coverage
0Mb
3.5Mb
7Mb
10.5Mb
14Mb
17.5Mb
21Mb
24.5Mb
28Mb
31.5Mb
Chr1
Chr2
Chr3
Chr4
Chr5
0
8
16
24
32
40
48
56
64
72

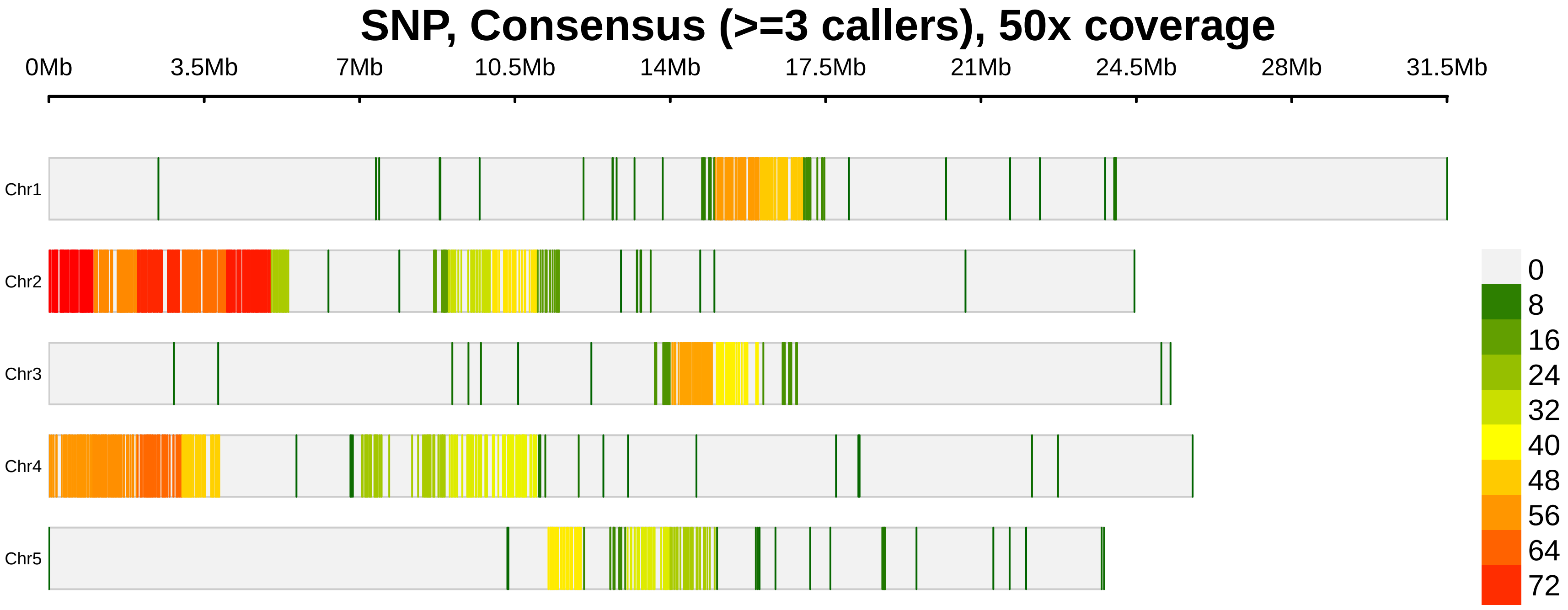

SNP, Consensus (>=3 callers), 50x coverage
0Mb
3.5Mb
7Mb
10.5Mb
14Mb
17.5Mb
21Mb
24.5Mb
28Mb
31.5Mb
Chr1
Chr2
Chr3
Chr4
Chr5
0
8
16
24
32
40
48
56
64
72

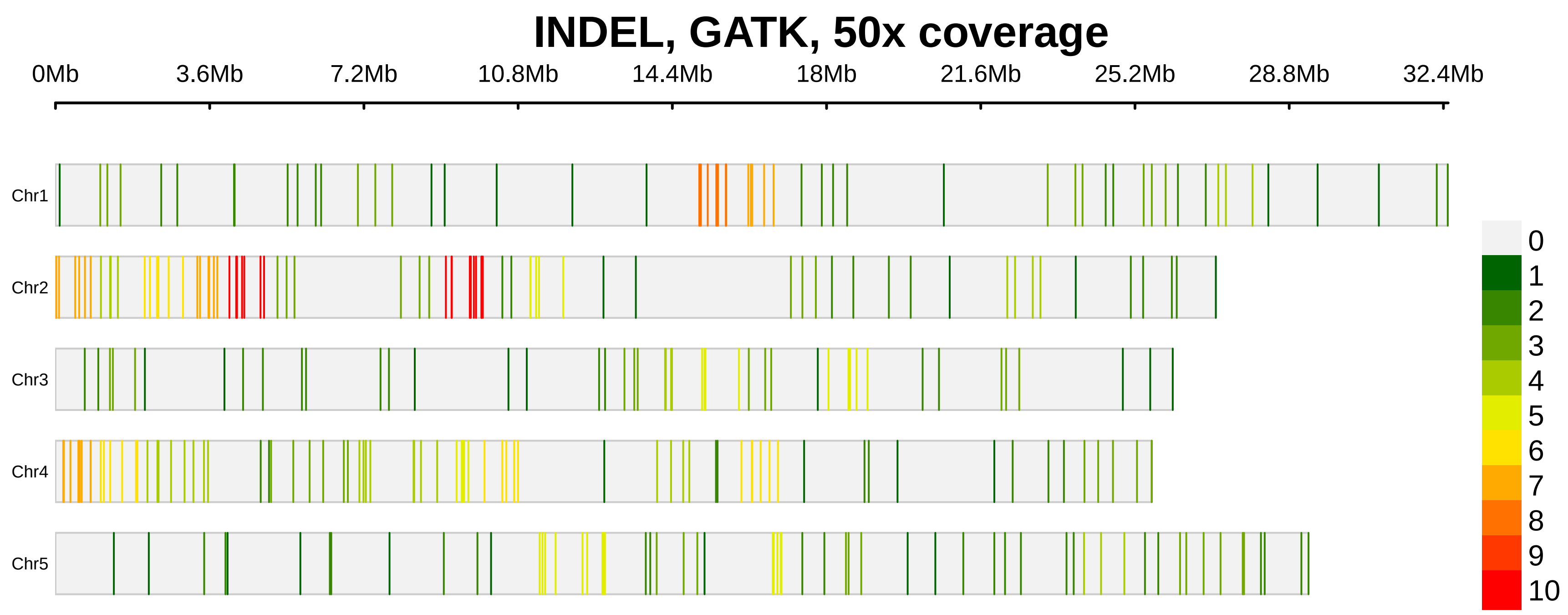

INDEL, GATK, 50x coverage
0Mb
3.6Mb
7.2Mb
10.8Mb
14.4Mb
18Mb
21.6Mb
25.2Mb
28.8Mb
32.4Mb
Chr1
Chr2
Chr3
Chr4
Chr5
0
1
2
3
4
5
6
7
8
9
10

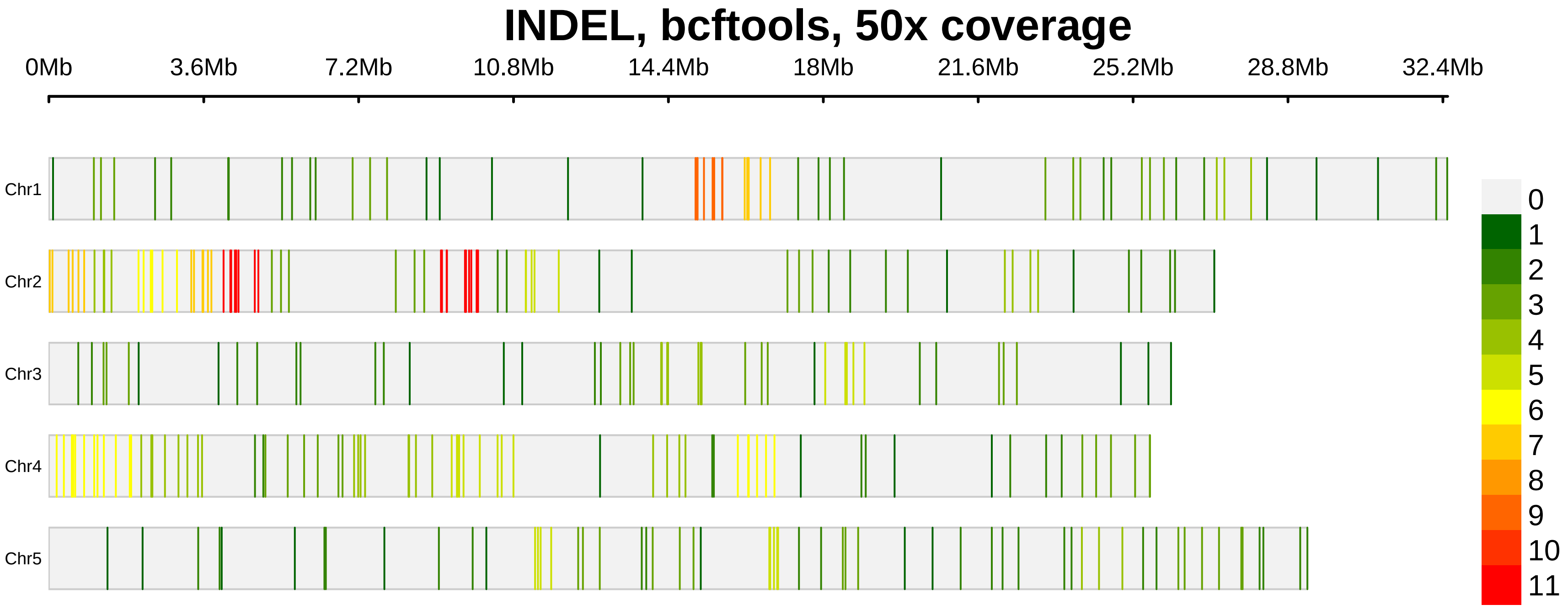

INDEL, bcftools, 50x coverage
0Mb
3.6Mb
7.2Mb
10.8Mb
14.4Mb
18Mb
21.6Mb
25.2Mb
28.8Mb
32.4Mb
Chr1
Chr2
Chr3
Chr4
Chr5
0
1
2
3
4
5
6
7
8
9
10
11

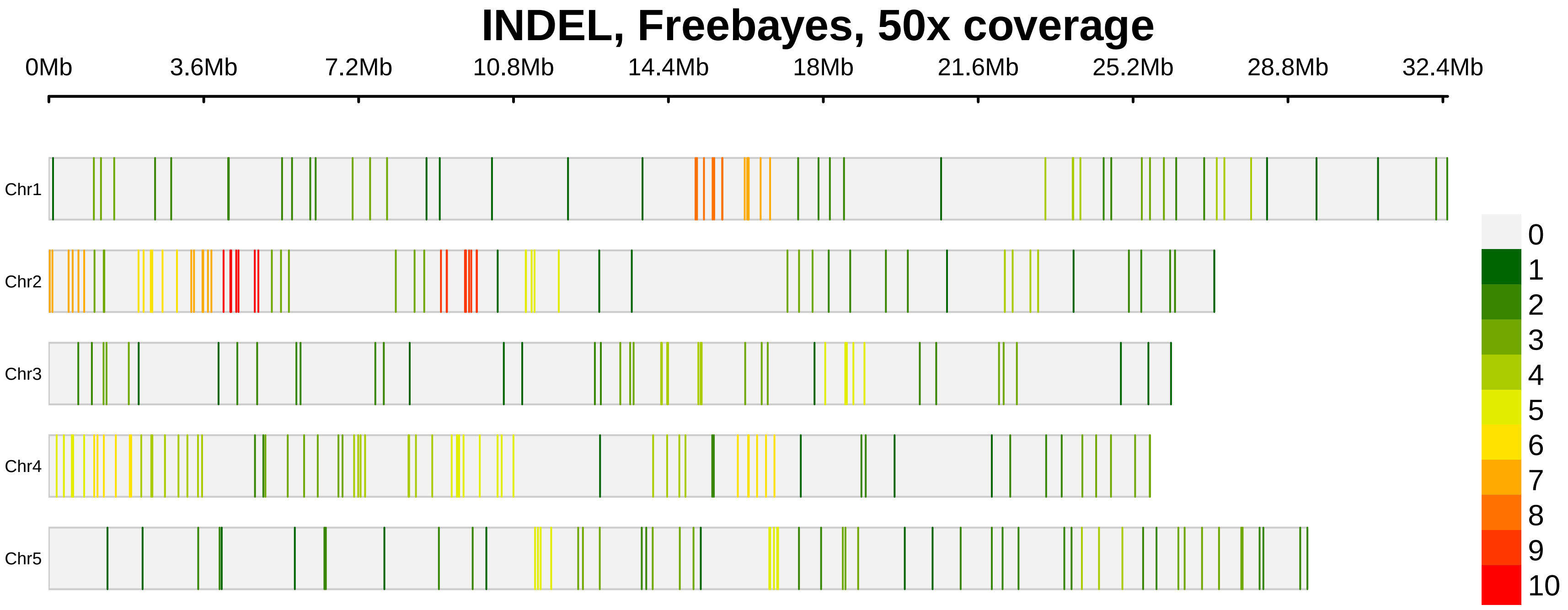
INDEL, Freebayes, 50x coverage
0Mb
3.6Mb
7.2Mb
10.8Mb
14.4Mb
18Mb
21.6Mb
25.2Mb
28.8Mb
32.4Mb
Chr1
Chr2
Chr3
Chr4
Chr5
0
1
2
3
4
5
6
7
8
9
10

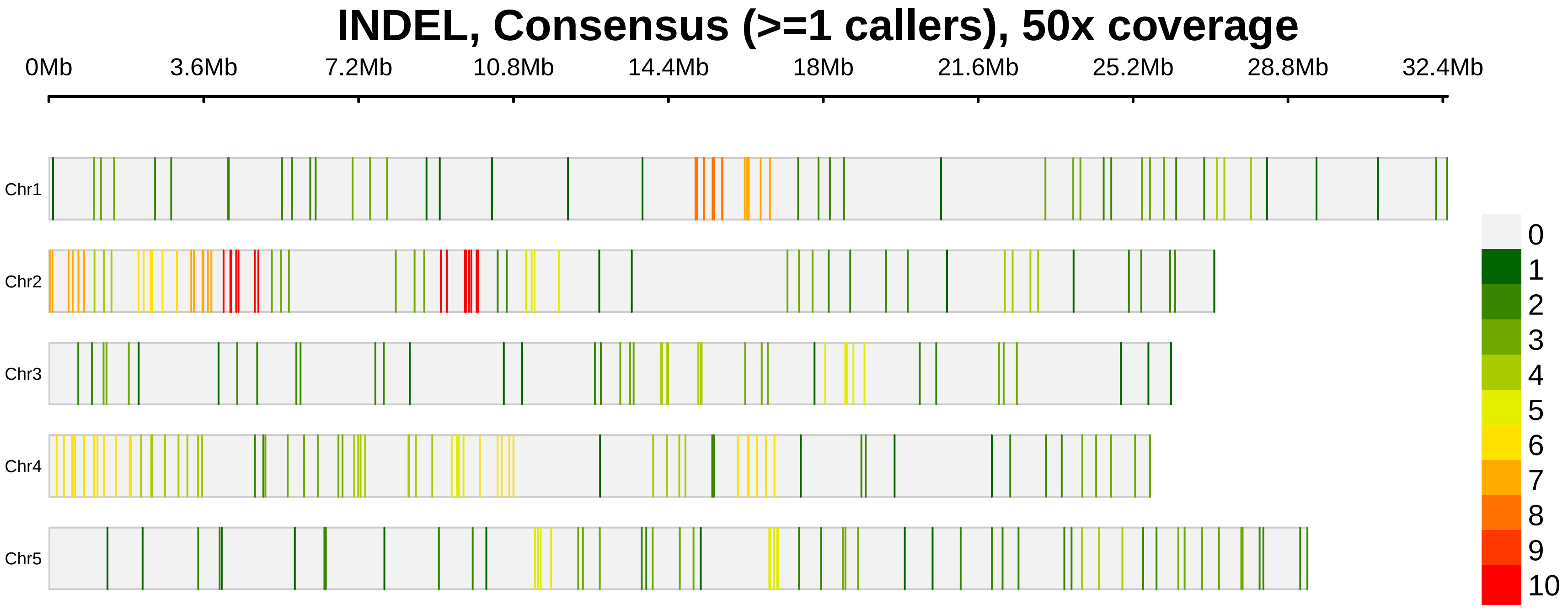
INDEL, Consensus (>=1 callers), 50x coverage
0Mb
3.6Mb
7.2Mb
10.8Mb
14.4Mb
18Mb
21.6Mb
25.2Mb
28.8Mb
32.4Mb
Chr1
Chr2
Chr3
Chr4
Chr5
0
1
2
3
4
5
6
7
8
9
10

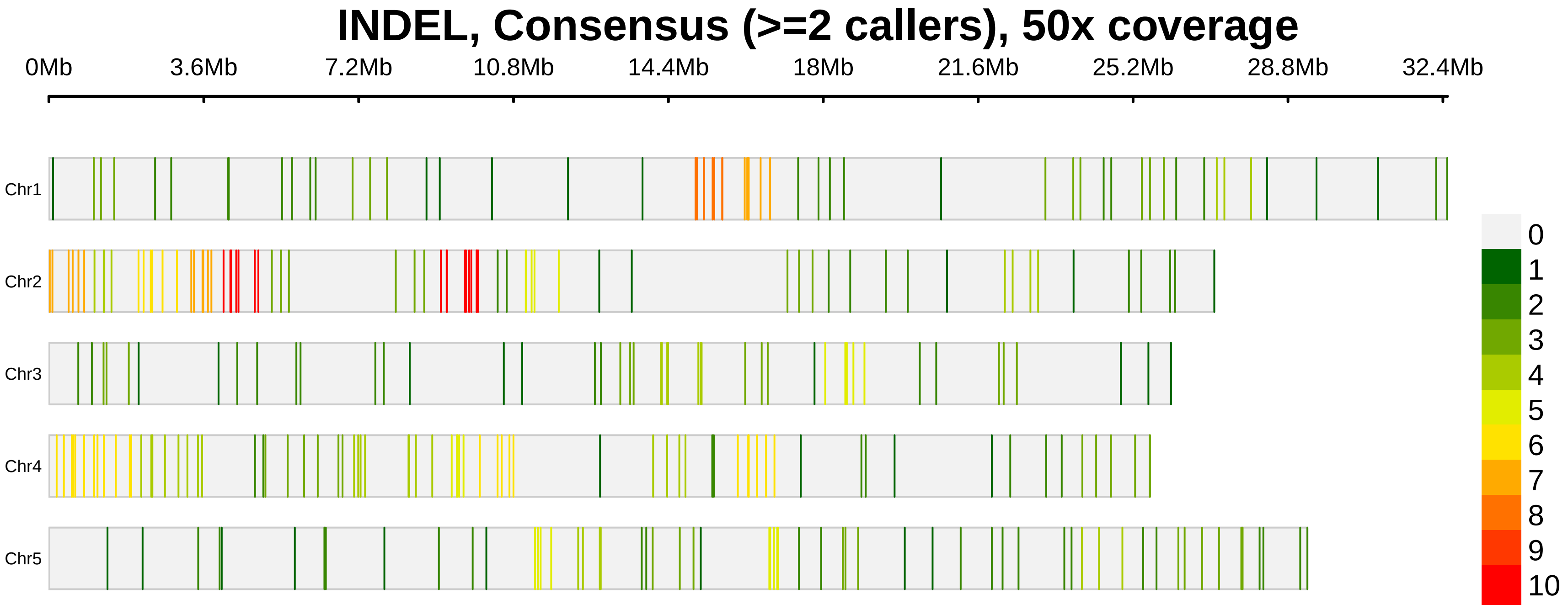
INDEL, Consensus (>=2 callers), 50x coverage
0Mb
3.6Mb
7.2Mb
10.8Mb
14.4Mb
18Mb
21.6Mb
25.2Mb
28.8Mb
32.4Mb
Chr1
Chr2
Chr3
Chr4
Chr5
0
1
2
3
4
5
6
7
8
9
10

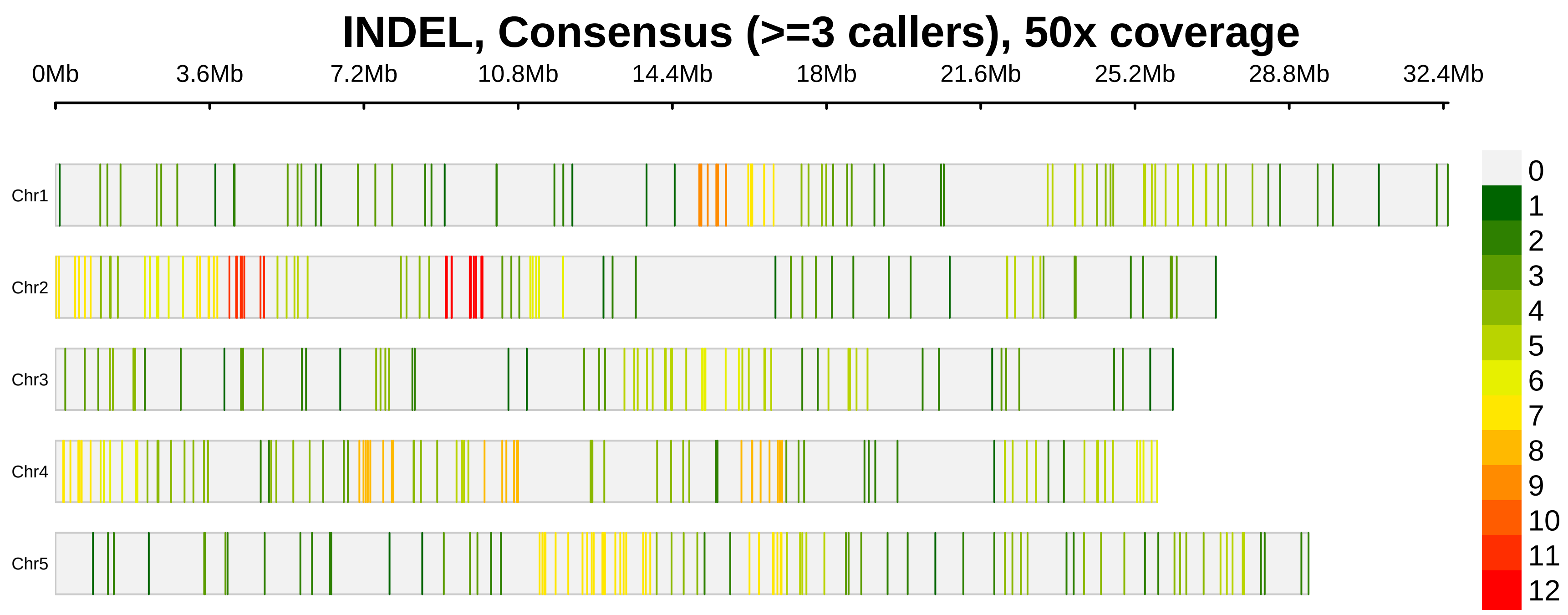

INDEL, Consensus (>=3 callers), 50x coverage
0Mb
3.6Mb
7.2Mb
10.8Mb
14.4Mb
18Mb
21.6Mb
25.2Mb
28.8Mb
32.4Mb
Chr1
Chr2
Chr3
Chr4
Chr5
0
1
2
3
4
5
6
7
8
9
10
11
12
